# From 15 Minutes to 15 Seconds: How the Delta Variant Changed the Risk of Exposure to COVID-19. A Comparative Epidemiological Investigation Using Community Mobility Data From the Metropolitan Area of Genoa, Italy

**DOI:** 10.3389/fpubh.2022.872698

**Published:** 2022-07-05

**Authors:** Cristina Oliva, Giampiero Favato

**Affiliations:** Institute of Leadership and Management in Health (ILMH), Kingston University, London, United Kingdom

**Keywords:** delta, variant, risk, exposure, game theory, response, COVID-19

## Abstract

The Delta variant became dominant during the second wave of the Covid-19 pandemic due to its competitive advantage, the ability to reduce close contact duration from minutes to seconds, and, consequently, increase the risk of exposure to COVID-19. We used game theory to model the most effective public health response to this new threat. We compared the absolute and relative risk of exposure to COVID-19 before and after the emergence of the Delta variant. The absolute risk of exposure was defined as the product of crowding (people within a six feet distance) and visit duration. Our epidemiological investigation used aggregated and anonymized mobility data from Google Maps to estimate the visit duration for 808 premises in the metropolitan area of Genoa, Italy, in June 2021. The relative risk of exposure was obtained by dividing the risk of exposure of each activity by the lowest value (gas stations = 1). The median absolute risk of exposure to COVID-19 increased by sixty-fold in the first semester of 2021, while the relative risk did not significantly differ from the risk of exposure to the ancestral form of Covid-19 (5.9 in 2021 vs. 2.5 in 2021). The Delta variant represents an evolution of the game against COVID-19, but it is not a game-changer. The best response is to commit to our original strategy based on population-wide vaccination and social distancing. Unilateral deviations from the dominant strategy could offer COVID-19 a fighting chance against humanity.

## Introduction

The pandemic spread of a virus in naïve populations can select mutations that alter virulence or transmissibility (1). The ancestral form of severe acute respiratory syndrome coronavirus 2 (SARS-CoV-2) that emerged from China in April 2020 was mainly replaced by the B.1.617.2 mutation, or DELTA variant, first detected in India in late 2020, where it is thought to have contributed to the extremely high number of cases during the country's second wave of COVID-19 (2). As of June 2021, it had spread to 74 countries worldwide (3). It later contributed to a third wave in the United Kingdom (4), and the WHO warned in July 2021 that it could have a similar effect elsewhere in Europe (5). The Delta variant rapidly replaced all other SARS-CoV-2 variants due to its “fitness”, the reproductive rate (R_0_), almost double the one observed with the ancestral strain (6).

What was the competitive advantage of the Delta variant? The Delta variant was more transmissible than previously evolved ones (7). Research conducted in the U.K., where the variant accounted for 99% of new Covid cases, suggested that it was about 60% more transmissible than the Alpha variant, which previously dominated (8, 9). Based on CCTV footage, Australian health officials suspect it has been transmitted in “scarily fleeting” encounters of roughly 5 to 10 seconds between people walking past each other in an indoor shopping area in Sydney in at least two instances (10). By reducing the close contact risk from 15 min (10) to 15 seconds, the Delta variant would significantly increase the risk of exposure to COVID-19. Consequently, should public health decision-makers change their response to the Delta variant or commit to the community mitigation measures already in place?

The theory of games can explain how viruses evolve when they compete against one another in a test of evolutionary fitness and predict which strategy will dominate this contest (11).

**Figure 1:**
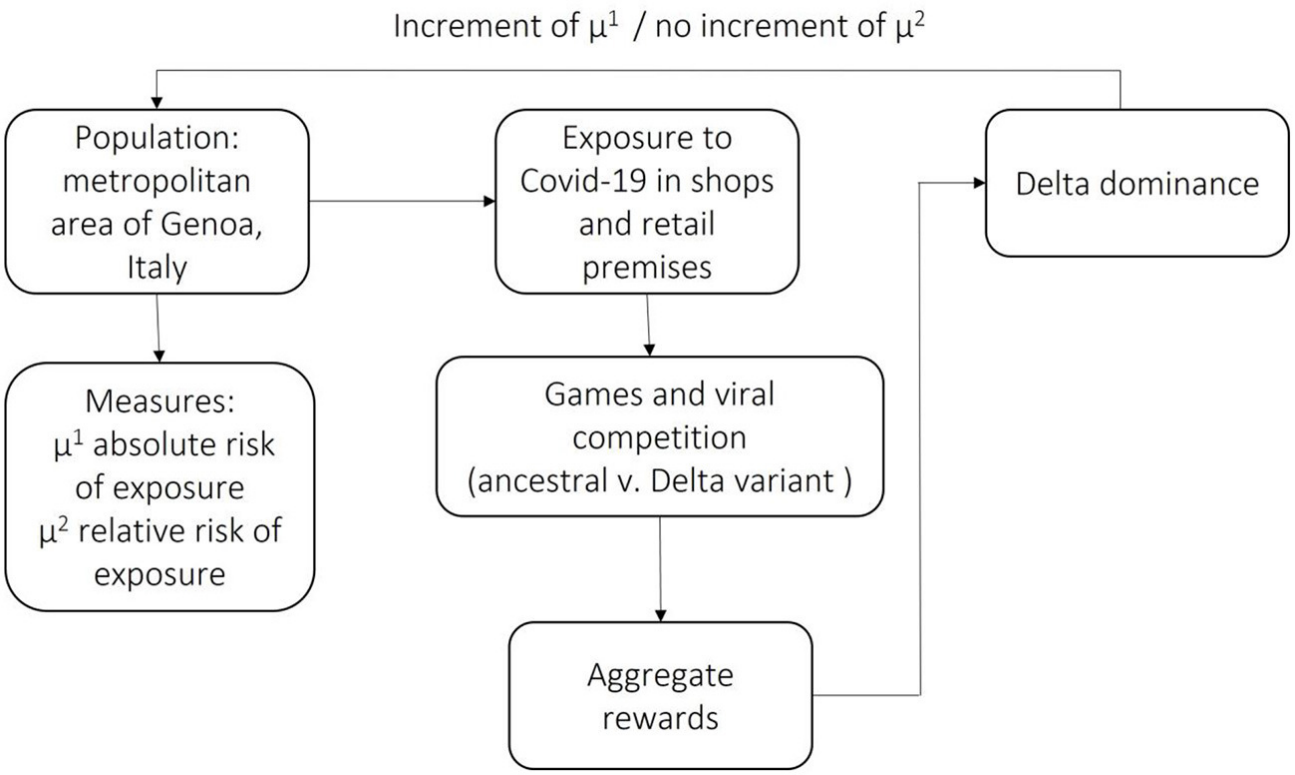
Delta variant evolutionary game.

To understand how game theory might help understand viral mutation when differing strategies are associated with different underlying genetics, we illustrated in Figure 1 an evolutionary game summarized in three main steps: “*meet, compete and mutate*” (12), graphically represented in Figure 1. First, consider a game where a defined population (the residents of the metropolitan area of Genoa, Italy) and the COVID-19 virus always play the same Tit-for-Tat strategy. The success of the population strategy is measured according to the population absolute (μ^1^) and relative (μ^2^) risk of exposure to the viral infection. Now, suppose that the ancestral form of COVID-19 competes with the Delta variant, which plays the Always Cheat strategy (i.e., they try to cheat everyone they meet). The Delta variant will soon dominate and completely replace the ancestral form, given its competitive advantage on the reproductive rate. The Delta's dominance would increase the population's μ^1^, the absolute risk of exposure to viral infection. Should the population adapt its response to the cheater (Delta variant) or maintain the original Tit-for-Tat strategy? If the game is a stable evolutionary game, maintaining the Tit-for-Tat strategy will prove more successful, and the cheaters will eventually lose out (13).

Our working hypothesis was that the Delta variant was a new round of the COVID-19 evolutionary game, a stable form of the “prisoner's dilemma” (14).

The first condition to accept the hypothesis is that the “cheaters” (the Delta variant) must displace the ancestral form of COVID-19 completely. Latest estimates confirmed that by the end of August 2021, the Delta variant represented 90% of all SARS-CoV-2 viruses circulating in the European Union (15).

We needed to confirm the second condition, that the fitness of the Delta variant relative to the ancestral COVID-19 had to be frequency-dependent because the model predicts that cheaters will show their greatest fitness advantage when they are rare relative to the co-operators (16). The primary aim of our study was to confirm the early fitness advantage of the Delta variant. We compared the absolute risk of viral exposure (μ^1^) by retail premises estimated in June 2021 with comparable values obtained in December 2020, mainly attributable to the ancestral form of COVID-19. Both sets of data were collected and analyzed following an identical method, which used for the first time mobility data on the average time spent by customers in a given location from a sample of retail premises in Genoa's metropolitan area (Italy). The secondary objective was to compare the population's relative risk of exposure (μ^2^), obtained by setting the lowest risk by premise = 1, before and after the Delta variant. The dominance of the new variant should not increase the population's relative risk attributable to COVID-19, assuming that the current mitigation strategies (Tit-for-Tat) are maintained.

If these two criteria were met, the Delta variant scenario would be consistent with the prisoner's dilemma. Consequently, the stable evolutionary theory could help us understand the Covid-19 variants' dynamics. Finally, but most importantly, it would confirm that vaccination, mask protection, and social distancing continue to be the dominant public health strategy to mitigate the pandemic's health and social impact.

The selection of the Delta variant is described as a moment of the viral evolutionary game. The process architecture is a simple *meet, mate and mutate* game. The self-contained population (metropolitan area of Genoa, Italy) is defined by two measures (absolute and relative risk of exposure). Exposure to COVID-19 (*meet*) generates random pairs for every encounter between prey and predators (ancestral virus and Delta variant). Delta variant does not co-operate and adopts the “*always cheat”* strategy. The initial reward allows Delta to become the dominant variant (*mutate*). In a stable evolutionary game, the dominance of the cheaters leads to an immediate advantage (increment of μ^1^) but does not change the game (μ^2^ does not increase).

## Materials and Methods

The manuscript was prepared in adherence to the STROBE (STrengthening the Reporting of OBservational studies in Epidemiology) reporting guidelines.

### New Data: Median Visit Duration Time by Retail Activity

Since June 2020, Google has been showing searchers how long they can expect to be at a specific store or venue based on the crowdsourced data from users who travel to specific stores. Visit duration estimates are based on patterns of customer visits over the past several weeks. Google does not report visit duration for those activities that do not generate reliable daily mobility data.

This new feature shows how much time customers typically spend in a specific store. Visit duration is based on customer visit patterns over the past several weeks and is expressed in units of time (minutes) (17). Some retail stores show the visit duration as a mean value (e.g., 15 min), while others as a range (e.g. 30–60 min). Visit duration times are publicly available on Google Maps.

Since the Delta variant could have reduced the close contact time to just a few seconds, we obtained a univocal measure of visit duration time by including the mean values (e.g., 15 min) or the lower limit of each range (e.g., 30 min) for each retail activity. Then, as input to the risk of exposure, we divided the visit duration (in seconds) by 15.

Google reports median visit duration in minutes as a range (upper and lower limits) for 11 out of 14 retail activities. At the same time, grocery shops, pharmacies and gas stations display only the median average visit duration. Thus, while the dispersion is narrow for in-and-out daily activities (such as grocery shopping or filling up the car at a gas station), the variance of time spent in other activities can be better expressed as a range. For instance, a quick espresso at the counter takes much less than an animated debate about football in front of an aperitive in a coffee shop.

The drastic reduction of time to close contact attributable to the Delta variant imposed a methodological choice regarding visit duration. Rather than a range, we used the shortest visit duration reported by Google as the contact time to calculate the risk of exposure. Consequently, the risk of exposure to the Delta variant by retail activity estimated by this research is fully comparable to the “lower limit of the range” scenario of the risk of exposure to the ancestral COVID-19 reported in the previously published study (18).

During the week from 28/06/2021 to 02/07/2021, we manually collected median visit duration data for all the retail activities, banks and public offices located by Google Maps in the metropolitan area of Genoa, Italy, which reported the visit duration time (*n* = 808). The sample was then clustered into 14 everyday activities, from grocery shopping to the post office. Data were collected from all the Genoa metropolitan area retail activities visible on Google Maps and reported visit duration times. Google does not report the visit duration for activities that do not generate reliable daily mobility data.

Interpreting mobility data in metropolitan areas requires an in-depth understanding of the urbanism and road mapping of the selected area. The choice of the location was determined by the fact that one of the Authors was born and raised in the metropolitan area of Genoa. The data collected for the study are available in the online Supplementary Material.

### Ethical Considerations

No data privacy issue is associated with the mobility data used to inform our risk model. Google Map publicly provides the duration of visit data by premise in a strictly aggregated and anonymised form. No personally identifiable information, such as an individual's location, contacts or movement, was made available at any point.

### Outcomes

From the CDC's definition of closed contact (19), we derived a working definition of the risk of exposure to the Delta variant for daily activities:


(1) $$\begin{array}{r} \text{Risk of exposure}=\left[\text{visit duration}\left(\text{seconds}\right)/15\right]\text{ X crowding} \end{array}$$

At the time of data collection and analysis, the minimum transmission time for the Delta variant was anecdotally estimated to be below 10 seconds: we conservatively used 15 cumulative seconds of exposure at a distance of 6 feet or less (20) as an operational definition for close contact.

Google median visit duration times by individual premise for the sample of n=808 retail premises included in the analysis are reported in the online Supplementary Material.

In Italy, crowding standards (the maximum allowable people per square meter) for retail and office premises represented a key social distancing measure, regulated by law since April 2020 (21). Accordingly, inputs for crowding standards of retail premises were derived from the latest norm in place since June 2021 (22).

We calculated the absolute risk of exposure to the Delta variant as the product of the median visit duration by retail activity expressed in units of time of 15 seconds by the maximum number of people by square meter allowed by the current crowing norm divided by a close contact space of six square feet (approximately 10.4 square meters). We then obtained a relative risk measure by dividing individual exposure risks by a constant equivalent to the lowest risk value observed (gas stations = 1).

The risk of exposure to the Delta variant by retail premises was then compared to exposure to the ancestral form of COVID-19 obtained following the same method but using data collected from the same metropolitan area of Genoa, Italy, in December 2020 (23).

As recommended by CDC, close contact should generally be determined irrespective of whether the contact was wearing respiratory personal protective equipment (PPE) (24).

### Statistical Analysis

We calculated the median visit duration using the statistical software MedCalc Version 20.110 (MedCalc Software Ltd, Ostend Belgium). The choice of median values is consistent with Google's method to calculate mobility data changes across different categories of places (25). Data on visit duration by premise were non-randomized (since we collected all visit duration times available for each retail activity in the Genoa metropolitan area) and non-normally distributed. As discussed earlier, the risk of exposure for each retail activity depended on a single variable (the median visit duration time), while all other parameters were constant. Consequently, we tested the following null hypothesis:

*H*_0_*: Samples come from the same distribution and have the same median*.

Rejecting the null hypothesis would confirm the validity of the estimated parameter (median visit duration) to calculate the absolute and relative risk of exposure to the Delta variant by retail activity.

We used two non-parametric methods to test the fourteen independent, non-normally distributed samples of median visit duration by retail activity.

Firstly, we used the Kruskal-Wallis one-way ANOVA, a non-parametric method for comparing k independent samples. The null hypothesis is that the distributions of k groups are equal. The Kruskal-Wallis test assumes independence of observations, no assumption of normality, and the distributions of the dependent variable must have similar shapes. If these assumptions are met, the test can be interpreted as testing for differences between medians (26).

Secondly, we used the non-parametric Mood's median test as a special Pearson's chi-squared test case. Similarly to the Kruskal-Wallis test, the Mood's test checks whether the medians of two or more groups differ and assumes the same conditions (27). Both tests allow for multiple pair-wise comparisons, which is a desirable feature for estimating the trend of the median visit duration over time. To reduce the risk of type 1 error when making multiple comparisons, *p*-values for pair-wise comparisons were computed using 10,000 Monte Carlo simulations and the Bonferroni correction (significance level: 0.0005) with the aim to reduce the chances of obtaining false-positive results (type I errors) when multiple pair wise tests are performed on a single set of data.

We used both non-parametric tests because the Kruskal-Wallis test is preferable when three or more samples need to be compared. In contrast, Mood's test effectively detects a shift in location for symmetric and heavy-tailed distributions (28).

We then tested the accuracy of the absolute risk of exposure model by using a least square regression of the median visit duration by retail activity against the absolute risk values. Finally, we checked for normality of residuals using the Kolmogorov-Smirnov test for normal distribution with Lilliefors significance correction. Finally, we checked patterns in the scatterplot of standardized residuals v. standardized predicted values for homoscedasticity.

Lastly, we used again both a Bonferroni-adjusted, Monte Carlo resampled, Kruskal-Wallis and a Mood non-parametric method to test the difference in medians of the absolute and relative risk of exposure by retail activity between two different points in time:

December 2020, when the ancestral form of COVID-19 was dominant and June 2021, when the Delta variant was prevalent in Italy (29).

## Results

Table 1 reports the median visit duration by retail activity in the metropolitan area of Genoa, Italy, based on store data extracted from Google Maps on June 28, 2021. The distribution of the retail activities for which Google reports the average duration of visit reflects the priorities of our daily life in a metropolitan area, and it is coherent with the published data collected in December 2020. Food supermarkets (*n* = 201), retail shops (*n* = 91), pharmacies (*n* = 81), post offices (*n* = 65) and banks (*n* = 50) were among the most represented locations in the dataset (60% of total compared to 56% in 2020). Social activities, such as pizza restaurants (*n* = 78), fine dining (*n* = 48), pubs (*n* = 32), fast-food (*n* = 31), and coffee shops (*n* = 55), represented 30% of the total locations included in the analysis (24% in 2020), a true testament of the importance of personal contact in our culture. Less habitual activities, such as hair salons (*n* = 17) and gyms (*n* = 11), when the visit duration is more difficult for Google to capture, were also significantly represented in the data set. Since the median was used because visit duration data were not drawn from a normally distributed population, the standard error of the median could not be estimated by multiplying the standard error of the mean by a constant (1.2533). The width of the 95% confidence interval could represent a proxy for the significance level of the estimated parameter (median visit duration) since the width increases as the significance level decreases (30). Most of the median visit duration times by retail activity showed a narrow width of their respective 95% confidence intervals, confirming the accuracy of the effect size measure, the estimated parameter. Pubs and wine bars, hair salons and pizza restaurants showed a wider width of confidence intervals, possibly determined by an insufficient sampling or by the dual nature of their activity. For example, lunch in a pub or pizza restaurant takes significantly less time than dinner. This difference is smaller for fine dining restaurants, which always serve two or three-course meals. Similarly, a simple hair cut requires significantly less time than hair color, styling and salon treatments.

**Table 1:** Estimated parameter: median visit duration by retail activities.

**RETAIL ACTIVITIES (*n =* 808)**	**Median visit duration by retail activity in the metropolitan area of Genoa (Italy)**					
	**Sample (n)**	**MIN visit duration (minutes)**	**MAX visit duration (minutes)**	**Median visit duration (minutes)**	**95% Confidence Interval of the median**	
**Fine-dining restaurants**	48	15	90	60	60	60
**Pubs and wine bars**	32	15	90	30	25	45
**Hair salons**	17	15	60	30	25	45
**Shopping centers**	21	15	30	25	20	25
**Pizza restaurants**	78	5	90	20	15	45
**Gyms**	11	5	60	20	15	20
**Food supermarkets**	201	10	30	20	15	20
**Retail shops (non-food)**	91	10	45	20	20	20
**Fast-food restaurants**	31	10	45	15	15	20
**Coffee shops**	55	10	45	15	15	20
**Banks**	50	10	25	15	15	15
**Pharmacies**	81	10	20	15	15	15
**Post offices**	65	10	25	15	15	20
Gas stations ^*^	27	10	15	10	10	10

Both the non-parametric methods discussed in the “Methods” section allowed us to reject the null hypothesis that retail activity's median visit duration values were equal. The Kruskal-Wallis two-tailed test on all samples (K value: 2,245.76) rejected the null hypothesis since the computed *p-*value (<0.0001) was lower than the significance level (alpha = 0.05). Hence the samples did not come from the same distribution. Table 2 reports the pairwise significance of the Bonferroni-adjusted *P*-values, according to a degree of evidence: high (*p*-values < 0.0001); medium (0.0001 < *p*-vales < 0.01) and low (*p*-values > 0.01). 157 out of 169 (93%) of the pair-wise comparisons resulted highly or moderately significant. The Mood test on all samples (U statistic: 255.851; Critical value: 22.362; Degrees of Freedom: 13) confirmed that the computed *p*-value (<0.0001) was lower than the significance level alpha = 0.05. Hence the null hypothesis should be rejected, and the alternative hypothesis accepted: at least one of the medians was different from the other. The Mood's pair-wise comparisons confirmed the degrees of evidence obtained using the Kruskal-Wallis method. Both statistical tests are reported in full in the online Supplemental Material.

**Table 2:** Kruskal-Wallis two-tailed test on all samples of median visit duration time by retail activity: pair-wise significance of the Bonferroni-adjusted P-values according to a degree of evidence.

	**Fine dining** **restaurants**	**Pubs and** **wine bars**	**Hair** **salons**	**Shopping** **centers**	**Pizza** **restaurants**	**Gyms**	**Food** **supermarkets**	**Retail** **shops** **(non-food)**	**Fast-food** **restaurants**	**Coffee** **shops**	**Banks**	**Pharmacies**	**Post** **Offices**	**Gas** **stations**
Fine dining restaurants		Medium	High	High	High	High	High	High	High	High	High	High	High	High
Pubs and wine bars	Medium		Low	High	High	High	High	High	High	High	High	High	High	High
Hair salons	High	Low		High	High	High	High	High	High	High	High	High	High	High
Shopping centers	High	High	High		Low	Medium	High	High	High	High	High	High	High	High
Pizza restaurants	High	High	High	Low		Medium	High	High	High	High	High	High	High	High
Gyms	High	High	High	Medium	Medium		High	Medium	High	High	High	High	High	High
Food supermarkets	High	High	High	High	High	High		High	Medium	Low	High	High	Low	High
Retail shops (non- food)	High	High	High	High	High	Medium	High		Medium	High	High	High	High	High
Fast-food restaurants	High	High	High	High	High	High	Medium	Medium		Low	High	High	Medium	High
Coffee shops	High	High	High	High	High	High	Low	High	Low		High	High	Medium	High
Banks	High	High	High	High	High	High	High	High	High	High		High	Low	High
Pharmacies	High	High	High	High	High	High	High	High	High	High	High		High	Medium
Post Offices	High	High	High	High	High	High	Low	High	Medium	Low	Medium	High		High
Gas stations	High	High	High	High	High	High	High	High	High	High	High	Medium	High	

*Legend: High (p-values < 0.0001); Medium (0.0001 < p-values < 0.01) and Low (p-values > 0.01)*.

**Figure 2:**
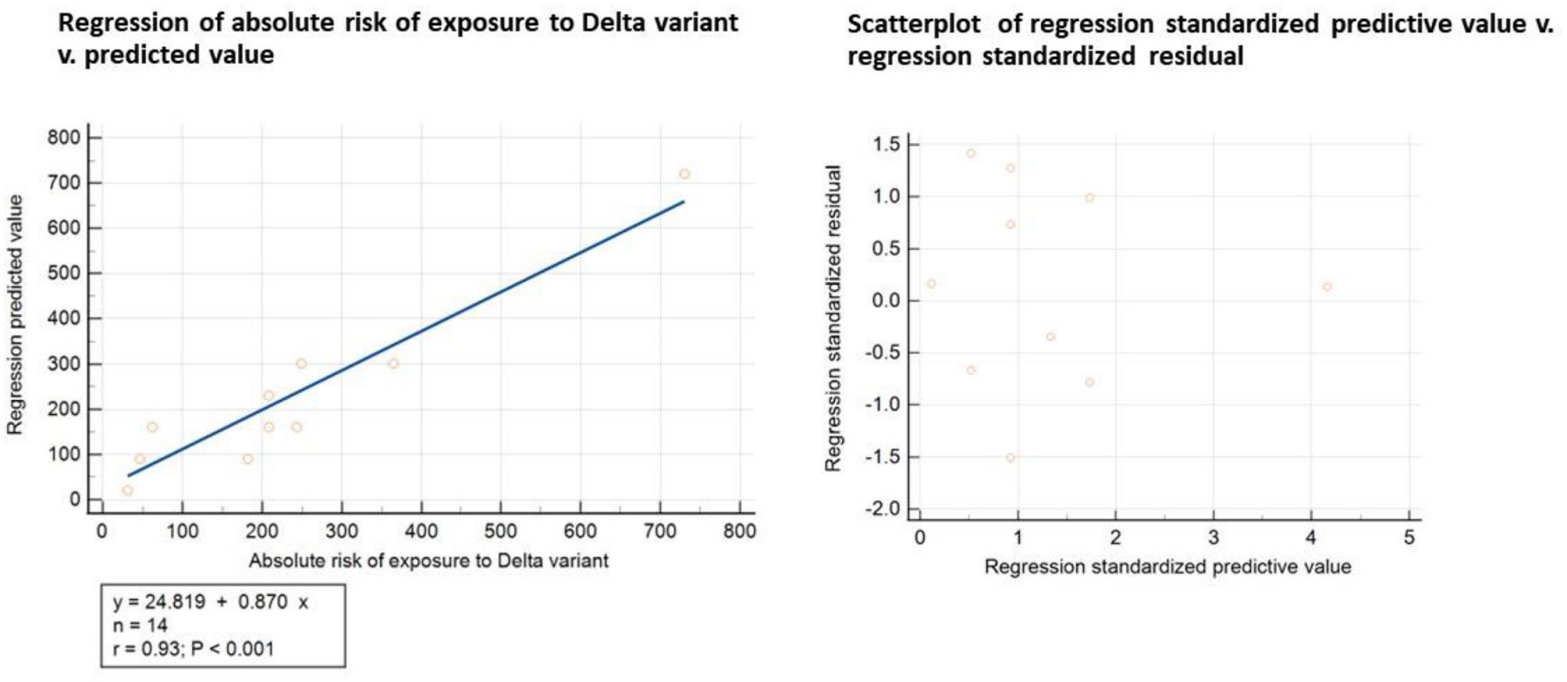
Statistical test of the accuracy of the risk of exposure model.

We then proceeded to test the accuracy of the risk of exposure model by regressing the median visit duration by store type against the predicted values of risk of exposure to the Delta variant. The Kolmogorov-Smirnov test with Lilliefors significance correction allowed to accept the normality of residuals (D = 0.2252; *p*-value = 0.0526). Figure 2 below reports the results of the least square regression of absolute v. predicted risk of exposure and the scatterplot of the regression standardized predictive value v. regression standardized residuals. The regression confirmed the model's predictive accuracy (r = 0.93, *p*-value < 0.001), and the scatterplot would exclude homoscedasticity. Regression standardized predictive values, and standardized residuals did not show any obvious pattern, with points equally distributed above and below zero on the X-axis and to the left and right of zero on the Y axis, except for a single outlier to the far right of the distribution. The outlier was represented by the absolute risk of exposure to the Delta variant associated with fine dining restaurants (standardized predictive value = 4.16): the relevance of this finding to public health policy will be better clarified in the following paragraphs.

The least-square regression data are reported in full in the online Supplementary Material.

Table 3 reports the risk of exposure to the Delta variant by retail activity based on the latest crowding norms and mobility data compared to the risk of exposure measured in December 2020, when the ancestral form of COVID-19 was prevalent.

**Table 3:** Absolute and relative risk of exposure to COVID-19 attributed to the Delta variant and the ancestral form of Covid-19 by retail activity.

**Retail activities**	**Median visit duration (minutes) Google Maps June 28, 2021**	**Median visit duration is a fraction of 15 seconds**	**Max crowding standard (people per square meter) Law 87, June 2021**	**Close contact area in square meters (CDC, October 2020)**	**Max number of people in the contact area**	**Absolute risk of exposure to DELTA variant**	**Absolute risk of exposure to ancestral form of Covid-19**	**Relative risk of exposure to DELTA variant**	**Relative risk of exposure to ancestral form of Covid-19**
	***a***	(a^*^60)/15	***c***	***d***	***c x d***	(a^*^60/15) x c x d	***December 2020 data***	***Gas stations = 1***	
**Fine-dining restaurants**	60	240	0.293	10.40	3.04	730.1	27.5	23.4	19.8
**Pubs and wine bars**	30	120	0.293	10.40	3.04	365.0	27.5	11.7	19.8
**Hair salons**	30	120	0.200	10.40	2.08	249.6	4.2	8.0	3.0
**Pizza restaurants**	20	80	0.293	10.40	3.04	243.4	27.5	7.8	19.8
**Shopping centers**	25	100	0.200	10.40	2.08	208.0	2.8	6.7	2.0
**Gyms**	20	80.00	0.250	10.40	2.60	208.0	9.1	6.7	6.5
**Fast-food restaurants**	15	60.00	0.293	10.40	3.04	182.5	11.4	5.9	8.2
**Coffee shops**	15	60.00	0.293	10.40	3.04	182.5	9.7	5.9	7.0
**Food supermarkets**	20	80.00	0.075	10.40	0.78	62.4	2.8	2.0	2.0
**Retail shops (non-food)**	20	80.00	0.075	10.40	0.78	62.4	2.8	2.0	2.0
**Banks**	15	60.00	0.075	10.40	0.78	46.8	2.1	1.5	1.5
**Pharmacies**	15	60.00	0.075	10.40	0.78	46.8	2.1	1.5	1.5
**Post offices**	15	60.00	0.075	10.40	0.78	46.8	2.1	1.5	1.5
Gas stations ^*^	10	40.00	0.075	10.40	0.78	31.2	1.4	1.0	1.0
***MEDIAN***						182.5	3.5	5.9	2.5

* *Max crowding standard refers to retail premises of the gas station (convenience store)*.

Both the Kruskal-Wallis two-tailed test and the Mood test confirmed the statistical significance of the differences in the median between the two observations. The Kruskal-Wallis two-tailed test on the two samples (K value: 20.382) rejected the null hypothesis since the computed p-value (<0.0001) was lower than the significance level (alpha = 0.05). Hence the samples did not come from the same distribution. The Mood test on the same samples (U statistic: 28.0; Critical value: 3.841; Degrees of Freedom: 1) confirmed that the computed *p*-value (<0.0001) was lower than the significance level alpha = 0.05. Hence the null hypothesis should be rejected, and the alternative hypothesis accepted: the median risk of exposure to the Delta variant and the ancestral form of COVID-19 were not equal.

Both statistical tests are reported in full in the online Supplemental Material.

**Figure 3:**
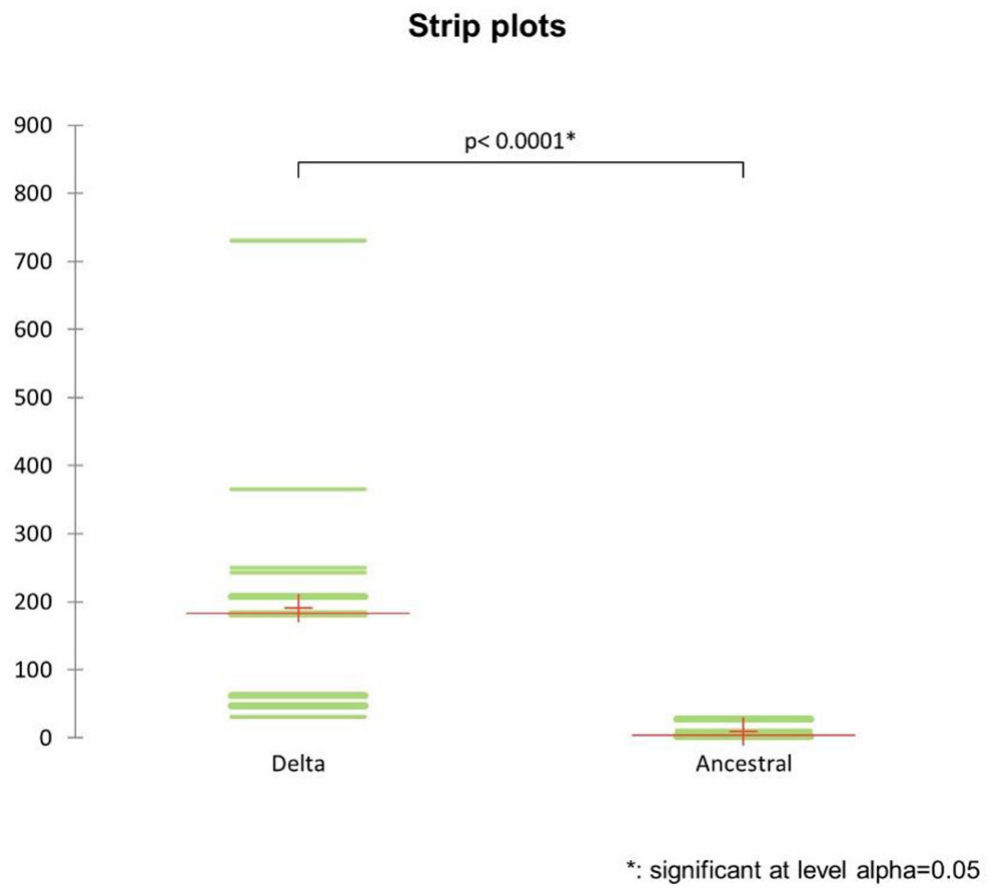
Strip plots of the absolute risk of exposure to the ancestral strain of Covid-19 and the Delta variant by retail activity.

The strip plots (Figure 3) of the absolute risk of exposure by retail activity showed a significant (*p*-value < 0.0001) variance of risk exposure to the Delta compared to the ancestral form of COVID-19, depending on our choice of activity and time spent on a retail premise. For example, the absolute risk of exposure ranged from a minimum of 31 when we stopped at a gas station to a record high of 730 if we decided to reward ourselves with a meal in a fine dining restaurant. In summary, the observed risk exposure to the Delta variant showed a three-tier risk structure for daily activities:

(1) HIGH RISK (risk of exposure above 300): fine-dining restaurants and pubs,
(2) MEDIUM RISK (risk of exposure from 100 to 300): fast-food restaurants, pizza restaurants, coffee shops, hair salons, shopping centers, and gyms;
(3) LOW RISK (risk of exposure below 100): retail shops (non-food), grocery supermarkets, pharmacies, banks, post offices and gas stations.

This new evidence should inform future public health policies concerning differential measures of social distancing, crowding and, ultimately, lockdown by retail activity.

**Figure 4:**
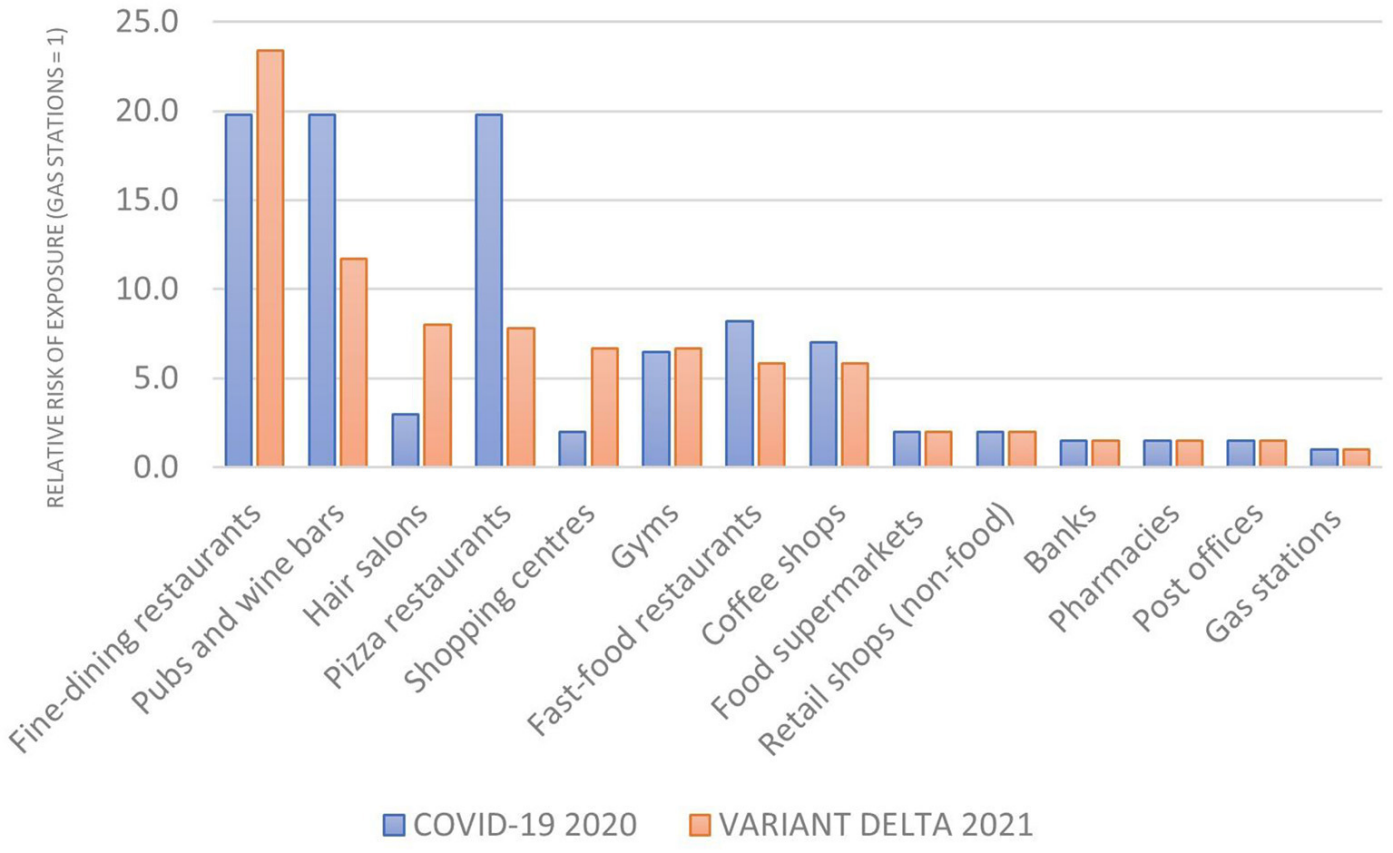
The relative risk of exposure (gas stations = 1).

Setting the lowest absolute value of the risk of exposure (gas stations) equal to 1, we obtained the relative risk of exposure by retail activity for both samples, as shown in Figure 4 below.

The comparative analysis of relative risk confirmed the three-tier risk structure observed for the absolute risk of exposure. Two retail activities reported a higher relative risk (fine dining restaurants and pub and wine bars) while the risk decreased in some premises (pubs, pizza restaurants, gyms and fast foods). For most activities, though, the relative risk of exposure remained unchanged, leading to a much smaller difference in median relative risk between 2021 (5.9) and 2020 (2.5) than the one observed for the absolute risk of exposure to the Delta variant v. the ancestral form of COVID-19.

The relative risk of exposure by retail activity in the metropolitan area of Genoa was measured at two distinct points in time:

December 2020 (when the ancestral form of Covid-19 was dominant) and June 2021 (when the Delta variant was prevalent).

Contrary to the absolute risk of exposure, both the Kruskal-Wallis two-tailed test and the Mood test agreed that the null hypothesis (the two medians were equal and came from the same population) could not be rejected. The Kruskal-Wallis two-tailed test on the two samples (K value: 0.119) could not reject the null hypothesis since the computed *p*-value (0.739) was higher than the significance level (alpha = 0.05). The Mood test on the same samples (U statistic: 0.571; Critical value: 3.841; Degrees of Freedom: 1) confirmed that the computed *p*-value (0.701) was lower than the significance level alpha = 0.05. Hence the null hypothesis should be rejected, and the alternative hypothesis accepted: the median relative risk of exposure to the Delta variant and the ancestral form of Covid-19 were equal. Both statistical tests are reported in full in the online Supplemental Material.

The data analysis and two non-parametric statistical tests confirmed that the absolute risk of exposure to the Delta variant significantly increased compared to its ancestral form due to its shorter time to close contact (competitive advantage). The median relative risk of exposure, though, did not significantly change. The two pieces of evidence satisfy the conditions of our working hypothesis: the Delta variant was not a “game changer” in the COVID-19 pandemic but rather a new round of the viral evolutionary game, a stable form of the “prisoner's dilemma”.

## Discussion

The analysis of median visit duration data by retail activity confirmed for the Delta variant what we already knew about COVID-19 on the potential risk of exposure when we go out. We spend up to one and a half hours sitting in restaurants, pubs and pizza places. Then, inevitably, remorse comes, and we exercise for 1 h at the gym. Even fast food can be not so fast: a hamburger gobbled up between two appointments takes about 10 min, but if we sit down immersed in our mobile phones, then the duration of the visit can almost quintuple. On the contrary, we are much more efficient in running our daily errands: it takes approximately 20 min to fill a cart at the supermarket, do essential shopping, or go in and out of a bank or post office. Visit duration times provide a clear indication that social activities should be, and are, a key priority for the containment of the diffusion of the Delta variant.

The comparative analysis between the risk of exposure to the ancestral form of COVID-19 estimated in December 2020, and the one attributed to the Delta variant measured approximately 6 months later provided insights relevant to public health policy. The first observation from the data reported in Table 3 is quite apparent: the median absolute risk of exposure to COVID-19 increased by sixty-fold in the latest semester. New data on visit duration and the relaxed crowding norm had a negligible impact on this dramatic change. Reducing close contact time from 15 min to 15 sec was the only determinant of the incremental, absolute risk of exposure.

The comparative epidemiological investigation of absolute and relative risk of exposure to COVID-19 in crowded metropolitan locations allowed us to accept our working hypothesis that the Delta variant is an evolutionary version of the game against COVID-19, not a game-changer. The shorter close contact time attributed to the Delta variant makes COVID-19 more transmissible, but it does not change the relative risk of exposure when we go out. Consequently, if we do not change our mitigation strategies (Tit-for-Tat), the relative risk of exposure to COVID-19 does not change, irrespective of the Delta variant. In this sense, COVID-19 has no incremental competitive advantage if the Delta variant completely replaces its ancestral form.

The best response strategy in an evolutionary stable game is to commit to the containment strategies already in place, and any competing alternative strategy should not replace them.

Consequently, public health decision-makers should not deviate from the chosen strategies to control the pandemic based on universal vaccination and social distancing (31).

It is the human containment strategy that selected the Delta variant. Viruses have a single, dominating strategic objective: to survive by infecting a host (32). Evolution proceeds by natural selection because the environment dictates which genetic variants favor contributing their genes to the next generation (33). In the game against COVID-19, our strategy to contain the pandemic determines the selection of a variant that is the “fittest” initially, but it will eventually lose out. If we change strategy, we offer the COVID-19 a unique opportunity to benefit from the new environment.

Our data on the risk of exposure to the Delta variant by retail premises confirm the game's evolution against COVID-19. The notion of crowding standards may have contributed to understating social activities' risk. When eating a meal or sipping a coffee, individuals necessarily put their masks down. Considering that face masks may significantly reduce exposure to the virus (34), the risk of exposure to COVID-19 for indoor social activities, such as exercising in a gym, enjoying a drink in a pub or a wine bar, and, most risky, consuming a meal in restaurants of any kind (including fast food), can be higher than expected.

The Delta variant does not seem to change the relative risk of exposure at a population level. Still, our current mitigation strategies might expose some individuals to a higher risk of COVID-19 infection.

Leisure activities are vital in the maintenance of both physical and mental wellbeing. Younger individuals privilege active leisure (social activities, exercising) while the aging population enjoys passive leisure (reading, watching television) (35). National vaccination plans identified elderly and vulnerable individuals as a priority target for immunization to prevent the vast majority of COVID-19 deaths well before herd immunity on the level of entire populations was achieved (36). Data indicates that vaccination may generate more neutralizing antibodies against Covid-19 variants than natural immunity (37).

Consequently, Millennials and Gen Z severely lagged in vaccinations. Vaccine uptake among adults between 18–39 years old has remained alarmingly low since all persons over the age of 16 have been eligible for COVID-19 immunization (38). The indications provided by our study are consistent with early epidemiological data on the “new wave” of Delta variant cases, showing that the majority of infections are among unvaccinated individuals below 40 years of age, who are less likely to fall seriously ill (39).

The empirical determination of the risk of exposure can inform national and local public health policies to contain the pandemic's diffusion. Compared to its ancestral form of COVID-19, the Delta variant puts time pressure on our strategy to contain the COVID-19 pandemic but is not a game-changer. Public health decision-makers should react to the new threat by continuing to play a Tit-for-Tat strategy. Stopping the spread at the source remains critical. Current measures to reduce transmission – including the vaccination of the younger strata of the population, wearing a mask in crowded premises and physical distancing – should continue to be our dominant strategy against the COVID-19 pandemic (40).

Looking at the global threat of the pandemic from a gaming perspective unlocks a further insight relevant to public health policy. The country's choices that contribute the least determine the outcome of all (41). Therefore, national strategies aimed to mitigate the effects of COVID-19 ought to be coordinated, as an outbreak anywhere in the world puts all other countries at risk. If one country relaxes its control measures and provokes an outbreak, all other countries will be negatively affected (42).

This research presents some limitations. First, the study is subject to a risk of selection bias in the population for whom data is available, limited to smartphone users who have turned on the Location History setting, which is off by default. It is a general limitation imposed by GPS mobility data (43). Spatially and temporally aggregated mobility data also do not capture differences in how individuals use their phones, making unfeasible any further cohort analysis (e.g., by users' age, gender or income). Secondly, the risk of exposure to Covid-19 and its variants can be influenced by many local risk factors, such as pollution (44), climate (45), seasonality (46), temperature (47), wind (48), relative humidity (49) demographics and local management of the pandemic (50). We tried to mitigate the impact of this wide variety of confounders by including in the study only residents of a single metropolitan area (Genoa, Italy) and by reducing the time allowed for data collection to one week, from 28/06/2021 to 02/07/2021.

In conclusion, our study shows that the Delta variant represents an evolution of the game against COVID-19, but it is not a game-changer. The best response to COVID-19 and its variants is to commit to our original Tit-for-Tat strategy based on population-wide vaccination and social distancing. Unilateral deviations from the dominant strategy could offer COVID-19 a fighting chance against humanity.

## Data Availability Statement

The original contributions presented in the study are included in the article/Supplementary Material, further inquiries can be directed to the corresponding author/s.

## Author Contributions

CO contributed to conception and design of the study, organized the database, performed the statistical analysis, and wrote the first draft of the manuscript. GF reviewed the first draft of the manuscript. All authors contributed to manuscript revision, read, and approved the submitted version.

## Conflict of Interest

The authors declare that the research was conducted in the absence of any commercial or financial relationships that could be construed as a potential conflict of interest.

## Publisher's Note

All claims expressed in this article are solely those of the authors and do not necessarily represent those of their affiliated organizations, or those of the publisher, the editors and the reviewers. Any product that may be evaluated in this article, or claim that may be made by its manufacturer, is not guaranteed or endorsed by the publisher.
