# Supplementary material for: From 15 Minutes to 15 Seconds: How the Delta Variant Changed the Risk of Exposure to COVID-19. A Comparative Epidemiological Investigation Using Community Mobility Data From the Metropolitan Area of Genoa, Italy
Source: Front Public Health. 2022 Jul 5;10:872698. doi: 10.3389/fpubh.2022.872698 (PMC9294394; doi:10.3389/fpubh.2022.872698)
Supplement: Supplementary file 1 [file Data_Sheet_1.DOCX]

Supplementary material

Table of Contents

[Mean visit duration in retail shopping premises: the Google Maps data 4](#_Toc105149829)

[Food supermarkets (n=201) 5](#_Toc105149830)

[Shopping centres (n=21) 9](#_Toc105149831)

[Coffee shops (n=55) 10](#_Toc105149832)

[Fast-food restaurants (n=31) 11](#_Toc105149833)

[Pubs and wine bars (n=22) 12](#_Toc105149834)

[Pizza restaurants (n=78) 13](#_Toc105149835)

[Fine dining restaurants (n=48) 15](#_Toc105149836)

[Retail shops non-food (n=91) 16](#_Toc105149837)

[Gyms (n=11) 18](#_Toc105149838)

[Hair saloons (n=14) 19](#_Toc105149839)

[Post offices (n=65) 19](#_Toc105149840)

[Banks (n=38) 21](#_Toc105149841)

[Gas stations (n=27) 22](#_Toc105149842)

[Pharmacies (n=35) 23](#_Toc105149843)

[Descriptive statistics 25](#_Toc105149844)

[Food supermarkets 25](#_Toc105149845)

[Shopping centres 26](#_Toc105149846)

[Coffee shops 27](#_Toc105149847)

[Fast-food restaurants 28](#_Toc105149848)

[Pubs and wine bars 29](#_Toc105149849)

[Pizza restaurants 30](#_Toc105149850)

[Fine dining restaurants 31](#_Toc105149851)

[Retail shops-non food 32](#_Toc105149852)

[Gyms 33](#_Toc105149853)

[Hair saloons 34](#_Toc105149854)

[Banks 35](#_Toc105149855)

[Post offices 36](#_Toc105149856)

[Pharmacies 37](#_Toc105149857)

[Gas stations 38](#_Toc105149858)

[Statistical analysis 39](#_Toc105149859)

[Median visit duration by retail activity 39](#_Toc105149860)

[Kruskall-Wallis test 40](#_Toc105149861)

[Mood test 43](#_Toc105149862)

[Model validity (least squares regression) 105](#_Toc105149863)

[Absolute risk of exposure 106](#_Toc105149864)

[Kruskal-Wallis test 106](#_Toc105149865)

[Mood test 110](#_Toc105149866)

[Relative risk of exposure 111](#_Toc105149867)

[Kruskal-Wallis test 111](#_Toc105149868)

[Mood Test 115](#_Toc105149869)

# Mean visit duration in retail shopping premises: the Google Maps data

Since October 2020, Google made visit duration time available on Google Maps (mobile version only). This data shows how much time customers typically spend in a specific store. Visit duration estimates are based on patterns of customer visits over the past several weeks.

Average visit duration is expressed in units of time (minutes).

Some retail activities show the visit duration as a range (e.g. 1.5 – 90 minutes). Since the aim of research was to estimate the incremental risk of exposure to the COVID-19 DELTA variant, which could bring the time to close contact from 15 minutes to just 15 seconds, when a range was reported we included in the analysis only the lower limit of visit duration.


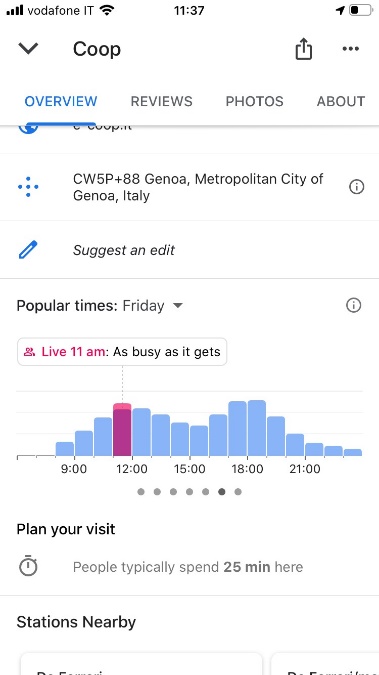

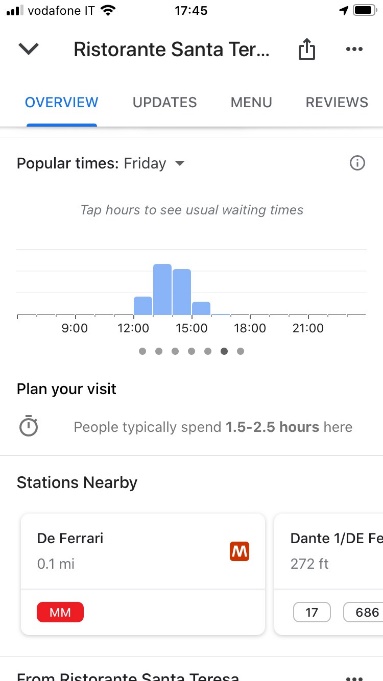


On June 28^th^, 2021, we manually collected visit duration data for 808 retail activities, banks and public offices located by Google Maps in the metropolitan area of Genoa, Italy.

The sample of visit data available included several retail activities:

Food supermarkets n=201

Shopping centres n= 21

Coffee shops n= 55

Fast-food restaurants n= 31

Pubs and wine bars n= 32

Pizza restaurants n= 78

Fine-dining restaurants n= 48

Retail shops (non-food) n= 91

Gyms n= 11

Hair salons n= 17

Banks n= 50

Pharmacies n= 81

Post offices n= 65

Gas stations n= 27

## Food supermarkets (n=201)

| **Store** | **Mean time in store (minutes)** |
| --- | --- |

| Coop Piccapietra | 25 |
| --- | --- |
| Coop Centro commerciale Europa | 20 |
| Coop Centro Commerciale Le Lampare | 20 |
| Coop Cso Gastaldi | 25 |
| Coop Centro commerciale Bisagno | 25 |
| Coop Centro commerciale Il Terminal | 25 |
| Coop Pzza Tre Ponti | 20 |
| Coop Salita F Da Paola | 20 |
| Coop Stazione Casella | 20 |
| Coop Via del Canto | 20 |
| Coop Via dei Mille | 20 |
| Coop via del Commercio | 20 |
| Coop Via delle Viazze | 10 |
| Coop Centro commerciale Il Mirto | 20 |
| Coop Via Franceschi | 25 |
| Coop Via Fumara | 20 |
| Coop Via Merano | 25 |
| Coop Via Prà | 20 |
| Coop Via Rivarolo | 20 |
| Coop Via Roana | 20 |
| Coop Via Romairone | 10 |
| Coop Via XX Settembre | 20 |
| Coop Viale Milite Ignoto | 25 |
| Conad Via Corsica | 25 |
| Conad Cso Sardegna | 15 |
| Conad Piazza Carloforte | 20 |
| Conad Via Anfossi | 25 |
| Conad Via Piccone | 20 |
| Conad Centro commerciale Fiumara | 10 |
| Conad Via Trossarelli | 15 |
| Conad Via Mascagni | 20 |
| Conad Via Linneo | 15 |
| Conad Via Murtola | 20 |
| Carrefour Campetto | 15 |
| Carrefour Cso Firenze | 15 |
| Carrefour Corso Sardegna | 15 |
| Carrefour Passo Centurione | 15 |
| Carrefour Piazza Giusti | 10 |
| Carrefour Pzza Marsala | 15 |
| Carrefour Pzza Merani | 15 |
| Carrefour Pzza Villa | 15 |
| Carrefour Pzzle Parenzo | 15 |
| Carrefour Salita S Maria | 15 |
| Carrefour Via Albaro | 15 |
| Carrefour Via Bari | 15 |
| Carrefour Via Bettini | 20 |
| Carrefour Via Bixio | 15 |
| Carrefour Via Bologna | 15 |
| Carrefour Via Bolzaneto | 15 |
| Carrefour Via Cairoli | 15 |
| Carrefour Via Canevari | 15 |
| Carrefour Via Cantore | 15 |
| Carrefour Via Casaregis | 15 |
| Carrefour Via Casaregis | 15 |
| Carrefour Via Cecchi | 20 |
| Carrefour Via Cesarea | 25 |
| Carrefour Via Chighizola | 15 |
| Carrefour Via Contubernio | 15 |
| Carrefour Via De Gasperi | 15 |
| Carrefour Via del Lagaccio | 15 |
| Carrefour Via delle Bernardine | 15 |
| Carrefour Via di Canneto | 15 |
| Carrefour Via Fareggiano | 15 |
| Carrefour Via Fiasella | 15 |
| Carrefour Via Fillak | 15 |
| Carrefour Via Filzi | 15 |
| Carrefour Via Gobetti | 15 |
| Carrefour Via isonzo | 15 |
| Carrefour Via Jori | 15 |
| Carrefour Via Martinetti | 15 |
| Carrefour Via Montaldo | 15 |
| Carrefour Via Montevideo | 10 |
| Carrefour Via Napoli | 15 |
| Carrefour Via Paleocapa | 15 |
| Carrefour Via Pisacane | 15 |
| Carrefour Via Quinto | 15 |
| Carrefour Via Rodi | 15 |
| Carrefour Via Rota | 20 |
| Carrefour Via S Agnese | 10 |
| Carrefour Via S De Rossi | 15 |
| Carrefour Via S Martino | 15 |
| Carrefour Corso Pio X | 15 |
| Carrefour Via S Vincenzo | 15 |
| Carrefour Via Tanini | 15 |
| Carrefour Via Torti | 20 |
| Carrefour Via Tortosa | 15 |
| Carrefour Vico Casana | 10 |
| PAM Via Manuzio | 25 |
| PAM Via Galata | 15 |
| PAM Via del Lagaccio | 20 |
| PAM Via Cantore | 15 |
| PAM Via Chiaravagna | 25 |
| PAM Via Fiasella | 15 |
| PAM Via Porta degli Archi | 20 |
| PAM Via Ruspoli | 15 |
| PAM Via Sestri | 15 |
| PAM Via Verità | 25 |
| Basko Piazza Sopranis | 25 |
| Basko Passo Antiochia | 15 |
| Basko Via Paggi | 25 |
| Basko Via Aurelia | 20 |
| Basko Via Barabino | 20 |
| Basko Via Barchetta | 25 |
| Basko Via Bertolotti | 30 |
| Basko Via Borzoli | 15 |
| Basko Via Cavallotti | 25 |
| Basko Via Cavour | 20 |
| Basko Via Centuriona | 25 |
| Basko Via Degola | 20 |
| Basko Via Emilia | 25 |
| Basko Via Langustena | 25 |
| Basko Via Martiri Libertà | 20 |
| Basko Via Molassana | 30 |
| Basko Via Paggi | 25 |
| Basko Via Posalunga | 15 |
| Basko Via Ricasoli | 15 |
| Basko Via Sturla | 20 |
| Basko Via Suardi | 20 |
| Basko Via Taggia | 20 |
| Basko Via Travi | 20 |
| Basko Via Vezzani | 25 |
| Basko Via Anfossi | 20 |
| Dipiù Via Alessi | 20 |
| Dipiù Via Sampierdarena | 15 |
| Dipiù Via Borgoratti | 20 |
| Dipiù Via Borzoli | 15 |
| Dipiù Via Murtola | 20 |
| Ekom Albaro | 20 |
| Ekom via Isonzo | 20 |
| Ekom Piazza Petrella | 20 |
| Ekom Via Archimede | 20 |
| Ekom Via Biga | 20 |
| Ekom Via Bobbio | 15 |
| Ekom Via Borgoratti | 15 |
| Ekom Via Bottego | 20 |
| Ekom Via Camozzini | 15 |
| Ekom Via Campomorone | 15 |
| Ekom Via Canevari | 15 |
| Ekom Via Cerruti | 20 |
| Ekom Via Chiaravagna | 15 |
| Ekom Via Colombo | 20 |
| Ekom Via Crimea | 20 |
| Ekom Via Custo | 20 |
| Ekom Via De Gaspari | 15 |
| Ekom Via del Lagaccio | 15 |
| Ekom Via della Castagna | 15 |
| Ekom Via Donghi | 15 |
| Ekom Via Ferrara | 25 |
| Ekom Via Gallino | 20 |
| Ekom Via Giovanni d'Acrì | 25 |
| Ekom Via Marussig | 20 |
| Ekom Via Molassana | 20 |
| Ekom Via Montebruno | 20 |
| Ekom Via Oberdan | 20 |
| Ekom Via Oriani | 20 |
| Ekom Via Passaggi | 20 |
| Ekom Via Petrella | 20 |
| Ekom Via Piacenza | 15 |
| Ekom Via Pinetti | 15 |
| Ekom Via Ponte Morosini | 15 |
| Ekom Via Prà | 25 |
| Ekom Via San Luca | 20 |
| Ekom Via S Martino | 15 |
| Ekom Via Torti | 15 |
| Ekom Via Turati | 15 |
| Ekom Via Vittorio Veneto | 15 |
| Doro Cso Armellini | 20 |
| Doro Passo Barsanti | 15 |
| Doro Via Melegari | 20 |
| Doro Via Carrea | 20 |
| Doro Via Chiodo | 15 |
| Doro Cso Martinetti | 15 |
| Doro Vle Franchini | 15 |
| Doro Via Bracelli | 15 |
| Doro Via del Commercio | 15 |
| Doro Via Lomellini | 15 |
| Doro Via Robino | 15 |
| iN's Mura di s Chiara | 20 |
| iN' Cso Perrone | 20 |
| iN's Cso de Stefanis | 20 |
| iN's Pzza Caroli | 15 |
| iN's Pzza Giusti | 20 |
| iN's Via Buranello | 20 |
| iN's Via Cantore | 15 |
| iN's Via Casaregis | 15 |
| iN's Via Centurione | 25 |
| iN's Via Donghi | 15 |
| iN's Via Fusinato | 20 |
| iN's Via Galata | 20 |
| iN's Via Giovanni Battista | 25 |
| iN's Via Jori | 15 |
| iN's Via Malfettani | 20 |
| iN's Via Mandoli | 15 |
| iN's Via Monticelli | 25 |
| iN's Via Paggi | 20 |
| iN's Via Parodi | 25 |
| iN's Via Piacenza | 15 |
| iN's Via S Martino | 20 |
| iN's Via Storace | 15 |
| iN's Via Merano | 15 |
| iN's Via Vecchia Filanda | 20 |

## Shopping centres (n=21)

| **Store** | **Mean time in store (minutes)** |
| --- | --- |

| Centro commerciale L'aquilone | 25 |
| --- | --- |
| Centro commerciale Europa | 30 |
| Il Terminal | 20 |
| Centro Commerciale Bisagno | 20 |
| Fiumara Shopping | 25 |
| Sotoripa | 20 |
| Shopping Center Molo 8.44 | 25 |
| Centro commerciale Le Serre | 25 |
| Centro commerciale Il Mirto | 25 |
| METRO | 20 |
| Ipercoop | 20 |
| COIN | 25 |
| Centro Commerciale Gabbiano | 25 |
| Centro Commerciale Le Lampare | 25 |
| I Leudi | 20 |
| Carrefour Market | 25 |
| Corte di Mare | 25 |
| La città sul Mare | 15 |
| La Riviera Shopville | 25 |
| Le Officine | 20 |
| Belforte | 25 |

## Coffee shops (n=55)

| **Store** | **Mean time in store (minutes)** |
| --- | --- |

| 23 Rosso | 15 |
| --- | --- |
| 7 Nasi | 15 |
| Bar Alfredo | 15 |
| Bar Boasi | 20 |
| Bar Bolla | 15 |
| Bar Centro | 25 |
| Bar Carioca | 15 |
| Bar Carletto 1960 | 15 |
| Bar Corallo | 15 |
| Bar Giardino | 25 |
| Bar Margherita | 10 |
| Bar Palli | 20 |
| Bar Paolo e Gianni | 15 |
| Bar Tagliafico | 15 |
| Bar Verdi | 25 |
| Basilico Caffè Albaro | 15 |
| Bisquit Bar | 15 |
| Caffè degli Specchi | 20 |
| Caffè Fogliotti | 15 |
| Cafè HB 1969 | 20 |
| Caffè Rolando | 20 |
| Caffetteria 68R | 10 |
| Caffetteria Carignano | 15 |
| Caffetteria Merano | 15 |
| Caffetteria Orefici | 25 |
| Caffetteria dell'Oratorio | 20 |
| Caffetteria Garrè | 15 |
| Caffetteria S Giorgio | 30 |
| Caffetteria S Giorgio Torti | 20 |
| Cafhein | 15 |
| Che Nervi | 15 |
| Corsi Caffetteria | 45 |
| De Lorenzi | 15 |
| De Stefanis | 20 |
| Don Cola | 25 |
| Don Paolo | 20 |
| Douce Patisserie Cafè | 20 |
| East River Cafè | 15 |
| Giumin | 15 |
| La Bottega del Caffè | 25 |
| La Piazza | 20 |
| La Superba | 25 |
| Mangini | 15 |
| Maca Caffè | 25 |
| Marina Bar | 15 |
| Mentelocale Bistrot | 20 |
| Merdiana caffè | 25 |
| P&O Bar | 15 |
| Quinto Caffè | 15 |
| Simo Caffè | 20 |
| Storico Lounge Cafe | 30 |
| Tazze Pazze | 30 |
| Tiffany Caffetteria | 20 |
| Tre Corone | 15 |
| Vintage Cafè 74 | 15 |

## Fast-food restaurants (n=31)

| **Store** | **Mean time in store (minutes)** |
| --- | --- |

| McDonald Via XX Settembre | 15 |
| --- | --- |
| Mc Donald Fiumara | 15 |
| McDonald Via di Sottoripa | 10 |
| Bowl! | 25 |
| Burger King Porto Antico | 20 |
| Burger King Via Mantovani | 15 |
| Burger Drive | 15 |
| Chicken & Chicken | 15 |
| Chicken & Chicken Via Contubernio | 15 |
| EatItaly | 30 |
| Fast Food Amico | 15 |
| Frigggitoria Carega | 20 |
| Gnam Gnam | 25 |
| King Kebab | 25 |
| Il Masetto | 20 |
| KFC Fiumara | 10 |
| La focacceria e dintorni | 15 |
| La Moucca | 20 |
| La Piadineria | 15 |
| Lo Stramburger | 20 |
| Marpione | 30 |
| Mc Donald Via Fiume | 15 |
| Old Wild West | 10 |
| Panino Marino | 25 |
| Poke and Bowl | 20 |
| Raviol House | 45 |
| Roaster Streetfood | 15 |
| Sbrano | 10 |
| Strakkino | 25 |
| Strike | 30 |
| Strike Albaro | 15 |

## Pubs and wine bars (n=22)

| **Store** | **Mean time in store (minutes)** |
| --- | --- |

| 23 Rosso | 15 |
| --- | --- |
| Ai Troeggi | 60 |
| Al Parador | 25 |
| Bar Bristol | 45 |
| Bar Degli Aperitivi | 15 |
| Bar Degli asinelli | 25 |
| Bar Festival | 25 |
| Bar Gemingway Pub | 25 |
| Bar Mini Mixing | 20 |
| Birreria Imbarco 1 | 60 |
| Cantiere Pub | 15 |
| Enoteca della Foce | 15 |
| La Coccagna | 90 |
| La Piazza | 20 |
| Il Baretto | 25 |
| Il Cantinone | 45 |
| Il Cavaturaccioli | 60 |
| Irish Pub | 30 |
| Kamun | 30 |
| La Goletta | 90 |
| La Lepre | 45 |
| La Pinta | 45 |
| Les Rouges | 45 |
| Manninvino | 15 |
| Molly Malone's | 60 |
| Negroneria Genovese | 45 |
| Otium | 15 |
| Paul Wine | 15 |
| Reset | 20 |
| Scurreria | 45 |
| Taverna Zaccaria | 60 |
| XO Food & Spirits | 45 |

## Pizza restaurants (n=78)

| **Store** | **Mean time in store (minutes)** |
| --- | --- |

| 23 Febbraio | 15 |
| --- | --- |
| Al Pisacane | 15 |
| Al Portico | 15 |
| Al Portico oregina | 15 |
| Al solito posto | 20 |
| Alice | 5 |
| Al Vulcano | 15 |
| Andrea Doria | 60 |
| Antica Vaccheria | 15 |
| Antico Borgo | 45 |
| Arte Pizza | 20 |
| Brera Express | 30 |
| City | 20 |
| Da Franz & Co. | 15 |
| Da Gibba | 90 |
| Dal Mister | 10 |
| Da Silva | 15 |
| Da Stefano | 60 |
| Del Ponte | 60 |
| Derby | 15 |
| Eataly | 30 |
| Eh..già | 20 |
| Europa Restaurant | 45 |
| Exultate | 60 |
| Ferrando | 10 |
| Focone | 90 |
| Fratelli La Bufala | 15 |
| Fuorigrotta | 15 |
| Garden | 60 |
| Gomez | 20 |
| Halloween | 90 |
| Kiss Me | 45 |
| I Tre Merli | 90 |
| Il Baluardo | 60 |
| Il Mortaio | 15 |
| Il Sette | 60 |
| Il Torchio | 20 |
| Il Vicolo | 60 |
| I Sassi | 45 |
| La Funicolare | 10 |
| La Legnaia | 15 |
| La Locanda del Molo | 45 |
| Le Mani in Pasta | 90 |
| La Ola | 60 |
| La Pizza di Egizio | 15 |
| La Terrazza | 60 |
| Le Tre Caravelle | 60 |
| Lo Scugnizzo | 15 |
| Massarjia | 30 |
| MoroMare | 20 |
| MoroMare Nervi | 60 |
| New O Sole Mio | 15 |
| Osteria della Piazza | 45 |
| Pazzi per la Pizza | 10 |
| Pestello d'oro | 60 |
| Pianeta Pizza | 20 |
| Pinseria Romana | 15 |
| Piuma | 60 |
| Pizza Express | 15 |
| Pizzeria Napoletana da Paolo | 10 |
| Pizzeria Stadio | 10 |
| Pulcinella | 15 |
| Punta Vagno | 15 |
| Pluto 2 | 15 |
| Rosso Pomodoro | 90 |
| Sapori di Sori | 60 |
| Sbrano | 10 |
| Sereno 1950 | 45 |
| Soho | 60 |
| Sole Luna | 60 |
| Sosta Obbligata | 20 |
| Strakkino | 25 |
| Tiflis | 60 |
| Totò e Peppino | 15 |
| Ulu Pizzeria Kebab | 15 |
| Va Pensiero | 60 |
| Voglie di Pizza | 20 |
| Zena Zuena | 30 |

## Fine dining restaurants (n=48)

| **Store** | **Mean time in store (minutes)** |
| --- | --- |

| 2f | 60 |
| --- | --- |
| 5 Maggio | 60 |
| Al Marmo | 25 |
| Antica Osteria Ravecca | 60 |
| Cavour Modo 21 | 45 |
| Cottura 9' | 60 |
| Da Gibba | 90 |
| Europa | 45 |
| I Tre merli | 90 |
| Il Baluardo | 60 |
| Il Gelsomino | 90 |
| Il Sette Genova | 60 |
| I Tre Merli | 90 |
| Ittiturismo | 60 |
| Kapperi | 60 |
| Kiss me | 45 |
| La Ola | 60 |
| La Terrazza | 60 |
| Le Cantine | 90 |
| Le Cicale in città | 15 |
| Le Cicale in trattoria | 60 |
| Le Colonne | 60 |
| Mangiabuono | 60 |
| Opera | 30 |
| Osteria Vico Palla | 90 |
| Ostetrattoria | 90 |
| Paradiso | 25 |
| Rio Samba | 60 |
| Rosmarino | 60 |
| RossoPomodoro | 90 |
| Santa Teresa | 45 |
| Settepolpette | 60 |
| Soho | 60 |
| Strakkino | 25 |
| The Cook | 60 |
| Toe Drue | 15 |
| Trattoria Acciughetta | 90 |
| Trattoria Archivolto | 90 |
| Trattoria Arvigo | 60 |
| Trattoria de Ruscin | 15 |
| Trattoria delle Grazie | 60 |
| Trattoria Archivolto | 90 |
| Trattoria Osvaldo | 90 |
| Trattoria Ugo | 60 |
| Vapensiero | 60 |
| Vegia Zena | 60 |
| Vivarelli | 90 |
| Zena Zuena | 30 |

## Retail shops non-food (n=91)

| **Store** | **Mean time in store (minutes)** |
| --- | --- |

| \| 420 shop \| 15 \|  \| \| --- \| --- \| --- \| \| Acqua & Sapone \| 20 \|  \| \| Antony Morato \| 30 \|  \| \| Arredo 3 store \| 15 \|  \| \| Asta del Mobile \| 20 \|  \| \| Bag store \| 10 \|  \| \| Bata \| 25 \|  \| \| Benetton \| 25 \|  \| \| Best Shopp \| 25 \|  \| \| Bikers store \| 25 \|  \| \| Bimbostore \| 30 \|  \| \| Bricoman \| 20 \|  \| \| Calzedonia \| 20 \|  \| \| C&C Genova \| 15 \|  \| \| Comics corner \| 30 \|  \| \| Corderia Nazionale \| 15 \|  \| \| Dainese \| 25 \|  \| \| Decathlon \| 25 \|  \| \| Deichman \| 25 \|  \| \| DM Italia \| 20 \|  \| \| Dmail \| 20 \|  \| \| Dungeon \| 25 \|  \| \| Expert \| 20 \|  \| \| Feltrinelli book & Music \| 45 \|  \| \| Fisherlandia \| 15 \|  \| \| Flying Tiger Copenhagen \| 10 \|  \| \| Full House \| 20 \|  \| \| Game People \| 20 \|  \| \| Geox XX Settembre 1 \| 25 \|  \| \| Geox XX Setembre 2 \| 25 \|  \| \| Geox Fiumara \| 10 \|  \| \| Globo \| 25 \|  \| \| Guess \| 10 \|  \| \| H&M \| 30 \|  \| \| Il libraccio \| 20 \|  \| \| Il libraccio Via Cairoli \| 30 \|  \| \| Intrend \| 25 \|  \| \| Kasanova \| 20 \|  \| \| La Befana \| 20 \|  \| \| La formica econegozio \| 15 \|  \| \| Lego store \| 20 \|  \| \| Leroy Merlin \| 25 \|  \| \| Louis Vuitton \| 15 \|  \| \| Lush \| 15 \|  \| \| Maison du Monde \| 30 \|  \| \| MD Market \| 25 \|  \| \| MediaWorld \| 15 \|  \| \| Mi store \| 10 \|  \| \| Moisman Sport \| 25 \|  \| \| Mondadori bookstore \| 30 \|  \| \| Mondadori Sestri \| 30 \|  \| \| Motivi \| 30 \|  \| \| Nespresso boutique \| 15 \|  \| \| Peter Tea House \| 20 \|  \| \| Piana utensili \| 15 \|  \| \| Pitta Rosso \| 10 \|  \| \| Primadonna Fiumara \| 10 \|  \| \| Primadonna XX Settembre \| 25 \|  \| \| Primo \| 20 \|  \| \| Promoclub \| 20 \|  \| \| OVS Fiumara \| 10 \|  \| \| OVS Brigata Liguria \| 25 \|  \| \| OVS Via Sestri \| 25 \|  \| \| Red House musical instruments \| 20 \|  \| \| Robe di Kappa \| 20 \|  \| \| Romanengo \| 15 \|  \| \| SAGE sanitaria \| 20 \|  \| \| Salewa \| 20 \|  \| \| Sampdoria point \| 20 \|  \| \| Scarpe & scarpe \| 15 \|  \| \| Sephora \| 10 \|  \| \| Stylecar \| 15 \|  \| \| Stradivarius via XX Settembre \| 25 \|  \| \| Stradivarius Fiumara \| 10 \|  \| \| Stroili \| 25 \|  \| \| Subdued \| 25 \|  \| \| Supershop Brignole \| 20 \|  \| \| Supershop Bisagno \| 10 \|  \| \| Swatch store \| 15 \|  \| \| Tessil Moda \| 15 \|  \| \| Thun shop via XX Zsettembre \| 20 \|  \| \| Thun shop Fiumara \| 10 \|  \| \| Tigotà \| 20 \|  \| \| Tipinifini \| 20 \|  \| \| Toys centre \| 25 \|  \| \| Triumph lingerie \| 25 \|  \| \| Unieuro \| 20 \|  \| \| Valigeria Sanson \| 20 \|  \| \| Wheelup \| 25 \|  \| \| Wurth \| 15 \|  \| \| Zuicki \| 10 \|  \| |  |  |
| --- | --- | --- | --- | --- | --- | --- | --- | --- | --- | --- | --- | --- | --- | --- | --- | --- | --- | --- | --- | --- | --- | --- | --- | --- | --- | --- | --- | --- | --- | --- | --- | --- | --- | --- | --- | --- | --- | --- | --- | --- | --- | --- | --- | --- | --- | --- | --- | --- | --- | --- | --- | --- | --- | --- | --- | --- | --- | --- | --- | --- | --- | --- | --- | --- | --- | --- | --- | --- | --- | --- | --- | --- | --- | --- | --- | --- | --- | --- | --- | --- | --- | --- | --- | --- | --- | --- | --- | --- | --- | --- | --- | --- | --- | --- | --- | --- | --- | --- | --- | --- | --- | --- | --- | --- | --- | --- | --- | --- | --- | --- | --- | --- | --- | --- | --- | --- | --- | --- | --- | --- | --- | --- | --- | --- | --- | --- | --- | --- | --- | --- | --- | --- | --- | --- | --- | --- | --- | --- | --- | --- | --- | --- | --- | --- | --- | --- | --- | --- | --- | --- | --- | --- | --- | --- | --- | --- | --- | --- | --- | --- | --- | --- | --- | --- | --- | --- | --- | --- | --- | --- | --- | --- | --- | --- | --- | --- | --- | --- | --- | --- | --- | --- | --- | --- | --- | --- | --- | --- | --- | --- | --- | --- | --- | --- | --- | --- | --- | --- | --- | --- | --- | --- | --- | --- | --- | --- | --- | --- | --- | --- | --- | --- | --- | --- | --- | --- | --- | --- | --- | --- | --- | --- | --- | --- | --- | --- | --- | --- | --- | --- | --- | --- | --- | --- | --- | --- | --- | --- | --- | --- | --- | --- | --- | --- | --- | --- | --- | --- | --- | --- | --- | --- | --- | --- | --- | --- | --- | --- | --- | --- | --- | --- | --- | --- | --- | --- | --- | --- | --- | --- | --- | --- | --- | --- | --- |

## Gyms (n=11)

| **Store** | **Mean time in store (minutes)** |
| --- | --- |

| Well & Fit | 5 |
| --- | --- |
| Gli Sportivi | 20 |
| FitActive Campi | 20 |
| FitActive Piazza Dante | 60 |
| Foltzer Fitness | 25 |
| Gruppo sportivo Aragno | 20 |
| AFD FPX | 15 |
| HiFit Club | 15 |
| Impianto sportivo Sciorba | 15 |
| San Fruttuoso Pool | 15 |
| ASD Sturla sports | 20 |

## Hair saloons (n=14)

| **Store** | **Mean time in store (minutes)** |
| --- | --- |

| Aesse Parrucchieri | 15 |
| --- | --- |
| RR Parrucchieri | 60 |
| Barberia Monsieur | 30 |
| Elisa Look and Style | 25 |
| Emme Diffusion | 30 |
| Equipe Giorgio Lab Haidresser | 60 |
| Equipe Guastamacchia | 45 |
| Russo Barber Chic | 45 |
| Hair Loris | 30 |
| Hairteca | 15 |
| Immagine | 60 |
| IG La Barberia | 45 |
| La Barberia Italiana | 45 |
| The Yambi Barber Shop | 45 |
| Nadege Hairdresser | 15 |
| Geko | 30 |
| Elisa Look & Style | 25 |

## Post offices (n=65)

| **Store** | **Mean time in store (minutes)** |
| --- | --- |

| Poste Cso Europa 1068 | 15 |
| --- | --- |
| Poste Cso Europa 546 | 15 |
| Poste Corso Firenze | 10 |
| Poste Cso Marconi | 15 |
| Poste Corso Martinetti | 20 |
| Poste Cso Sardegna 2 | 15 |
| Poste Cso Sardegna 2 | 20 |
| Poste Piazza Cavour | 15 |
| Poste Piazza Gaggero | 25 |
| Poste Piazza Monastero | 15 |
| Poste Piazza Rovere | 25 |
| Poste Piazzale Marassi | 25 |
| Poste Piazzale Sigelli | 15 |
| Poste Piazzale Rizzolio | 25 |
| Poste Via Airaghi | 20 |
| Poste Via Assarotti | 20 |
| Poste Via Blelè | 15 |
| Poste Via Borzoli | 15 |
| Poste Via Caldesi | 20 |
| Poste Via Canevari | 15 |
| Poste Via Cantore | 20 |
| Poste Via Carbone | 20 |
| Poste Via Catalani | 25 |
| Poste Viale Cembrano | 15 |
| Poste Via Colombo | 20 |
| Poste Via da Pozzo | 20 |
| Poste Via dal Canto | 20 |
| Poste Via Dante | 15 |
| Poste Via Donghi | 15 |
| Poste Via Fereggiano | 20 |
| Poste Via Franchini | 20 |
| Poste Via Francia | 15 |
| Poste Via Giannelli | 15 |
| Poste Via Granello | 20 |
| Poste Via Ilva | 15 |
| Poste Via Langustena | 15 |
| Poste Corso Martinetti | 20 |
| Poste Via Martiri della Libertà | 20 |
| Poste Via Molassana | 15 |
| Poste Via Multedo | 15 |
| Poste Via Napoli | 25 |
| Poste Via Olivieri | 15 |
| Poste Via Orsini | 20 |
| Poste Via Pastorino | 20 |
| Poste Via Piacenza | 15 |
| Poste Via Piccone | 25 |
| Poste Via Pisa | 15 |
| Poste Via Poirè | 10 |
| Poste Via Pozzo | 20 |
| Poste Via Re di Puglia | 20 |
| Poste Via Robino | 20 |
| Poste Via Roggerone | 20 |
| Poste Via Spalato | 15 |
| Poste Via S Franceco de Paola | 25 |
| Poste Via San Fruttuoso | 15 |
| Poste Via San Quirico | 15 |
| Poste Via San Romolo | 15 |
| Poste Via Sauli | 15 |
| Poste Via Schiaffino | 25 |
| Poste Via Spalato | 15 |
| Poste Via Struppa | 15 |
| Poste Via Terpi | 15 |
| Poste Via Toscanelli | 25 |
| Poste Via Ulanowski | 20 |
| Poste Via Valle Chiara | 15 |

## Banks (n=38)

| **Store** | **Mean time in store (minutes)** |
| --- | --- |

| Banca CARIGE Via Corsica | 15 |
| --- | --- |
| Banca di Asti Via Brigata Liguria | 15 |
| Banca di Sondrio Pzza Tomasseo | 20 |
| Banca di Sondrio Via XXV Aprile | 20 |
| Banca Generali Private | 15 |
| Banca Italia Via Dante | 20 |
| Banca Passadore | 15 |
| Banca Passaore Via Sestri | 15 |
| Banca Sella Via Fieschi | 20 |
| Banco di Sardegna Pzza Fontane Marose | 20 |
| BNL Corso Torino | 15 |
| BNL Largo Lanfranco | 15 |
| BNL Via Bolzaneto | 15 |
| BNL Via Brigata Liguria | 15 |
| BNL Via Caprera | 10 |
| BNL Via Catalani | 15 |
| BNL Via Gherzi | 20 |
| BNL Via Jori | 15 |
| BNL Piazza Montano | 15 |
| BNL Via Martiri della Libertà | 15 |
| BNL Via Murcarolo | 15 |
| BNL Via Tortosa | 15 |
| BNL Via Torti | 15 |
| BPER Banca Viale Brigate Bisagno | 20 |
| Carige al Monte di Pietà | 25 |
| Carige Piazza delle Americhe | 15 |
| Carige Via Avio | 10 |
| Carige Via Cesarea | 25 |
| Carige Via Cassa di Risparmio | 15 |
| Carige Via Torti | 10 |
| CheBanca! Via Roma | 15 |
| Compass Via Brigata Bisagno | 25 |
| Credem Piazza Dante | 20 |
| Credem Piazza Martinez | 15 |
| ING Pzza Dante | 15 |
| Intesa Cso Sardegna | 15 |
| Intesa Pzza Fontane Marose | 15 |
| Intesa Via Fieschi | 15 |
| Intesa Via Righetti | 10 |
| Intesa Via Rolando | 15 |
| Intesa Via Sestri | 15 |
| Intesa Via Timavo | 15 |
| MPS Corso Sardegna | 10 |
| Unicredit Via Cornigliano | 15 |
| Unicredit Via Fiasella | 15 |
| Unicredit Via Garibaldi | 20 |
| Unicredit Via Gastaldi | 10 |
| Unicredit Via Piacenza | 15 |
| Unicredit Via Sestri | 10 |
| Unicredit Via Vittorio veneto | 15 |

## Gas stations (n=27)

| **Store** | **Mean time in the store (minutes)** |
| --- | --- |

| Automobilgas Via Borzoli | 10 |
| --- | --- |
| Automobilgas Via Trensaco | 10 |
| Barisone Via Ronchi | 15 |
| ENI Via Archimede | 10 |
| ENI Via Reta | 10 |
| Esso Cso Europa | 10 |
| Esso Piazza Dinegro | 10 |
| Esso Piazza Manin | 10 |
| Esso Molassana | 10 |
| Esso Via Lungobisagno | 10 |
| Esso Via Pegli | 10 |
| Europam Cso Magenta | 10 |
| Europam Via Borzoli | 10 |
| Europam Via Bianchi | 10 |
| IP Via Cantore | 10 |
| IP Via Diaz | 10 |
| IP Via Ferri | 10 |
| Q8 Cso Europa | 10 |
| Q8 S.Ilario | 15 |
| Q8 Lanterna Est | 10 |
| Q8 Via Adamoli | 10 |
| Q8 Via dei Mille | 10 |
| Q8 Via Multedo | 10 |
| Q8 Via Vasco de Gama | 10 |
| Stazione di Servizio Via Lemerle | 10 |
| Tamoil Via Canepari | 10 |
| Tamoil Corso Europa | 10 |

## Pharmacies (n=35)

| **Store** | **Mean time in the store (minutes)** |
| --- | --- |

| Farmacia Alvigini | 15 |
| --- | --- |
| Farmacia Amoretti | 15 |
| Farmacia Assarotti | 10 |
| Farmacia Barabino | 15 |
| Farmacia Bassano | 20 |
| Farmacia Baudoin | 15 |
| Farmacia Bonanni | 15 |
| Farmacia Burlando | 20 |
| Farmacia Campart | 10 |
| Farmacia Canevari | 15 |
| Farnacia Cantore | 15 |
| Farmacia Cappuccini | 15 |
| Farmacia Carlevaro | 15 |
| Farmacia Arte Farmaceutica | 15 |
| Farmacia Castelletto | 15 |
| Farmacia Centrale | 15 |
| Farmacia Comunale | 15 |
| Farmacia Comunale 2 | 15 |
| Farmacia Comunale Molassana | 15 |
| Farmacia Comunale Burlando | 15 |
| Farmacia de Ferrari | 15 |
| Farmacia Nazionale | 15 |
| Farmacia Del Chiappazzo | 15 |
| Farmacia Oregina | 15 |
| Farmacia dell'Aquila | 15 |
| Farmacia Nuovo Lido di Albaro | 10 |
| Farmacia Dell'Oro | 10 |
| Farmacia Europa | 10 |
| Farmacia Ghersi | 10 |
| Farmacia Genovese | 15 |
| Farmacia Imperiale | 15 |
| Farmacia Ligure | 15 |
| Farnacia Lloyds | 15 |
| Farmacia Lloyds albaro | 15 |
| Farmacia Martinelli | 15 |
| Farmacia Moderna | 15 |
| Farmacia Modigliani | 15 |
| Farmacia Monticelli | 15 |
| Farmacia Multedo | 10 |
| Farmacia della Nunziata | 15 |
| Farmacia Nizza | 15 |
| Farmacia Nostra Signora del monte | 15 |
| Farmacia Palmaro | 15 |
| Farmacia Pedrini | 15 |
| Farmacia Pescetto | 15 |
| Farmacia Popolare | 15 |
| Farmacia Ponte Monumentale | 15 |
| Farmacia Quarto dei Mille | 15 |
| Farmacia Ribaldone | 10 |
| Farmacia Santa Rosa | 15 |
| Farmacia san Giacomo | 15 |
| Farmacia San Raffaele | 10 |
| Farmacia San Rocco | 15 |
| Farmacia San Sebastiano | 15 |
| Farmacia Sarzano | 15 |
| Farmacia Scanavino | 10 |
| Farmacia Sanitas | 15 |
| Farmacia Sangiorgi | 15 |
| Faarmacia Terrile | 15 |
| Framacia San Pietro | 15 |
| Farmacia Sturla | 10 |
| Farmacia Iachetti | 10 |
| Farmacia Giusto | 15 |
| Farmacia Massa | 15 |
| Farmacia Ponte Pila | 15 |
| Farmacia Lloyds Castelletto | 15 |
| Farmacia Majonchi | 10 |
| Farmacia Certosa | 15 |
| Farmacia Saltarelli | 15 |
| Farmacia Orientale | 15 |
| Farmacia Comunale Sestri Ponente | 15 |
| Farmacia Vesuvio | 15 |
| Farmacia Dagnino | 15 |
| Farmacia San Bernardo | 15 |
| Farmacia Gallo | 15 |
| Farmacia Moderna | 15 |
| Farmacia Dapelo | 15 |
| Farmacia Piaggio | 15 |
| Farmacia San Giorgio | 10 |
| Farmacia Lagaccio | 15 |
| Farmacia Centrale | 15 |

# Descriptive statistics

Statistical software: MedCalc

## Food supermarkets

|  |  |
| --- | --- |
| **Sample size** | 201 |
| **Lowest value** | [10](cmd:SHOWXMINMAX?13) |
| **Highest value** | [30](cmd:SHOWXMINMAX?104) |
| **Arithmetic mean** | 18.1592 |
| **95% CI for the Arithmetic mean** | 17.5969 to 18.7215 |
| **Geometric mean** | 17.7204 |
| **95% CI for the Geometric mean** | 17.1807 to 18.2770 |
| **Harmonic mean** | 17.2878 |
| **95% CI for the Harmonic mean** | 16.7592 to 17.8509 |
| **Median** | 20 |
| **95% CI for the median** | 15.0000 to 20.0000 |
| **Variance** | 16.3445 |
| **Standard deviation** | 4.0428 |
| **Relative standard deviation** | 0.2226 (22.26%) |
| **Standard error of the mean** | 0.2852 |
| **Coefficient of Skewness** | 0.4820 (P=0.0063) |
| **Coefficient of Kurtosis** | -0.2699 (P=0.4330) |
| **Chi-squared test** | reject Normality (P<0.0001) |
| **for Normal distribution** | (Chi-squared=752.541 DF=14) |
| **10% Trimmed mean (n=181)** | 18.0939 |
| **95% CI of Trimmed mean** | 17.5277 to 18.6602 |

## Shopping centres

| **Sample size** | 21 |
| --- | --- |
| **Lowest value** | [15](cmd:SHOWXMINMAX?18) |
| **Highest value** | [30](cmd:SHOWXMINMAX?2) |
| **Arithmetic mean** | 23.0952 |
| **95% CI for the Arithmetic mean** | 21.5725 to 24.6180 |
| **Geometric mean** | 22.8477 |
| **95% CI for the Geometric mean** | 21.3037 to 24.5036 |
| **Harmonic mean** | 22.5806 |
| **95% CI for the Harmonic mean** | 21.0018 to 24.4161 |
| **Median** | 25 |
| **95% CI for the median** | 20.0000 to 25.0000 |
| **Variance** | 11.1905 |
| **Standard deviation** | 3.3452 |
| **Relative standard deviation** | 0.1448 (14.48%) |
| **Standard error of the mean** | 0.73 |
| **Coefficient of Skewness** | -0.4812 (P=0.3170) |
| **Coefficient of Kurtosis** | 0.4653 (P=0.4839) |
| **Chi-squared test** | reject Normality (P=0.0003) |
| **for Normal distribution** | (Chi-squared=16.472 DF=2) |
| **10% Trimmed mean (n=19)** | 23.1579 |
| **95% CI of Trimmed mean** | 21.8938 to 24.4220 |

## Coffee shops

| **Sample size** | 55 |
| --- | --- |
| **Lowest value** | [10](cmd:SHOWXMINMAX?11) |
| **Highest value** | [45](cmd:SHOWXMINMAX?32) |
| **Arithmetic mean** | 19.0909 |
| **95% CI for the Arithmetic mean** | 17.4645 to 20.7173 |
| **Geometric mean** | 18.3184 |
| **95% CI for the Geometric mean** | 16.9748 to 19.7683 |
| **Harmonic mean** | 17.6534 |
| **95% CI for the Harmonic mean** | 16.4674 to 19.0234 |
| **Median** | 15 |
| **95% CI for the median** | 15.0000 to 20.0000 |
| **Variance** | 36.1953 |
| **Standard deviation** | 6.0163 |
| **Relative standard deviation** | 0.3151 (31.51%) |
| **Standard error of the mean** | 0.8112 |
| **Coefficient of Skewness** | 1.7537 (P<0.0001) |
| **Coefficient of Kurtosis** | 5.1197 (P=0.0003) |
| **Chi-squared test** | reject Normality (P<0.0001) |
| **for Normal distribution** | (Chi-squared=133.654 DF=8) |
| **10% Trimmed mean (n=51)** | 18.7255 |
| **95% CI of Trimmed mean** | 17.3080 to 20.1430 |

## Fast-food restaurants

| **Sample size** | 31 |
| --- | --- |
| **Lowest value** | [10](cmd:SHOWXMINMAX?3) |
| **Highest value** | [45](cmd:SHOWXMINMAX?26) |
| **Arithmetic mean** | 19.3548 |
| **95% CI for the Arithmetic mean** | 16.5637 to 22.1460 |
| **Geometric mean** | 18.1074 |
| **95% CI for the Geometric mean** | 15.8353 to 20.7055 |
| **Harmonic mean** | 17.0122 |
| **95% CI for the Harmonic mean** | 15.0532 to 19.5573 |
| **Median** | 15 |
| **95% CI for the median** | 15.0000 to 20.0000 |
| **Variance** | 57.9032 |
| **Standard deviation** | 7.6094 |
| **Relative standard deviation** | 0.3932 (39.32%) |
| **Standard error of the mean** | 1.3667 |
| **Coefficient of Skewness** | 1.3845 (P=0.0030) |
| **Coefficient of Kurtosis** | 2.8825 (P=0.0180) |
| **Chi-squared test** | reject Normality (P<0.0001) |
| **for Normal distribution** | (Chi-squared=23.937 DF=3) |
| **10% Trimmed mean (n=29)** | 18.7931 |
| **95% CI of Trimmed mean** | 16.3180 to 21.2682 |

## Pubs and wine bars

| **Sample size** | 32 |
| --- | --- |
| **Lowest value** | [15](cmd:SHOWXMINMAX?1) |
| **Highest value** | [90](cmd:SHOWXMINMAX?13) |
| **Arithmetic mean** | 37.1875 |
| **95% CI for the Arithmetic mean** | 29.5676 to 44.8074 |
| **Geometric mean** | 31.8617 |
| **95% CI for the Geometric mean** | 25.9519 to 39.1173 |
| **Harmonic mean** | 27.4286 |
| **95% CI for the Harmonic mean** | 22.9791 to 34.0149 |
| **Median** | 30 |
| **95% CI for the median** | 24.9880 to 45.0000 |
| **Variance** | 446.6734 |
| **Standard deviation** | 21.1346 |
| **Relative standard deviation** | 0.5683 (56.83%) |
| **Standard error of the mean** | 3.7361 |
| **Coefficient of Skewness** | 0.9342 (P=0.0289) |
| **Coefficient of Kurtosis** | 0.4182 (P=0.4652) |
| **Chi-squared test** | reject Normality (P<0.0001) |
| **for Normal distribution** | (Chi-squared=33.654 DF=4) |
| **10% Trimmed mean (n=30)** | 36.1667 |
| **95% CI of Trimmed mean** | 28.0073 to 44.3261 |

## Pizza restaurants

| **Sample size** | 78 |
| --- | --- |
| **Lowest value** | [5](cmd:SHOWXMINMAX?6) |
| **Highest value** | [90](cmd:SHOWXMINMAX?15) |
| **Arithmetic mean** | 35.3846 |
| **95% CI for the Arithmetic mean** | 29.7766 to 40.9927 |
| **Geometric mean** | 27.4033 |
| **95% CI for the Geometric mean** | 23.2337 to 32.3212 |
| **Harmonic mean** | 21.3959 |
| **95% CI for the Harmonic mean** | 18.4803 to 25.4038 |
| **Median** | 20 |
| **95% CI for the median** | 15.0000 to 45.0000 |
| **Variance** | 618.6813 |
| **Standard deviation** | 24.8733 |
| **Relative standard deviation** | 0.7029 (70.29%) |
| **Standard error of the mean** | 2.8163 |
| **Coefficient of Skewness** | 0.7722 (P=0.0071) |
| **Coefficient of Kurtosis** | -0.5997 (P=0.1713) |
| **Chi-squared test** | reject Normality (P<0.0001) |
| **for Normal distribution** | (Chi-squared=127.601 DF=11) |
| **10% Trimmed mean (n=72)** | 34.2361 |
| **95% CI of Trimmed mean** | 28.1671 to 40.3052 |

## Fine dining restaurants

| **Sample size** | 48 |
| --- | --- |
| **Lowest value** | [15](cmd:SHOWXMINMAX?20) |
| **Highest value** | [90](cmd:SHOWXMINMAX?7) |
| **Arithmetic mean** | 60.625 |
| **95% CI for the Arithmetic mean** | 54.0455 to 67.2045 |
| **Geometric mean** | 55.1437 |
| **95% CI for the Geometric mean** | 47.8706 to 63.5219 |
| **Harmonic mean** | 47.8405 |
| **95% CI for the Harmonic mean** | 40.0020 to 59.4998 |
| **Median** | 60 |
| **95% CI for the median** | 60.0000 to 60.0000 |
| **Variance** | 513.4309 |
| **Standard deviation** | 22.659 |
| **Relative standard deviation** | 0.3738 (37.38%) |
| **Standard error of the mean** | 3.2705 |
| **Coefficient of Skewness** | -0.3125 (P=0.3453) |
| **Coefficient of Kurtosis** | -0.4952 (P=0.4581) |
| **Chi-squared test** | reject Normality (P<0.0001) |
| **for Normal distribution** | (Chi-squared=75.783 DF=7) |
| **10% Trimmed mean (n=44)** | 61.3636 |
| **95% CI of Trimmed mean** | 54.1614 to 68.5659 |

## Retail shops-non food

| **Sample size** | 91 |
| --- | --- |
| **Lowest value** | [10](cmd:SHOWXMINMAX?6) |
| **Highest value** | [45](cmd:SHOWXMINMAX?24) |
| **Arithmetic mean** | 20.2198 |
| **95% CI for the Arithmetic mean** | 18.8584 to 21.5811 |
| **Geometric mean** | 19.1219 |
| **95% CI for the Geometric mean** | 17.7908 to 20.5525 |
| **Harmonic mean** | 17.9645 |
| **95% CI for the Harmonic mean** | 16.6515 to 19.5021 |
| **Median** | 20 |
| **95% CI for the median** | 20.0000 to 20.0000 |
| **Variance** | 42.7289 |
| **Standard deviation** | 6.5367 |
| **Relative standard deviation** | 0.3233 (32.33%) |
| **Standard error of the mean** | 0.6852 |
| **Coefficient of Skewness** | 0.4053 (P=0.1069) |
| **Coefficient of Kurtosis** | 0.9721 (P=0.0904) |
| **Chi-squared test** | reject Normality (P<0.0001) |
| **for Normal distribution** | (Chi-squared=127.709 DF=10) |
| **10% Trimmed mean (n=83)** | 20.0602 |

## Gyms

| **Sample size** | 11 |
| --- | --- |
| **Lowest value** | [5](cmd:SHOWXMINMAX?1) |
| **Highest value** | [60](cmd:SHOWXMINMAX?4) |
| **Arithmetic mean** | 20.9091 |
| **95% CI for the Arithmetic mean** | 11.5497 to 30.2685 |
| **Geometric mean** | 17.9081 |
| **95% CI for the Geometric mean** | 12.1211 to 26.4580 |
| **Harmonic mean** | 15.2074 |
| **95% CI for the Harmonic mean** | 10.2740 to 29.2553 |
| **Median** | 20 |
| **95% CI for the median** | 15.0000 to 20.8985 |
| **Variance** | 194.0909 |
| **Standard deviation** | 13.9317 |
| **Relative standard deviation** | 0.6663 (66.63%) |
| **Standard error of the mean** | 4.2006 |
| **Coefficient of Skewness** | 2.4837 (P=0.0008) |
| **Coefficient of Kurtosis** | 7.5343 (P=0.0016) |
| **Chi-squared test** | cannot be assessed |
| **for Normal distribution** |  |

## Hair saloons

| **Sample size** | 17 |
| --- | --- |
| **Lowest value** | [15](cmd:SHOWXMINMAX?1) |
| **Highest value** | [60](cmd:SHOWXMINMAX?2) |
| **Arithmetic mean** | 36.4706 |
| **95% CI for the Arithmetic mean** | 28.5072 to 44.4340 |
| **Geometric mean** | 33.0824 |
| **95% CI for the Geometric mean** | 25.9151 to 42.2320 |
| **Harmonic mean** | 29.5938 |
| **95% CI for the Harmonic mean** | 23.3862 to 40.2878 |
| **Median** | 30 |
| **95% CI for the median** | 25.0806 to 45.0000 |
| **Variance** | 239.8897 |
| **Standard deviation** | 15.4884 |
| **Relative standard deviation** | 0.4247 (42.47%) |
| **Standard error of the mean** | 3.7565 |
| **Coefficient of Skewness** | 0.1599 (P=0.7581) |
| **Coefficient of Kurtosis** | -1.0852 (P=0.2023) |
| **Chi-squared test** | reject Normality (P=0.0258) |
| **for Normal distribution** | (Chi-squared=4.972 DF=1) |

## Banks

| **Sample size** | 50 |
| --- | --- |
| **Lowest value** | [10](cmd:SHOWXMINMAX?15) |
| **Highest value** | [25](cmd:SHOWXMINMAX?25) |
| **Arithmetic mean** | 15.8 |
| **95% CI for the Arithmetic mean** | 14.7507 to 16.8493 |
| **Geometric mean** | 15.39 |
| **95% CI for the Geometric mean** | 14.4065 to 16.4406 |
| **Harmonic mean** | 14.985 |
| **95% CI for the Harmonic mean** | 14.0338 to 16.0745 |
| **Median** | 15 |
| **95% CI for the median** | 15.0000 to 15.0000 |
| **Variance** | 13.6327 |
| **Standard deviation** | 3.6922 |
| **Relative standard deviation** | 0.2337 (23.37%) |
| **Standard error of the mean** | 0.5222 |
| **Coefficient of Skewness** | 0.6847 (P=0.0451) |
| **Coefficient of Kurtosis** | 0.8072 (P=0.2107) |
| **Chi-squared test** | reject Normality (P<0.0001) |
| **for Normal distribution** | (Chi-squared=96.008 DF=6) |
| **10% Trimmed mean (n=46)** | 15.6522 |
| **95% CI of Trimmed mean** | 14.5080 to 16.7963 |

## Post offices

| **Sample size** | 65 |
| --- | --- |
| **Lowest value** | [10](cmd:SHOWXMINMAX?3) |
| **Highest value** | [25](cmd:SHOWXMINMAX?9) |
| **Arithmetic mean** | 18.0769 |
| **95% CI for the Arithmetic mean** | 17.1050 to 19.0488 |
| **Geometric mean** | 17.6641 |
| **95% CI for the Geometric mean** | 16.7381 to 18.6412 |
| **Harmonic mean** | 17.2566 |
| **95% CI for the Harmonic mean** | 16.3604 to 18.2568 |
| **Median** | 15 |
| **95% CI for the median** | 15.0000 to 20.0000 |
| **Variance** | 15.3846 |
| **Standard deviation** | 3.9223 |
| **Relative standard deviation** | 0.2170 (21.70%) |
| **Standard error of the mean** | 0.4865 |
| **Coefficient of Skewness** | 0.4055 (P=0.1655) |
| **Coefficient of Kurtosis** | -0.6399 (P=0.1771) |
| **Chi-squared test** | reject Normality (P<0.0001) |
| **for Normal distribution** | (Chi-squared=177.159 DF=10) |
| **10% Trimmed mean (n=59)** | 18.0508 |
| **95% CI of Trimmed mean** | 17.0407 to 19.0610 |

## Pharmacies

| **Sample size** | 81 |
| --- | --- |
| **Lowest value** | [10](cmd:SHOWXMINMAX?3) |
| **Highest value** | [20](cmd:SHOWXMINMAX?5) |
| **Arithmetic mean** | 14.2593 |
| **95% CI for the Arithmetic mean** | 13.7931 to 14.7254 |
| **Geometric mean** | 14.0845 |
| **95% CI for the Geometric mean** | 13.5835 to 14.6039 |
| **Harmonic mean** | 13.8857 |
| **95% CI for the Harmonic mean** | 13.3476 to 14.4690 |
| **Median** | 15 |
| **95% CI for the median** | 15.0000 to 15.0000 |
| **Variance** | 4.4444 |
| **Standard deviation** | 2.1082 |
| **Relative standard deviation** | 0.1478 (14.78%) |
| **Standard error of the mean** | 0.2342 |
| **Coefficient of Skewness** | -0.9260 (P=0.0015) |
| **Coefficient of Kurtosis** | 1.5220 (P=0.0304) |
| **Chi-squared test** | reject Normality (P<0.0001) |
| **for Normal distribution** | (Chi-squared=579.303 DF=10) |
| **10% Trimmed mean (n=73)** | 14.3151 |
| **95% CI of Trimmed mean** | 13.8472 to 14.7829 |

## Gas stations

| **Sample size** | 27 |
| --- | --- |
| **Lowest value** | [10](cmd:SHOWXMINMAX?1) |
| **Highest value** | [15](cmd:SHOWXMINMAX?3) |
| **Arithmetic mean** | 10.3704 |
| **95% CI for the Arithmetic mean** | 9.8425 to 10.8982 |
| **Geometric mean** | 10.3049 |
| **95% CI for the Geometric mean** | 9.8731 to 10.7556 |
| **Harmonic mean** | 10.2532 |
| **95% CI for the Harmonic mean** | 9.8961 to 10.6370 |
| **Median** | 10 |
| **95% CI for the median** | 10.0000 to 10.0000 |
| **Variance** | 1.7806 |
| **Standard deviation** | 1.3344 |
| **Relative standard deviation** | 0.1287 (12.87%) |
| **Standard error of the mean** | 0.2568 |
| **Coefficient of Skewness** | 3.4472 (P<0.0001) |
| **Coefficient of Kurtosis** | 10.6704 (P=0.0001) |
| **Chi-squared test** | reject Normality (P<0.0001) |
| **for Normal distribution** | (Chi-squared=72.693 DF=1) |
| **10% Trimmed mean (n=25)** | 10.2 |
| **95% CI of Trimmed mean** | 9.6267 to 10.7733 |

# Statistical analysis

## Median visit duration by retail activity

| Kruskall-Wallis test Summary statistics: |  |  |  |  |  |  |  |  |  |
| --- | --- | --- | --- | --- | --- | --- | --- | --- | --- |
|  |  |  |  |  |  |  |  |  |  |
| Variable | Observations | Obs. with missing data | Obs. without missing data | Minimum | Maximum | Mean | Std. deviation |  |  |
| Fine dining restaurants | 201 | 0 | 201 | 15.000 | 90.000 | 60.625 | 10.984 |  |  |
| Pubs and wine bars | 201 | 0 | 201 | 15.000 | 90.000 | 37.188 | 8.321 |  |  |
| Hair salons | 201 | 0 | 201 | 15.000 | 60.000 | 36.471 | 4.381 |  |  |
| Shopping centres | 201 | 0 | 201 | 15.000 | 30.000 | 23.095 | 1.058 |  |  |
| Pizza restaurants | 201 | 0 | 201 | 5.000 | 90.000 | 35.385 | 15.433 |  |  |
| Gyms | 201 | 0 | 201 | 5.000 | 60.000 | 20.909 | 3.115 |  |  |
| Food supermarkets | 201 | 0 | 201 | 10.000 | 30.000 | 18.159 | 4.043 |  |  |
| Retail shops (non food) | 201 | 0 | 201 | 10.000 | 45.000 | 20.220 | 4.385 |  |  |
| Fast-food restaurants | 201 | 0 | 201 | 10.000 | 45.000 | 19.355 | 2.947 |  |  |
| Coffee shops | 201 | 0 | 201 | 10.000 | 45.000 | 19.091 | 3.126 |  |  |
| Banks | 201 | 0 | 201 | 10.000 | 25.000 | 15.800 | 1.828 |  |  |
| Pharmacies | 201 | 0 | 201 | 10.000 | 20.000 | 14.259 | 1.333 |  |  |
| Post Offices | 201 | 0 | 201 | 10.000 | 25.000 | 18.077 | 2.219 |  |  |
| Gas stations | 201 | 0 | 201 | 10.000 | 15.000 | 10.370 | 0.481 |  |  |
|  |  |  |  |  |  |  |  |  |  |
|  |  |  |  |  |  |  |  |  |  |
| Kruskal-Wallis test / Two-tailed test: | | |  |  |  |  |  |  |  |
|  |  |  |  |  |  |  |  |  |  |
| K | 2245.376 |  |  |  |  |  |  |  |  |
| p-value (one-tailed) | **<0.0001** |  |  |  |  |  |  |  |  |
| alpha | 0.050 |  |  |  |  |  |  |  |  |
| The p-value has been computed using 10000 Monte Carlo simulations. Time elapsed: 2s. | | | | | | | |  |  |
| 99% confidence interval on the p-value: | | |  |  |  |  |  |  |  |
| ] 0.000, | 0.000 [ |  |  |  |  |  |  |  |  |
|  |  |  |  |  |  |  |  |  |  |
| Test interpretation: |  |  |  |  |  |  |  |  |  |
| H0: The samples come from the same population. | | | |  |  |  |  |  |  |
| Ha: The samples do not come from the same population. | | | | |  |  |  |  |  |
| As the computed p-value is lower than the significance level alpha=0.05, one should reject the null hypothesis H0, and accept the alternative hypothesis Ha. | | | | | | | | |  |
|  |  |  |  |  |  |  |  |  |  |
|  |  |  |  |  |  |  |  |  |  |
|  |  |  |  |  |  |  |  |  |  |
| Ties have been detected in the data and the appropriate corrections have been applied. | | | | | | | | |  |

### Mood test

| Summary statistics: | |  |  |  |  |  |  |  |  |
| --- | --- | --- | --- | --- | --- | --- | --- | --- | --- |
|  |  |  |  |  |  |  |  |  |  |
| Variable | Observations | Obs. with missing data | Obs. without missing data | Minimum | Maximum | Mean | Std. deviation |  |  |
| Fine dining restaurants | 201 | 153 | 48 | 15.000 | 90.000 | 60.625 | 22.659 |  |  |
| Pubs and wine bars | 201 | 169 | 32 | 15.000 | 90.000 | 37.188 | 21.135 |  |  |
| Hair salons | 201 | 184 | 17 | 15.000 | 60.000 | 36.471 | 15.488 |  |  |
| Shopping centres | 201 | 180 | 21 | 15.000 | 30.000 | 23.095 | 3.345 |  |  |
| Pizza restaurants | 201 | 123 | 78 | 5.000 | 90.000 | 35.385 | 24.873 |  |  |
| Gyms | 201 | 190 | 11 | 5.000 | 60.000 | 20.909 | 13.932 |  |  |
| Food supermarkets | 201 | 0 | 201 | 10.000 | 30.000 | 18.159 | 4.043 |  |  |
| Retail shops (non food) | 201 | 110 | 91 | 10.000 | 45.000 | 20.220 | 6.537 |  |  |
| Fast-food restaurants | 201 | 170 | 31 | 10.000 | 45.000 | 19.355 | 7.609 |  |  |
| Coffee shops | 201 | 146 | 55 | 10.000 | 45.000 | 19.091 | 6.016 |  |  |
| Banks | 201 | 151 | 50 | 10.000 | 25.000 | 15.800 | 3.692 |  |  |
| Pharmacies | 201 | 120 | 81 | 10.000 | 20.000 | 14.259 | 2.108 |  |  |
| Post Offices | 201 | 136 | 65 | 10.000 | 25.000 | 18.077 | 3.922 |  |  |
| Gas stations | 201 | 174 | 27 | 10.000 | 15.000 | 10.370 | 1.334 |  |  |
|  |  |  |  |  |  |  |  |  |  |
|  |  |  |  |  |  |  |  |  |  |
| **Mood test:** | |  |  |  |  |  |  |  |  |
|  |  |  |  |  |  |  |  |  |  |
| U | 255.851 |  |  |  |  |  |  |  |  |
| Critical value | 22.362 |  |  |  |  |  |  |  |  |
| DF | 13.000 |  |  |  |  |  |  |  |  |
| p-value | < 0.0001 |  |  |  |  |  |  |  |  |
| alpha | 0.05 |  |  |  |  |  |  |  |  |
| The p-value has been computed using 10000 Monte Carlo simulations. Time elapsed: 6s. | | | | | | | | |  |
|  |  |  |  |  |  |  |  |  |  |
| Test interpretation: | |  |  |  |  |  |  |  |  |
| H0: The medians are all equal. | | |  |  |  |  |  |  |  |
| Ha: At least one of the medians is different from another. | | | | | |  |  |  |  |
| As the computed p-value is lower than the significance level alpha=0.05, one should reject the null hypothesis H0, and accept the alternative hypothesis Ha. | | | | | | | | |  |
|  |  |  |  |  |  |  |  |  |  |
|  |  |  |  |  |  |  |  |  |  |
|  |  |  |  |  |  |  |  |  |  |
| 95% confidence interval on the p-value: | | | |  |  |  |  |  |  |
| ] 0.000, | 0.000 [ |  |  |  |  |  |  |  |  |
|  |  |  |  |  |  |  |  |  |  |
| **Multiple pairwise comparisons:** | | | |  |  |  |  |  |  |
|  |  |  |  |  |  |  |  |  |  |
| Mood test (Fine dining restaurants\|Pubs and wine bars): | | | | | |  |  |  |  |
|  |  |  |  |  |  |  |  |  |  |
| U | 4.188 |  |  |  |  |  |  |  |  |
| Critical value | 3.841 |  |  |  |  |  |  |  |  |
| DF | 1.000 |  |  |  |  |  |  |  |  |
| p-value | 0.035 |  |  |  |  |  |  |  |  |
| alpha | 0.05 |  |  |  |  |  |  |  |  |
| The p-value has been computed using 10000 Monte Carlo simulations. Time elapsed: 6s. | | | | | | | | |  |
|  |  |  |  |  |  |  |  |  |  |
| Test interpretation: | |  |  |  |  |  |  |  |  |
| H0: The medians of Fine dining restaurants and Pubs and wine bars are equal. | | | | | | | |  |  |
| Ha: Medians of Fine dining restaurants and Pubs and wine bars are not equal | | | | | | | |  |  |
| As the computed p-value is lower than the significance level alpha=0.05, one should reject the null hypothesis H0, and accept the alternative hypothesis Ha. | | | | | | | | |  |
|  |  |  |  |  |  |  |  |  |  |
|  |  |  |  |  |  |  |  |  |  |
|  |  |  |  |  |  |  |  |  |  |
| 95% confidence interval on the p-value: | | | |  |  |  |  |  |  |
| ] 0.031, | 0.039 [ |  |  |  |  |  |  |  |  |
|  |  |  |  |  |  |  |  |  |  |
| Mood test (Fine dining restaurants\|Hair salons): | | | | |  |  |  |  |  |
|  |  |  |  |  |  |  |  |  |  |
| U | 4.187 |  |  |  |  |  |  |  |  |
| Critical value | 3.841 |  |  |  |  |  |  |  |  |
| DF | 1.000 |  |  |  |  |  |  |  |  |
| p-value | 0.029 |  |  |  |  |  |  |  |  |
| alpha | 0.05 |  |  |  |  |  |  |  |  |
| The p-value has been computed using 10000 Monte Carlo simulations. Time elapsed: 6s. | | | | | | | | |  |
|  |  |  |  |  |  |  |  |  |  |
| Test interpretation: | |  |  |  |  |  |  |  |  |
| H0: The medians of Fine dining restaurants and Hair salons are equal. | | | | | | |  |  |  |
| Ha: Medians of Fine dining restaurants and Hair salons are not equal | | | | | | |  |  |  |
| As the computed p-value is lower than the significance level alpha=0.05, one should reject the null hypothesis H0, and accept the alternative hypothesis Ha. | | | | | | | | |  |
|  |  |  |  |  |  |  |  |  |  |
|  |  |  |  |  |  |  |  |  |  |
|  |  |  |  |  |  |  |  |  |  |
| 95% confidence interval on the p-value: | | | |  |  |  |  |  |  |
| ] 0.026, | 0.032 [ |  |  |  |  |  |  |  |  |
|  |  |  |  |  |  |  |  |  |  |
| Mood test (Fine dining restaurants\|Shopping centres): | | | | | |  |  |  |  |
|  |  |  |  |  |  |  |  |  |  |
| U | 5.349 |  |  |  |  |  |  |  |  |
| Critical value | 3.841 |  |  |  |  |  |  |  |  |
| DF | 1.000 |  |  |  |  |  |  |  |  |
| p-value | 0.014 |  |  |  |  |  |  |  |  |
| alpha | 0.05 |  |  |  |  |  |  |  |  |
| The p-value has been computed using 10000 Monte Carlo simulations. Time elapsed: 6s. | | | | | | | | |  |
|  |  |  |  |  |  |  |  |  |  |
| Test interpretation: | |  |  |  |  |  |  |  |  |
| H0: The medians of Fine dining restaurants and Shopping centres are equal. | | | | | | | |  |  |
| Ha: Medians of Fine dining restaurants and Shopping centres are not equal | | | | | | | |  |  |
| As the computed p-value is lower than the significance level alpha=0.05, one should reject the null hypothesis H0, and accept the alternative hypothesis Ha. | | | | | | | | |  |
|  |  |  |  |  |  |  |  |  |  |
|  |  |  |  |  |  |  |  |  |  |
|  |  |  |  |  |  |  |  |  |  |
| 95% confidence interval on the p-value: | | | |  |  |  |  |  |  |
| ] 0.012, | 0.017 [ |  |  |  |  |  |  |  |  |
|  |  |  |  |  |  |  |  |  |  |
| Mood test (Fine dining restaurants\|Pizza restaurants): | | | | | |  |  |  |  |
|  |  |  |  |  |  |  |  |  |  |
| U | 20.260 |  |  |  |  |  |  |  |  |
| Critical value | 3.841 |  |  |  |  |  |  |  |  |
| DF | 1.000 |  |  |  |  |  |  |  |  |
| p-value | < 0.0001 |  |  |  |  |  |  |  |  |
| alpha | 0.05 |  |  |  |  |  |  |  |  |
| The p-value has been computed using 10000 Monte Carlo simulations. Time elapsed: 6s. | | | | | | | | |  |
|  |  |  |  |  |  |  |  |  |  |
| Test interpretation: | |  |  |  |  |  |  |  |  |
| H0: The medians of Fine dining restaurants and Pizza restaurants are equal. | | | | | | | |  |  |
| Ha: Medians of Fine dining restaurants and Pizza restaurants are not equal | | | | | | | |  |  |
| As the computed p-value is lower than the significance level alpha=0.05, one should reject the null hypothesis H0, and accept the alternative hypothesis Ha. | | | | | | | | |  |
|  |  |  |  |  |  |  |  |  |  |
|  |  |  |  |  |  |  |  |  |  |
|  |  |  |  |  |  |  |  |  |  |
| 95% confidence interval on the p-value: | | | |  |  |  |  |  |  |
| ] 0.000, | 0.000 [ |  |  |  |  |  |  |  |  |
|  |  |  |  |  |  |  |  |  |  |
| Mood test (Fine dining restaurants\|Gyms): | | | | |  |  |  |  |  |
|  |  |  |  |  |  |  |  |  |  |
| U | 2.407 |  |  |  |  |  |  |  |  |
| Critical value | 3.841 |  |  |  |  |  |  |  |  |
| DF | 1.000 |  |  |  |  |  |  |  |  |
| p-value | 0.112 |  |  |  |  |  |  |  |  |
| alpha | 0.05 |  |  |  |  |  |  |  |  |
| The p-value has been computed using 10000 Monte Carlo simulations. Time elapsed: 6s. | | | | | | | | |  |
|  |  |  |  |  |  |  |  |  |  |
| Test interpretation: | |  |  |  |  |  |  |  |  |
| H0: The medians of Fine dining restaurants and Gyms are equal. | | | | | | |  |  |  |
| Ha: Medians of Fine dining restaurants and Gyms are not equal | | | | | | |  |  |  |
| As the computed p-value is greater than the significance level alpha=0.05, one cannot reject the null hypothesis H0. | | | | | | | | |  |
|  |  |  |  |  |  |  |  |  |  |
|  |  |  |  |  |  |  |  |  |  |
|  |  |  |  |  |  |  |  |  |  |
| 95% confidence interval on the p-value: | | | |  |  |  |  |  |  |
| ] 0.106, | 0.118 [ |  |  |  |  |  |  |  |  |
|  |  |  |  |  |  |  |  |  |  |
| Mood test (Fine dining restaurants\|Food supermarkets): | | | | | |  |  |  |  |
|  |  |  |  |  |  |  |  |  |  |
| U | 108.436 |  |  |  |  |  |  |  |  |
| Critical value | 3.841 |  |  |  |  |  |  |  |  |
| DF | 1.000 |  |  |  |  |  |  |  |  |
| p-value | < 0.0001 |  |  |  |  |  |  |  |  |
| alpha | 0.05 |  |  |  |  |  |  |  |  |
| The p-value has been computed using 10000 Monte Carlo simulations. Time elapsed: 6s. | | | | | | | | |  |
|  |  |  |  |  |  |  |  |  |  |
| Test interpretation: | |  |  |  |  |  |  |  |  |
| H0: The medians of Fine dining restaurants and Food supermarkets are equal. | | | | | | | |  |  |
| Ha: Medians of Fine dining restaurants and Food supermarkets are not equal | | | | | | | |  |  |
| As the computed p-value is lower than the significance level alpha=0.05, one should reject the null hypothesis H0, and accept the alternative hypothesis Ha. | | | | | | | | |  |
|  |  |  |  |  |  |  |  |  |  |
|  |  |  |  |  |  |  |  |  |  |
|  |  |  |  |  |  |  |  |  |  |
| 95% confidence interval on the p-value: | | | |  |  |  |  |  |  |
| ] 0.000, | 0.000 [ |  |  |  |  |  |  |  |  |
|  |  |  |  |  |  |  |  |  |  |
| Mood test (Fine dining restaurants\|Retail shops (non food)): | | | | | |  |  |  |  |
|  |  |  |  |  |  |  |  |  |  |
| U | 75.330 |  |  |  |  |  |  |  |  |
| Critical value | 3.841 |  |  |  |  |  |  |  |  |
| DF | 1.000 |  |  |  |  |  |  |  |  |
| p-value | < 0.0001 |  |  |  |  |  |  |  |  |
| alpha | 0.05 |  |  |  |  |  |  |  |  |
| The p-value has been computed using 10000 Monte Carlo simulations. Time elapsed: 6s. | | | | | | | | |  |
|  |  |  |  |  |  |  |  |  |  |
| Test interpretation: | |  |  |  |  |  |  |  |  |
| H0: The medians of Fine dining restaurants and Retail shops (non food) are equal. | | | | | | | |  |  |
| Ha: Medians of Fine dining restaurants and Retail shops (non food) are not equal | | | | | | | |  |  |
| As the computed p-value is lower than the significance level alpha=0.05, one should reject the null hypothesis H0, and accept the alternative hypothesis Ha. | | | | | | | | |  |
|  |  |  |  |  |  |  |  |  |  |
|  |  |  |  |  |  |  |  |  |  |
|  |  |  |  |  |  |  |  |  |  |
| 95% confidence interval on the p-value: | | | |  |  |  |  |  |  |
| ] 0.000, | 0.000 [ |  |  |  |  |  |  |  |  |
|  |  |  |  |  |  |  |  |  |  |
| Mood test (Fine dining restaurants\|Fast-food restaurants): | | | | | |  |  |  |  |
|  |  |  |  |  |  |  |  |  |  |
| U | 39.745 |  |  |  |  |  |  |  |  |
| Critical value | 3.841 |  |  |  |  |  |  |  |  |
| DF | 1.000 |  |  |  |  |  |  |  |  |
| p-value | < 0.0001 |  |  |  |  |  |  |  |  |
| alpha | 0.05 |  |  |  |  |  |  |  |  |
| The p-value has been computed using 10000 Monte Carlo simulations. Time elapsed: 6s. | | | | | | | | |  |
|  |  |  |  |  |  |  |  |  |  |
| Test interpretation: | |  |  |  |  |  |  |  |  |
| H0: The medians of Fine dining restaurants and Fast-food restaurants are equal. | | | | | | | |  |  |
| Ha: Medians of Fine dining restaurants and Fast-food restaurants are not equal | | | | | | | |  |  |
| As the computed p-value is lower than the significance level alpha=0.05, one should reject the null hypothesis H0, and accept the alternative hypothesis Ha. | | | | | | | | |  |
|  |  |  |  |  |  |  |  |  |  |
|  |  |  |  |  |  |  |  |  |  |
|  |  |  |  |  |  |  |  |  |  |
| 95% confidence interval on the p-value: | | | |  |  |  |  |  |  |
| ] 0.000, | 0.000 [ |  |  |  |  |  |  |  |  |
|  |  |  |  |  |  |  |  |  |  |
| Mood test (Fine dining restaurants\|Coffee shops): | | | | |  |  |  |  |  |
|  |  |  |  |  |  |  |  |  |  |
| U | 63.544 |  |  |  |  |  |  |  |  |
| Critical value | 3.841 |  |  |  |  |  |  |  |  |
| DF | 1.000 |  |  |  |  |  |  |  |  |
| p-value | < 0.0001 |  |  |  |  |  |  |  |  |
| alpha | 0.05 |  |  |  |  |  |  |  |  |
| The p-value has been computed using 10000 Monte Carlo simulations. Time elapsed: 6s. | | | | | | | | |  |
|  |  |  |  |  |  |  |  |  |  |
| Test interpretation: | |  |  |  |  |  |  |  |  |
| H0: The medians of Fine dining restaurants and Coffee shops are equal. | | | | | | |  |  |  |
| Ha: Medians of Fine dining restaurants and Coffee shops are not equal | | | | | | |  |  |  |
| As the computed p-value is lower than the significance level alpha=0.05, one should reject the null hypothesis H0, and accept the alternative hypothesis Ha. | | | | | | | | |  |
|  |  |  |  |  |  |  |  |  |  |
|  |  |  |  |  |  |  |  |  |  |
|  |  |  |  |  |  |  |  |  |  |
| 95% confidence interval on the p-value: | | | |  |  |  |  |  |  |
| ] 0.000, | 0.000 [ |  |  |  |  |  |  |  |  |
|  |  |  |  |  |  |  |  |  |  |
| Mood test (Fine dining restaurants\|Banks): | | | | |  |  |  |  |  |
|  |  |  |  |  |  |  |  |  |  |
| U | 71.990 |  |  |  |  |  |  |  |  |
| Critical value | 3.841 |  |  |  |  |  |  |  |  |
| DF | 1.000 |  |  |  |  |  |  |  |  |
| p-value | < 0.0001 |  |  |  |  |  |  |  |  |
| alpha | 0.05 |  |  |  |  |  |  |  |  |
| The p-value has been computed using 10000 Monte Carlo simulations. Time elapsed: 6s. | | | | | | | | |  |
|  |  |  |  |  |  |  |  |  |  |
| Test interpretation: | |  |  |  |  |  |  |  |  |
| H0: The medians of Fine dining restaurants and Banks are equal. | | | | | | |  |  |  |
| Ha: Medians of Fine dining restaurants and Banks are not equal | | | | | | |  |  |  |
| As the computed p-value is lower than the significance level alpha=0.05, one should reject the null hypothesis H0, and accept the alternative hypothesis Ha. | | | | | | | | |  |
|  |  |  |  |  |  |  |  |  |  |
|  |  |  |  |  |  |  |  |  |  |
|  |  |  |  |  |  |  |  |  |  |
| 95% confidence interval on the p-value: | | | |  |  |  |  |  |  |
| ] 0.000, | 0.000 [ |  |  |  |  |  |  |  |  |
|  |  |  |  |  |  |  |  |  |  |
| Mood test (Fine dining restaurants\|Pharmacies): | | | | |  |  |  |  |  |
|  |  |  |  |  |  |  |  |  |  |
| U | 104.528 |  |  |  |  |  |  |  |  |
| Critical value | 3.841 |  |  |  |  |  |  |  |  |
| DF | 1.000 |  |  |  |  |  |  |  |  |
| p-value | < 0.0001 |  |  |  |  |  |  |  |  |
| alpha | 0.05 |  |  |  |  |  |  |  |  |
| The p-value has been computed using 10000 Monte Carlo simulations. Time elapsed: 6s. | | | | | | | | |  |
|  |  |  |  |  |  |  |  |  |  |
| Test interpretation: | |  |  |  |  |  |  |  |  |
| H0: The medians of Fine dining restaurants and Pharmacies are equal. | | | | | | |  |  |  |
| Ha: Medians of Fine dining restaurants and Pharmacies are not equal | | | | | | |  |  |  |
| As the computed p-value is lower than the significance level alpha=0.05, one should reject the null hypothesis H0, and accept the alternative hypothesis Ha. | | | | | | | | |  |
|  |  |  |  |  |  |  |  |  |  |
|  |  |  |  |  |  |  |  |  |  |
|  |  |  |  |  |  |  |  |  |  |
| 95% confidence interval on the p-value: | | | |  |  |  |  |  |  |
| ] 0.000, | 0.000 [ |  |  |  |  |  |  |  |  |
|  |  |  |  |  |  |  |  |  |  |
| Mood test (Fine dining restaurants\|Post Offices): | | | | |  |  |  |  |  |
|  |  |  |  |  |  |  |  |  |  |
| U | 64.771 |  |  |  |  |  |  |  |  |
| Critical value | 3.841 |  |  |  |  |  |  |  |  |
| DF | 1.000 |  |  |  |  |  |  |  |  |
| p-value | < 0.0001 |  |  |  |  |  |  |  |  |
| alpha | 0.05 |  |  |  |  |  |  |  |  |
| The p-value has been computed using 10000 Monte Carlo simulations. Time elapsed: 6s. | | | | | | | | |  |
|  |  |  |  |  |  |  |  |  |  |
| Test interpretation: | |  |  |  |  |  |  |  |  |
| H0: The medians of Fine dining restaurants and Post Offices are equal. | | | | | | |  |  |  |
| Ha: Medians of Fine dining restaurants and Post Offices are not equal | | | | | | |  |  |  |
| As the computed p-value is lower than the significance level alpha=0.05, one should reject the null hypothesis H0, and accept the alternative hypothesis Ha. | | | | | | | | |  |
|  |  |  |  |  |  |  |  |  |  |
|  |  |  |  |  |  |  |  |  |  |
|  |  |  |  |  |  |  |  |  |  |
| 95% confidence interval on the p-value: | | | |  |  |  |  |  |  |
| ] 0.000, | 0.000 [ |  |  |  |  |  |  |  |  |
|  |  |  |  |  |  |  |  |  |  |
| Mood test (Fine dining restaurants\|Gas stations): | | | | |  |  |  |  |  |
|  |  |  |  |  |  |  |  |  |  |
| U | 35.995 |  |  |  |  |  |  |  |  |
| Critical value | 3.841 |  |  |  |  |  |  |  |  |
| DF | 1.000 |  |  |  |  |  |  |  |  |
| p-value | < 0.0001 |  |  |  |  |  |  |  |  |
| alpha | 0.05 |  |  |  |  |  |  |  |  |
| The p-value has been computed using 10000 Monte Carlo simulations. Time elapsed: 6s. | | | | | | | | |  |
|  |  |  |  |  |  |  |  |  |  |
| Test interpretation: | |  |  |  |  |  |  |  |  |
| H0: The medians of Fine dining restaurants and Gas stations are equal. | | | | | | |  |  |  |
| Ha: Medians of Fine dining restaurants and Gas stations are not equal | | | | | | |  |  |  |
| As the computed p-value is lower than the significance level alpha=0.05, one should reject the null hypothesis H0, and accept the alternative hypothesis Ha. | | | | | | | | |  |
|  |  |  |  |  |  |  |  |  |  |
|  |  |  |  |  |  |  |  |  |  |
|  |  |  |  |  |  |  |  |  |  |
| 95% confidence interval on the p-value: | | | |  |  |  |  |  |  |
| ] 0.000, | 0.000 [ |  |  |  |  |  |  |  |  |
|  |  |  |  |  |  |  |  |  |  |
| Mood test (Pubs and wine bars\|Hair salons): | | | | |  |  |  |  |  |
|  |  |  |  |  |  |  |  |  |  |
| U | 0.083 |  |  |  |  |  |  |  |  |
| Critical value | 3.841 |  |  |  |  |  |  |  |  |
| DF | 1.000 |  |  |  |  |  |  |  |  |
| p-value | 0.752 |  |  |  |  |  |  |  |  |
| alpha | 0.05 |  |  |  |  |  |  |  |  |
| The p-value has been computed using 10000 Monte Carlo simulations. Time elapsed: 6s. | | | | | | | | |  |
|  |  |  |  |  |  |  |  |  |  |
| Test interpretation: | |  |  |  |  |  |  |  |  |
| H0: The medians of Pubs and wine bars and Hair salons are equal. | | | | | | |  |  |  |
| Ha: Medians of Pubs and wine bars and Hair salons are not equal | | | | | | |  |  |  |
| As the computed p-value is greater than the significance level alpha=0.05, one cannot reject the null hypothesis H0. | | | | | | | | |  |
|  |  |  |  |  |  |  |  |  |  |
|  |  |  |  |  |  |  |  |  |  |
|  |  |  |  |  |  |  |  |  |  |
| 95% confidence interval on the p-value: | | | |  |  |  |  |  |  |
| ] 0.744, | 0.761 [ |  |  |  |  |  |  |  |  |
|  |  |  |  |  |  |  |  |  |  |
| Mood test (Pubs and wine bars\|Shopping centres): | | | | |  |  |  |  |  |
|  |  |  |  |  |  |  |  |  |  |
| U | 11.155 |  |  |  |  |  |  |  |  |
| Critical value | 3.841 |  |  |  |  |  |  |  |  |
| DF | 1.000 |  |  |  |  |  |  |  |  |
| p-value | 0.000 |  |  |  |  |  |  |  |  |
| alpha | 0.05 |  |  |  |  |  |  |  |  |
| The p-value has been computed using 10000 Monte Carlo simulations. Time elapsed: 6s. | | | | | | | | |  |
|  |  |  |  |  |  |  |  |  |  |
| Test interpretation: | |  |  |  |  |  |  |  |  |
| H0: The medians of Pubs and wine bars and Shopping centres are equal. | | | | | | |  |  |  |
| Ha: Medians of Pubs and wine bars and Shopping centres are not equal | | | | | | |  |  |  |
| As the computed p-value is lower than the significance level alpha=0.05, one should reject the null hypothesis H0, and accept the alternative hypothesis Ha. | | | | | | | | |  |
|  |  |  |  |  |  |  |  |  |  |
|  |  |  |  |  |  |  |  |  |  |
|  |  |  |  |  |  |  |  |  |  |
| 95% confidence interval on the p-value: | | | |  |  |  |  |  |  |
| ] 0.000, | 0.000 [ |  |  |  |  |  |  |  |  |
|  |  |  |  |  |  |  |  |  |  |
| Mood test (Pubs and wine bars\|Pizza restaurants): | | | | |  |  |  |  |  |
|  |  |  |  |  |  |  |  |  |  |
| U | 0.207 |  |  |  |  |  |  |  |  |
| Critical value | 3.841 |  |  |  |  |  |  |  |  |
| DF | 1.000 |  |  |  |  |  |  |  |  |
| p-value | 0.675 |  |  |  |  |  |  |  |  |
| alpha | 0.05 |  |  |  |  |  |  |  |  |
| The p-value has been computed using 10000 Monte Carlo simulations. Time elapsed: 6s. | | | | | | | | |  |
|  |  |  |  |  |  |  |  |  |  |
| Test interpretation: | |  |  |  |  |  |  |  |  |
| H0: The medians of Pubs and wine bars and Pizza restaurants are equal. | | | | | | |  |  |  |
| Ha: Medians of Pubs and wine bars and Pizza restaurants are not equal | | | | | | |  |  |  |
| As the computed p-value is greater than the significance level alpha=0.05, one cannot reject the null hypothesis H0. | | | | | | | | |  |
|  |  |  |  |  |  |  |  |  |  |
|  |  |  |  |  |  |  |  |  |  |
|  |  |  |  |  |  |  |  |  |  |
| 95% confidence interval on the p-value: | | | |  |  |  |  |  |  |
| ] 0.666, | 0.684 [ |  |  |  |  |  |  |  |  |
|  |  |  |  |  |  |  |  |  |  |
| Mood test (Pubs and wine bars\|Gyms): | | | |  |  |  |  |  |  |
|  |  |  |  |  |  |  |  |  |  |
| U | 4.838 |  |  |  |  |  |  |  |  |
| Critical value | 3.841 |  |  |  |  |  |  |  |  |
| DF | 1.000 |  |  |  |  |  |  |  |  |
| p-value | 0.026 |  |  |  |  |  |  |  |  |
| alpha | 0.05 |  |  |  |  |  |  |  |  |
| The p-value has been computed using 10000 Monte Carlo simulations. Time elapsed: 6s. | | | | | | | | |  |
|  |  |  |  |  |  |  |  |  |  |
| Test interpretation: | |  |  |  |  |  |  |  |  |
| H0: The medians of Pubs and wine bars and Gyms are equal. | | | | | |  |  |  |  |
| Ha: Medians of Pubs and wine bars and Gyms are not equal | | | | | |  |  |  |  |
| As the computed p-value is lower than the significance level alpha=0.05, one should reject the null hypothesis H0, and accept the alternative hypothesis Ha. | | | | | | | | |  |
|  |  |  |  |  |  |  |  |  |  |
|  |  |  |  |  |  |  |  |  |  |
|  |  |  |  |  |  |  |  |  |  |
| 95% confidence interval on the p-value: | | | |  |  |  |  |  |  |
| ] 0.023, | 0.029 [ |  |  |  |  |  |  |  |  |
|  |  |  |  |  |  |  |  |  |  |
| Mood test (Pubs and wine bars\|Food supermarkets): | | | | | |  |  |  |  |
|  |  |  |  |  |  |  |  |  |  |
| U | 41.690 |  |  |  |  |  |  |  |  |
| Critical value | 3.841 |  |  |  |  |  |  |  |  |
| DF | 1.000 |  |  |  |  |  |  |  |  |
| p-value | < 0.0001 |  |  |  |  |  |  |  |  |
| alpha | 0.05 |  |  |  |  |  |  |  |  |
| The p-value has been computed using 10000 Monte Carlo simulations. Time elapsed: 6s. | | | | | | | | |  |
|  |  |  |  |  |  |  |  |  |  |
| Test interpretation: | |  |  |  |  |  |  |  |  |
| H0: The medians of Pubs and wine bars and Food supermarkets are equal. | | | | | | | |  |  |
| Ha: Medians of Pubs and wine bars and Food supermarkets are not equal | | | | | | | |  |  |
| As the computed p-value is lower than the significance level alpha=0.05, one should reject the null hypothesis H0, and accept the alternative hypothesis Ha. | | | | | | | | |  |
|  |  |  |  |  |  |  |  |  |  |
|  |  |  |  |  |  |  |  |  |  |
|  |  |  |  |  |  |  |  |  |  |
| 95% confidence interval on the p-value: | | | |  |  |  |  |  |  |
| ] 0.000, | 0.000 [ |  |  |  |  |  |  |  |  |
|  |  |  |  |  |  |  |  |  |  |
| Mood test (Pubs and wine bars\|Retail shops (non food)): | | | | | |  |  |  |  |
|  |  |  |  |  |  |  |  |  |  |
| U | 8.182 |  |  |  |  |  |  |  |  |
| Critical value | 3.841 |  |  |  |  |  |  |  |  |
| DF | 1.000 |  |  |  |  |  |  |  |  |
| p-value | 0.003 |  |  |  |  |  |  |  |  |
| alpha | 0.05 |  |  |  |  |  |  |  |  |
| The p-value has been computed using 10000 Monte Carlo simulations. Time elapsed: 6s. | | | | | | | | |  |
|  |  |  |  |  |  |  |  |  |  |
| Test interpretation: | |  |  |  |  |  |  |  |  |
| H0: The medians of Pubs and wine bars and Retail shops (non food) are equal. | | | | | | | |  |  |
| Ha: Medians of Pubs and wine bars and Retail shops (non food) are not equal | | | | | | | |  |  |
| As the computed p-value is lower than the significance level alpha=0.05, one should reject the null hypothesis H0, and accept the alternative hypothesis Ha. | | | | | | | | |  |
|  |  |  |  |  |  |  |  |  |  |
|  |  |  |  |  |  |  |  |  |  |
|  |  |  |  |  |  |  |  |  |  |
| 95% confidence interval on the p-value: | | | |  |  |  |  |  |  |
| ] 0.002, | 0.004 [ |  |  |  |  |  |  |  |  |
|  |  |  |  |  |  |  |  |  |  |
| Mood test (Pubs and wine bars\|Fast-food restaurants): | | | | | |  |  |  |  |
|  |  |  |  |  |  |  |  |  |  |
| U | 8.413 |  |  |  |  |  |  |  |  |
| Critical value | 3.841 |  |  |  |  |  |  |  |  |
| DF | 1.000 |  |  |  |  |  |  |  |  |
| p-value | 0.004 |  |  |  |  |  |  |  |  |
| alpha | 0.05 |  |  |  |  |  |  |  |  |
| The p-value has been computed using 10000 Monte Carlo simulations. Time elapsed: 6s. | | | | | | | | |  |
|  |  |  |  |  |  |  |  |  |  |
| Test interpretation: | |  |  |  |  |  |  |  |  |
| H0: The medians of Pubs and wine bars and Fast-food restaurants are equal. | | | | | | | |  |  |
| Ha: Medians of Pubs and wine bars and Fast-food restaurants are not equal | | | | | | | |  |  |
| As the computed p-value is lower than the significance level alpha=0.05, one should reject the null hypothesis H0, and accept the alternative hypothesis Ha. | | | | | | | | |  |
|  |  |  |  |  |  |  |  |  |  |
|  |  |  |  |  |  |  |  |  |  |
|  |  |  |  |  |  |  |  |  |  |
| 95% confidence interval on the p-value: | | | |  |  |  |  |  |  |
| ] 0.003, | 0.005 [ |  |  |  |  |  |  |  |  |
|  |  |  |  |  |  |  |  |  |  |
| Mood test (Pubs and wine bars\|Coffee shops): | | | | |  |  |  |  |  |
|  |  |  |  |  |  |  |  |  |  |
| U | 15.298 |  |  |  |  |  |  |  |  |
| Critical value | 3.841 |  |  |  |  |  |  |  |  |
| DF | 1.000 |  |  |  |  |  |  |  |  |
| p-value | 0.000 |  |  |  |  |  |  |  |  |
| alpha | 0.05 |  |  |  |  |  |  |  |  |
| The p-value has been computed using 10000 Monte Carlo simulations. Time elapsed: 6s. | | | | | | | | |  |
|  |  |  |  |  |  |  |  |  |  |
| Test interpretation: | |  |  |  |  |  |  |  |  |
| H0: The medians of Pubs and wine bars and Coffee shops are equal. | | | | | | |  |  |  |
| Ha: Medians of Pubs and wine bars and Coffee shops are not equal | | | | | | |  |  |  |
| As the computed p-value is lower than the significance level alpha=0.05, one should reject the null hypothesis H0, and accept the alternative hypothesis Ha. | | | | | | | | |  |
|  |  |  |  |  |  |  |  |  |  |
|  |  |  |  |  |  |  |  |  |  |
|  |  |  |  |  |  |  |  |  |  |
| 95% confidence interval on the p-value: | | | |  |  |  |  |  |  |
| ] 0.000, | 0.000 [ |  |  |  |  |  |  |  |  |
|  |  |  |  |  |  |  |  |  |  |
| Mood test (Pubs and wine bars\|Banks): | | | |  |  |  |  |  |  |
|  |  |  |  |  |  |  |  |  |  |
| U | 20.950 |  |  |  |  |  |  |  |  |
| Critical value | 3.841 |  |  |  |  |  |  |  |  |
| DF | 1.000 |  |  |  |  |  |  |  |  |
| p-value | < 0.0001 |  |  |  |  |  |  |  |  |
| alpha | 0.05 |  |  |  |  |  |  |  |  |
| The p-value has been computed using 10000 Monte Carlo simulations. Time elapsed: 6s. | | | | | | | | |  |
|  |  |  |  |  |  |  |  |  |  |
| Test interpretation: | |  |  |  |  |  |  |  |  |
| H0: The medians of Pubs and wine bars and Banks are equal. | | | | | |  |  |  |  |
| Ha: Medians of Pubs and wine bars and Banks are not equal | | | | | |  |  |  |  |
| As the computed p-value is lower than the significance level alpha=0.05, one should reject the null hypothesis H0, and accept the alternative hypothesis Ha. | | | | | | | | |  |
|  |  |  |  |  |  |  |  |  |  |
|  |  |  |  |  |  |  |  |  |  |
|  |  |  |  |  |  |  |  |  |  |
| 95% confidence interval on the p-value: | | | |  |  |  |  |  |  |
| ] 0.000, | 0.000 [ |  |  |  |  |  |  |  |  |
|  |  |  |  |  |  |  |  |  |  |
| Mood test (Pubs and wine bars\|Pharmacies): | | | | |  |  |  |  |  |
|  |  |  |  |  |  |  |  |  |  |
| U | 68.099 |  |  |  |  |  |  |  |  |
| Critical value | 3.841 |  |  |  |  |  |  |  |  |
| DF | 1.000 |  |  |  |  |  |  |  |  |
| p-value | < 0.0001 |  |  |  |  |  |  |  |  |
| alpha | 0.05 |  |  |  |  |  |  |  |  |
| The p-value has been computed using 10000 Monte Carlo simulations. Time elapsed: 6s. | | | | | | | | |  |
|  |  |  |  |  |  |  |  |  |  |
| Test interpretation: | |  |  |  |  |  |  |  |  |
| H0: The medians of Pubs and wine bars and Pharmacies are equal. | | | | | | |  |  |  |
| Ha: Medians of Pubs and wine bars and Pharmacies are not equal | | | | | | |  |  |  |
| As the computed p-value is lower than the significance level alpha=0.05, one should reject the null hypothesis H0, and accept the alternative hypothesis Ha. | | | | | | | | |  |
|  |  |  |  |  |  |  |  |  |  |
|  |  |  |  |  |  |  |  |  |  |
|  |  |  |  |  |  |  |  |  |  |
| 95% confidence interval on the p-value: | | | |  |  |  |  |  |  |
| ] 0.000, | 0.000 [ |  |  |  |  |  |  |  |  |
|  |  |  |  |  |  |  |  |  |  |
| Mood test (Pubs and wine bars\|Post Offices): | | | | |  |  |  |  |  |
|  |  |  |  |  |  |  |  |  |  |
| U | 25.263 |  |  |  |  |  |  |  |  |
| Critical value | 3.841 |  |  |  |  |  |  |  |  |
| DF | 1.000 |  |  |  |  |  |  |  |  |
| p-value | < 0.0001 |  |  |  |  |  |  |  |  |
| alpha | 0.05 |  |  |  |  |  |  |  |  |
| The p-value has been computed using 10000 Monte Carlo simulations. Time elapsed: 6s. | | | | | | | | |  |
|  |  |  |  |  |  |  |  |  |  |
| Test interpretation: | |  |  |  |  |  |  |  |  |
| H0: The medians of Pubs and wine bars and Post Offices are equal. | | | | | | |  |  |  |
| Ha: Medians of Pubs and wine bars and Post Offices are not equal | | | | | | |  |  |  |
| As the computed p-value is lower than the significance level alpha=0.05, one should reject the null hypothesis H0, and accept the alternative hypothesis Ha. | | | | | | | | |  |
|  |  |  |  |  |  |  |  |  |  |
|  |  |  |  |  |  |  |  |  |  |
|  |  |  |  |  |  |  |  |  |  |
| 95% confidence interval on the p-value: | | | |  |  |  |  |  |  |
| ] 0.000, | 0.000 [ |  |  |  |  |  |  |  |  |
|  |  |  |  |  |  |  |  |  |  |
| Mood test (Pubs and wine bars\|Gas stations): | | | | |  |  |  |  |  |
|  |  |  |  |  |  |  |  |  |  |
| U | 33.474 |  |  |  |  |  |  |  |  |
| Critical value | 3.841 |  |  |  |  |  |  |  |  |
| DF | 1.000 |  |  |  |  |  |  |  |  |
| p-value | < 0.0001 |  |  |  |  |  |  |  |  |
| alpha | 0.05 |  |  |  |  |  |  |  |  |
| The p-value has been computed using 10000 Monte Carlo simulations. Time elapsed: 6s. | | | | | | | | |  |
|  |  |  |  |  |  |  |  |  |  |
| Test interpretation: | |  |  |  |  |  |  |  |  |
| H0: The medians of Pubs and wine bars and Gas stations are equal. | | | | | | |  |  |  |
| Ha: Medians of Pubs and wine bars and Gas stations are not equal | | | | | | |  |  |  |
| As the computed p-value is lower than the significance level alpha=0.05, one should reject the null hypothesis H0, and accept the alternative hypothesis Ha. | | | | | | | | |  |
|  |  |  |  |  |  |  |  |  |  |
|  |  |  |  |  |  |  |  |  |  |
|  |  |  |  |  |  |  |  |  |  |
| 95% confidence interval on the p-value: | | | |  |  |  |  |  |  |
| ] 0.000, | 0.000 [ |  |  |  |  |  |  |  |  |
|  |  |  |  |  |  |  |  |  |  |
| Mood test (Hair salons\|Shopping centres): | | | | |  |  |  |  |  |
|  |  |  |  |  |  |  |  |  |  |
| U | 15.281 |  |  |  |  |  |  |  |  |
| Critical value | 3.841 |  |  |  |  |  |  |  |  |
| DF | 1.000 |  |  |  |  |  |  |  |  |
| p-value | < 0.0001 |  |  |  |  |  |  |  |  |
| alpha | 0.05 |  |  |  |  |  |  |  |  |
| The p-value has been computed using 10000 Monte Carlo simulations. Time elapsed: 6s. | | | | | | | | |  |
|  |  |  |  |  |  |  |  |  |  |
| Test interpretation: | |  |  |  |  |  |  |  |  |
| H0: The medians of Hair salons and Shopping centres are equal. | | | | | | |  |  |  |
| Ha: Medians of Hair salons and Shopping centres are not equal | | | | | | |  |  |  |
| As the computed p-value is lower than the significance level alpha=0.05, one should reject the null hypothesis H0, and accept the alternative hypothesis Ha. | | | | | | | | |  |
|  |  |  |  |  |  |  |  |  |  |
|  |  |  |  |  |  |  |  |  |  |
|  |  |  |  |  |  |  |  |  |  |
| 95% confidence interval on the p-value: | | | |  |  |  |  |  |  |
| ] 0.000, | 0.000 [ |  |  |  |  |  |  |  |  |
|  |  |  |  |  |  |  |  |  |  |
| Mood test (Hair salons\|Pizza restaurants): | | | | |  |  |  |  |  |
|  |  |  |  |  |  |  |  |  |  |
| U | 0.034 |  |  |  |  |  |  |  |  |
| Critical value | 3.841 |  |  |  |  |  |  |  |  |
| DF | 1.000 |  |  |  |  |  |  |  |  |
| p-value | 0.850 |  |  |  |  |  |  |  |  |
| alpha | 0.05 |  |  |  |  |  |  |  |  |
| The p-value has been computed using 10000 Monte Carlo simulations. Time elapsed: 6s. | | | | | | | | |  |
|  |  |  |  |  |  |  |  |  |  |
| Test interpretation: | |  |  |  |  |  |  |  |  |
| H0: The medians of Hair salons and Pizza restaurants are equal. | | | | | | |  |  |  |
| Ha: Medians of Hair salons and Pizza restaurants are not equal | | | | | | |  |  |  |
| As the computed p-value is greater than the significance level alpha=0.05, one cannot reject the null hypothesis H0. | | | | | | | | |  |
|  |  |  |  |  |  |  |  |  |  |
|  |  |  |  |  |  |  |  |  |  |
|  |  |  |  |  |  |  |  |  |  |
| 95% confidence interval on the p-value: | | | |  |  |  |  |  |  |
| ] 0.843, | 0.857 [ |  |  |  |  |  |  |  |  |
|  |  |  |  |  |  |  |  |  |  |
| Mood test (Hair salons\|Gyms): | | | |  |  |  |  |  |  |
|  |  |  |  |  |  |  |  |  |  |
| U | 10.155 |  |  |  |  |  |  |  |  |
| Critical value | 3.841 |  |  |  |  |  |  |  |  |
| DF | 1.000 |  |  |  |  |  |  |  |  |
| p-value | 0.002 |  |  |  |  |  |  |  |  |
| alpha | 0.05 |  |  |  |  |  |  |  |  |
| The p-value has been computed using 10000 Monte Carlo simulations. Time elapsed: 6s. | | | | | | | | |  |
|  |  |  |  |  |  |  |  |  |  |
| Test interpretation: | |  |  |  |  |  |  |  |  |
| H0: The medians of Hair salons and Gyms are equal. | | | | | |  |  |  |  |
| Ha: Medians of Hair salons and Gyms are not equal | | | | |  |  |  |  |  |
| As the computed p-value is lower than the significance level alpha=0.05, one should reject the null hypothesis H0, and accept the alternative hypothesis Ha. | | | | | | | | |  |
|  |  |  |  |  |  |  |  |  |  |
|  |  |  |  |  |  |  |  |  |  |
|  |  |  |  |  |  |  |  |  |  |
| 95% confidence interval on the p-value: | | | |  |  |  |  |  |  |
| ] 0.001, | 0.003 [ |  |  |  |  |  |  |  |  |
|  |  |  |  |  |  |  |  |  |  |
| Mood test (Hair salons\|Food supermarkets): | | | | |  |  |  |  |  |
|  |  |  |  |  |  |  |  |  |  |
| U | 38.875 |  |  |  |  |  |  |  |  |
| Critical value | 3.841 |  |  |  |  |  |  |  |  |
| DF | 1.000 |  |  |  |  |  |  |  |  |
| p-value | < 0.0001 |  |  |  |  |  |  |  |  |
| alpha | 0.05 |  |  |  |  |  |  |  |  |
| The p-value has been computed using 10000 Monte Carlo simulations. Time elapsed: 6s. | | | | | | | | |  |
|  |  |  |  |  |  |  |  |  |  |
| Test interpretation: | |  |  |  |  |  |  |  |  |
| H0: The medians of Hair salons and Food supermarkets are equal. | | | | | | |  |  |  |
| Ha: Medians of Hair salons and Food supermarkets are not equal | | | | | | |  |  |  |
| As the computed p-value is lower than the significance level alpha=0.05, one should reject the null hypothesis H0, and accept the alternative hypothesis Ha. | | | | | | | | |  |
|  |  |  |  |  |  |  |  |  |  |
|  |  |  |  |  |  |  |  |  |  |
|  |  |  |  |  |  |  |  |  |  |
| 95% confidence interval on the p-value: | | | |  |  |  |  |  |  |
| ] 0.000, | 0.000 [ |  |  |  |  |  |  |  |  |
|  |  |  |  |  |  |  |  |  |  |
| Mood test (Hair salons\|Retail shops (non food)): | | | | |  |  |  |  |  |
|  |  |  |  |  |  |  |  |  |  |
| U | 9.991 |  |  |  |  |  |  |  |  |
| Critical value | 3.841 |  |  |  |  |  |  |  |  |
| DF | 1.000 |  |  |  |  |  |  |  |  |
| p-value | 0.002 |  |  |  |  |  |  |  |  |
| alpha | 0.05 |  |  |  |  |  |  |  |  |
| The p-value has been computed using 10000 Monte Carlo simulations. Time elapsed: 6s. | | | | | | | | |  |
|  |  |  |  |  |  |  |  |  |  |
| Test interpretation: | |  |  |  |  |  |  |  |  |
| H0: The medians of Hair salons and Retail shops (non food) are equal. | | | | | | |  |  |  |
| Ha: Medians of Hair salons and Retail shops (non food) are not equal | | | | | | |  |  |  |
| As the computed p-value is lower than the significance level alpha=0.05, one should reject the null hypothesis H0, and accept the alternative hypothesis Ha. | | | | | | | | |  |
|  |  |  |  |  |  |  |  |  |  |
|  |  |  |  |  |  |  |  |  |  |
|  |  |  |  |  |  |  |  |  |  |
| 95% confidence interval on the p-value: | | | |  |  |  |  |  |  |
| ] 0.001, | 0.002 [ |  |  |  |  |  |  |  |  |
|  |  |  |  |  |  |  |  |  |  |
| Mood test (Hair salons\|Fast-food restaurants): | | | | |  |  |  |  |  |
|  |  |  |  |  |  |  |  |  |  |
| U | 10.462 |  |  |  |  |  |  |  |  |
| Critical value | 3.841 |  |  |  |  |  |  |  |  |
| DF | 1.000 |  |  |  |  |  |  |  |  |
| p-value | 0.001 |  |  |  |  |  |  |  |  |
| alpha | 0.05 |  |  |  |  |  |  |  |  |
| The p-value has been computed using 10000 Monte Carlo simulations. Time elapsed: 6s. | | | | | | | | |  |
|  |  |  |  |  |  |  |  |  |  |
| Test interpretation: | |  |  |  |  |  |  |  |  |
| H0: The medians of Hair salons and Fast-food restaurants are equal. | | | | | | |  |  |  |
| Ha: Medians of Hair salons and Fast-food restaurants are not equal | | | | | | |  |  |  |
| As the computed p-value is lower than the significance level alpha=0.05, one should reject the null hypothesis H0, and accept the alternative hypothesis Ha. | | | | | | | | |  |
|  |  |  |  |  |  |  |  |  |  |
|  |  |  |  |  |  |  |  |  |  |
|  |  |  |  |  |  |  |  |  |  |
| 95% confidence interval on the p-value: | | | |  |  |  |  |  |  |
| ] 0.000, | 0.001 [ |  |  |  |  |  |  |  |  |
|  |  |  |  |  |  |  |  |  |  |
| Mood test (Hair salons\|Coffee shops): | | | |  |  |  |  |  |  |
|  |  |  |  |  |  |  |  |  |  |
| U | 16.679 |  |  |  |  |  |  |  |  |
| Critical value | 3.841 |  |  |  |  |  |  |  |  |
| DF | 1.000 |  |  |  |  |  |  |  |  |
| p-value | < 0.0001 |  |  |  |  |  |  |  |  |
| alpha | 0.05 |  |  |  |  |  |  |  |  |
| The p-value has been computed using 10000 Monte Carlo simulations. Time elapsed: 6s. | | | | | | | | |  |
|  |  |  |  |  |  |  |  |  |  |
| Test interpretation: | |  |  |  |  |  |  |  |  |
| H0: The medians of Hair salons and Coffee shops are equal. | | | | | |  |  |  |  |
| Ha: Medians of Hair salons and Coffee shops are not equal | | | | | |  |  |  |  |
| As the computed p-value is lower than the significance level alpha=0.05, one should reject the null hypothesis H0, and accept the alternative hypothesis Ha. | | | | | | | | |  |
|  |  |  |  |  |  |  |  |  |  |
|  |  |  |  |  |  |  |  |  |  |
|  |  |  |  |  |  |  |  |  |  |
| 95% confidence interval on the p-value: | | | |  |  |  |  |  |  |
| ] 0.000, | 0.000 [ |  |  |  |  |  |  |  |  |
|  |  |  |  |  |  |  |  |  |  |
| Mood test (Hair salons\|Banks): | | | |  |  |  |  |  |  |
|  |  |  |  |  |  |  |  |  |  |
| U | 15.817 |  |  |  |  |  |  |  |  |
| Critical value | 3.841 |  |  |  |  |  |  |  |  |
| DF | 1.000 |  |  |  |  |  |  |  |  |
| p-value | < 0.0001 |  |  |  |  |  |  |  |  |
| alpha | 0.05 |  |  |  |  |  |  |  |  |
| The p-value has been computed using 10000 Monte Carlo simulations. Time elapsed: 6s. | | | | | | | | |  |
|  |  |  |  |  |  |  |  |  |  |
| Test interpretation: | |  |  |  |  |  |  |  |  |
| H0: The medians of Hair salons and Banks are equal. | | | | | |  |  |  |  |
| Ha: Medians of Hair salons and Banks are not equal | | | | |  |  |  |  |  |
| As the computed p-value is lower than the significance level alpha=0.05, one should reject the null hypothesis H0, and accept the alternative hypothesis Ha. | | | | | | | | |  |
|  |  |  |  |  |  |  |  |  |  |
|  |  |  |  |  |  |  |  |  |  |
|  |  |  |  |  |  |  |  |  |  |
| 95% confidence interval on the p-value: | | | |  |  |  |  |  |  |
| ] 0.000, | 0.000 [ |  |  |  |  |  |  |  |  |
|  |  |  |  |  |  |  |  |  |  |
| Mood test (Hair salons\|Pharmacies): | | | |  |  |  |  |  |  |
|  |  |  |  |  |  |  |  |  |  |
| U | 59.919 |  |  |  |  |  |  |  |  |
| Critical value | 3.841 |  |  |  |  |  |  |  |  |
| DF | 1.000 |  |  |  |  |  |  |  |  |
| p-value | < 0.0001 |  |  |  |  |  |  |  |  |
| alpha | 0.05 |  |  |  |  |  |  |  |  |
| The p-value has been computed using 10000 Monte Carlo simulations. Time elapsed: 6s. | | | | | | | | |  |
|  |  |  |  |  |  |  |  |  |  |
| Test interpretation: | |  |  |  |  |  |  |  |  |
| H0: The medians of Hair salons and Pharmacies are equal. | | | | | |  |  |  |  |
| Ha: Medians of Hair salons and Pharmacies are not equal | | | | | |  |  |  |  |
| As the computed p-value is lower than the significance level alpha=0.05, one should reject the null hypothesis H0, and accept the alternative hypothesis Ha. | | | | | | | | |  |
|  |  |  |  |  |  |  |  |  |  |
|  |  |  |  |  |  |  |  |  |  |
|  |  |  |  |  |  |  |  |  |  |
| 95% confidence interval on the p-value: | | | |  |  |  |  |  |  |
| ] 0.000, | 0.000 [ |  |  |  |  |  |  |  |  |
|  |  |  |  |  |  |  |  |  |  |
| Mood test (Hair salons\|Post Offices): | | | |  |  |  |  |  |  |
|  |  |  |  |  |  |  |  |  |  |
| U | 26.048 |  |  |  |  |  |  |  |  |
| Critical value | 3.841 |  |  |  |  |  |  |  |  |
| DF | 1.000 |  |  |  |  |  |  |  |  |
| p-value | < 0.0001 |  |  |  |  |  |  |  |  |
| alpha | 0.05 |  |  |  |  |  |  |  |  |
| The p-value has been computed using 10000 Monte Carlo simulations. Time elapsed: 6s. | | | | | | | | |  |
|  |  |  |  |  |  |  |  |  |  |
| Test interpretation: | |  |  |  |  |  |  |  |  |
| H0: The medians of Hair salons and Post Offices are equal. | | | | | |  |  |  |  |
| Ha: Medians of Hair salons and Post Offices are not equal | | | | | |  |  |  |  |
| As the computed p-value is lower than the significance level alpha=0.05, one should reject the null hypothesis H0, and accept the alternative hypothesis Ha. | | | | | | | | |  |
|  |  |  |  |  |  |  |  |  |  |
|  |  |  |  |  |  |  |  |  |  |
|  |  |  |  |  |  |  |  |  |  |
| 95% confidence interval on the p-value: | | | |  |  |  |  |  |  |
| ] 0.000, | 0.000 [ |  |  |  |  |  |  |  |  |
|  |  |  |  |  |  |  |  |  |  |
| Mood test (Hair salons\|Gas stations): | | | |  |  |  |  |  |  |
|  |  |  |  |  |  |  |  |  |  |
| U | 32.776 |  |  |  |  |  |  |  |  |
| Critical value | 3.841 |  |  |  |  |  |  |  |  |
| DF | 1.000 |  |  |  |  |  |  |  |  |
| p-value | < 0.0001 |  |  |  |  |  |  |  |  |
| alpha | 0.05 |  |  |  |  |  |  |  |  |
| The p-value has been computed using 10000 Monte Carlo simulations. Time elapsed: 6s. | | | | | | | | |  |
|  |  |  |  |  |  |  |  |  |  |
| Test interpretation: | |  |  |  |  |  |  |  |  |
| H0: The medians of Hair salons and Gas stations are equal. | | | | | |  |  |  |  |
| Ha: Medians of Hair salons and Gas stations are not equal | | | | | |  |  |  |  |
| As the computed p-value is lower than the significance level alpha=0.05, one should reject the null hypothesis H0, and accept the alternative hypothesis Ha. | | | | | | | | |  |
|  |  |  |  |  |  |  |  |  |  |
|  |  |  |  |  |  |  |  |  |  |
|  |  |  |  |  |  |  |  |  |  |
| 95% confidence interval on the p-value: | | | |  |  |  |  |  |  |
| ] 0.000, | 0.000 [ |  |  |  |  |  |  |  |  |
|  |  |  |  |  |  |  |  |  |  |
| Mood test (Shopping centres\|Pizza restaurants): | | | | |  |  |  |  |  |
|  |  |  |  |  |  |  |  |  |  |
| U | 10.407 |  |  |  |  |  |  |  |  |
| Critical value | 3.841 |  |  |  |  |  |  |  |  |
| DF | 1.000 |  |  |  |  |  |  |  |  |
| p-value | 0.001 |  |  |  |  |  |  |  |  |
| alpha | 0.05 |  |  |  |  |  |  |  |  |
| The p-value has been computed using 10000 Monte Carlo simulations. Time elapsed: 6s. | | | | | | | | |  |
|  |  |  |  |  |  |  |  |  |  |
| Test interpretation: | |  |  |  |  |  |  |  |  |
| H0: The medians of Shopping centres and Pizza restaurants are equal. | | | | | | |  |  |  |
| Ha: Medians of Shopping centres and Pizza restaurants are not equal | | | | | | |  |  |  |
| As the computed p-value is lower than the significance level alpha=0.05, one should reject the null hypothesis H0, and accept the alternative hypothesis Ha. | | | | | | | | |  |
|  |  |  |  |  |  |  |  |  |  |
|  |  |  |  |  |  |  |  |  |  |
|  |  |  |  |  |  |  |  |  |  |
| 95% confidence interval on the p-value: | | | |  |  |  |  |  |  |
| ] 0.000, | 0.002 [ |  |  |  |  |  |  |  |  |
|  |  |  |  |  |  |  |  |  |  |
| Mood test (Shopping centres\|Gyms): | | | |  |  |  |  |  |  |
|  |  |  |  |  |  |  |  |  |  |
| U | 3.925 |  |  |  |  |  |  |  |  |
| Critical value | 3.841 |  |  |  |  |  |  |  |  |
| DF | 1.000 |  |  |  |  |  |  |  |  |
| p-value | 0.048 |  |  |  |  |  |  |  |  |
| alpha | 0.05 |  |  |  |  |  |  |  |  |
| The p-value has been computed using 10000 Monte Carlo simulations. Time elapsed: 6s. | | | | | | | | |  |
|  |  |  |  |  |  |  |  |  |  |
| Test interpretation: | |  |  |  |  |  |  |  |  |
| H0: The medians of Shopping centres and Gyms are equal. | | | | | |  |  |  |  |
| Ha: Medians of Shopping centres and Gyms are not equal | | | | | |  |  |  |  |
| As the computed p-value is lower than the significance level alpha=0.05, one should reject the null hypothesis H0, and accept the alternative hypothesis Ha. | | | | | | | | |  |
|  |  |  |  |  |  |  |  |  |  |
|  |  |  |  |  |  |  |  |  |  |
|  |  |  |  |  |  |  |  |  |  |
| 95% confidence interval on the p-value: | | | |  |  |  |  |  |  |
| ] 0.044, | 0.052 [ |  |  |  |  |  |  |  |  |
|  |  |  |  |  |  |  |  |  |  |
| Mood test (Shopping centres\|Food supermarkets): | | | | |  |  |  |  |  |
|  |  |  |  |  |  |  |  |  |  |
| U | 23.008 |  |  |  |  |  |  |  |  |
| Critical value | 3.841 |  |  |  |  |  |  |  |  |
| DF | 1.000 |  |  |  |  |  |  |  |  |
| p-value | 0.000 |  |  |  |  |  |  |  |  |
| alpha | 0.05 |  |  |  |  |  |  |  |  |
| The p-value has been computed using 10000 Monte Carlo simulations. Time elapsed: 6s. | | | | | | | | |  |
|  |  |  |  |  |  |  |  |  |  |
| Test interpretation: | |  |  |  |  |  |  |  |  |
| H0: The medians of Shopping centres and Food supermarkets are equal. | | | | | | |  |  |  |
| Ha: Medians of Shopping centres and Food supermarkets are not equal | | | | | | |  |  |  |
| As the computed p-value is lower than the significance level alpha=0.05, one should reject the null hypothesis H0, and accept the alternative hypothesis Ha. | | | | | | | | |  |
|  |  |  |  |  |  |  |  |  |  |
|  |  |  |  |  |  |  |  |  |  |
|  |  |  |  |  |  |  |  |  |  |
| 95% confidence interval on the p-value: | | | |  |  |  |  |  |  |
| ] 0.000, | 0.001 [ |  |  |  |  |  |  |  |  |
|  |  |  |  |  |  |  |  |  |  |
| Mood test (Shopping centres\|Retail shops (non food)): | | | | | |  |  |  |  |
|  |  |  |  |  |  |  |  |  |  |
| U | 3.272 |  |  |  |  |  |  |  |  |
| Critical value | 3.841 |  |  |  |  |  |  |  |  |
| DF | 1.000 |  |  |  |  |  |  |  |  |
| p-value | 0.075 |  |  |  |  |  |  |  |  |
| alpha | 0.05 |  |  |  |  |  |  |  |  |
| The p-value has been computed using 10000 Monte Carlo simulations. Time elapsed: 6s. | | | | | | | | |  |
|  |  |  |  |  |  |  |  |  |  |
| Test interpretation: | |  |  |  |  |  |  |  |  |
| H0: The medians of Shopping centres and Retail shops (non food) are equal. | | | | | | | |  |  |
| Ha: Medians of Shopping centres and Retail shops (non food) are not equal | | | | | | | |  |  |
| As the computed p-value is greater than the significance level alpha=0.05, one cannot reject the null hypothesis H0. | | | | | | | | |  |
|  |  |  |  |  |  |  |  |  |  |
|  |  |  |  |  |  |  |  |  |  |
|  |  |  |  |  |  |  |  |  |  |
| 95% confidence interval on the p-value: | | | |  |  |  |  |  |  |
| ] 0.069, | 0.080 [ |  |  |  |  |  |  |  |  |
|  |  |  |  |  |  |  |  |  |  |
| Mood test (Shopping centres\|Fast-food restaurants): | | | | | |  |  |  |  |
|  |  |  |  |  |  |  |  |  |  |
| U | 4.278 |  |  |  |  |  |  |  |  |
| Critical value | 3.841 |  |  |  |  |  |  |  |  |
| DF | 1.000 |  |  |  |  |  |  |  |  |
| p-value | 0.042 |  |  |  |  |  |  |  |  |
| alpha | 0.05 |  |  |  |  |  |  |  |  |
| The p-value has been computed using 10000 Monte Carlo simulations. Time elapsed: 6s. | | | | | | | | |  |
|  |  |  |  |  |  |  |  |  |  |
| Test interpretation: | |  |  |  |  |  |  |  |  |
| H0: The medians of Shopping centres and Fast-food restaurants are equal. | | | | | | | |  |  |
| Ha: Medians of Shopping centres and Fast-food restaurants are not equal | | | | | | | |  |  |
| As the computed p-value is lower than the significance level alpha=0.05, one should reject the null hypothesis H0, and accept the alternative hypothesis Ha. | | | | | | | | |  |
|  |  |  |  |  |  |  |  |  |  |
|  |  |  |  |  |  |  |  |  |  |
|  |  |  |  |  |  |  |  |  |  |
| 95% confidence interval on the p-value: | | | |  |  |  |  |  |  |
| ] 0.038, | 0.046 [ |  |  |  |  |  |  |  |  |
|  |  |  |  |  |  |  |  |  |  |
| Mood test (Shopping centres\|Coffee shops): | | | | |  |  |  |  |  |
|  |  |  |  |  |  |  |  |  |  |
| U | 8.261 |  |  |  |  |  |  |  |  |
| Critical value | 3.841 |  |  |  |  |  |  |  |  |
| DF | 1.000 |  |  |  |  |  |  |  |  |
| p-value | 0.003 |  |  |  |  |  |  |  |  |
| alpha | 0.05 |  |  |  |  |  |  |  |  |
| The p-value has been computed using 10000 Monte Carlo simulations. Time elapsed: 6s. | | | | | | | | |  |
|  |  |  |  |  |  |  |  |  |  |
| Test interpretation: | |  |  |  |  |  |  |  |  |
| H0: The medians of Shopping centres and Coffee shops are equal. | | | | | | |  |  |  |
| Ha: Medians of Shopping centres and Coffee shops are not equal | | | | | | |  |  |  |
| As the computed p-value is lower than the significance level alpha=0.05, one should reject the null hypothesis H0, and accept the alternative hypothesis Ha. | | | | | | | | |  |
|  |  |  |  |  |  |  |  |  |  |
|  |  |  |  |  |  |  |  |  |  |
|  |  |  |  |  |  |  |  |  |  |
| 95% confidence interval on the p-value: | | | |  |  |  |  |  |  |
| ] 0.002, | 0.004 [ |  |  |  |  |  |  |  |  |
|  |  |  |  |  |  |  |  |  |  |
| Mood test (Shopping centres\|Banks): | | | |  |  |  |  |  |  |
|  |  |  |  |  |  |  |  |  |  |
| U | 27.506 |  |  |  |  |  |  |  |  |
| Critical value | 3.841 |  |  |  |  |  |  |  |  |
| DF | 1.000 |  |  |  |  |  |  |  |  |
| p-value | < 0.0001 |  |  |  |  |  |  |  |  |
| alpha | 0.05 |  |  |  |  |  |  |  |  |
| The p-value has been computed using 10000 Monte Carlo simulations. Time elapsed: 6s. | | | | | | | | |  |
|  |  |  |  |  |  |  |  |  |  |
| Test interpretation: | |  |  |  |  |  |  |  |  |
| H0: The medians of Shopping centres and Banks are equal. | | | | | |  |  |  |  |
| Ha: Medians of Shopping centres and Banks are not equal | | | | | |  |  |  |  |
| As the computed p-value is lower than the significance level alpha=0.05, one should reject the null hypothesis H0, and accept the alternative hypothesis Ha. | | | | | | | | |  |
|  |  |  |  |  |  |  |  |  |  |
|  |  |  |  |  |  |  |  |  |  |
|  |  |  |  |  |  |  |  |  |  |
| 95% confidence interval on the p-value: | | | |  |  |  |  |  |  |
| ] 0.000, | 0.000 [ |  |  |  |  |  |  |  |  |
|  |  |  |  |  |  |  |  |  |  |
| Mood test (Shopping centres\|Pharmacies): | | | | |  |  |  |  |  |
|  |  |  |  |  |  |  |  |  |  |
| U | 79.444 |  |  |  |  |  |  |  |  |
| Critical value | 3.841 |  |  |  |  |  |  |  |  |
| DF | 1.000 |  |  |  |  |  |  |  |  |
| p-value | < 0.0001 |  |  |  |  |  |  |  |  |
| alpha | 0.05 |  |  |  |  |  |  |  |  |
| The p-value has been computed using 10000 Monte Carlo simulations. Time elapsed: 6s. | | | | | | | | |  |
|  |  |  |  |  |  |  |  |  |  |
| Test interpretation: | |  |  |  |  |  |  |  |  |
| H0: The medians of Shopping centres and Pharmacies are equal. | | | | | | |  |  |  |
| Ha: Medians of Shopping centres and Pharmacies are not equal | | | | | | |  |  |  |
| As the computed p-value is lower than the significance level alpha=0.05, one should reject the null hypothesis H0, and accept the alternative hypothesis Ha. | | | | | | | | |  |
|  |  |  |  |  |  |  |  |  |  |
|  |  |  |  |  |  |  |  |  |  |
|  |  |  |  |  |  |  |  |  |  |
| 95% confidence interval on the p-value: | | | |  |  |  |  |  |  |
| ] 0.000, | 0.000 [ |  |  |  |  |  |  |  |  |
|  |  |  |  |  |  |  |  |  |  |
| Mood test (Shopping centres\|Post Offices): | | | | |  |  |  |  |  |
|  |  |  |  |  |  |  |  |  |  |
| U | 15.238 |  |  |  |  |  |  |  |  |
| Critical value | 3.841 |  |  |  |  |  |  |  |  |
| DF | 1.000 |  |  |  |  |  |  |  |  |
| p-value | 0.000 |  |  |  |  |  |  |  |  |
| alpha | 0.05 |  |  |  |  |  |  |  |  |
| The p-value has been computed using 10000 Monte Carlo simulations. Time elapsed: 6s. | | | | | | | | |  |
|  |  |  |  |  |  |  |  |  |  |
| Test interpretation: | |  |  |  |  |  |  |  |  |
| H0: The medians of Shopping centres and Post Offices are equal. | | | | | | |  |  |  |
| Ha: Medians of Shopping centres and Post Offices are not equal | | | | | | |  |  |  |
| As the computed p-value is lower than the significance level alpha=0.05, one should reject the null hypothesis H0, and accept the alternative hypothesis Ha. | | | | | | | | |  |
|  |  |  |  |  |  |  |  |  |  |
|  |  |  |  |  |  |  |  |  |  |
|  |  |  |  |  |  |  |  |  |  |
| 95% confidence interval on the p-value: | | | |  |  |  |  |  |  |
| ] 0.000, | 0.000 [ |  |  |  |  |  |  |  |  |
|  |  |  |  |  |  |  |  |  |  |
| Mood test (Shopping centres\|Gas stations): | | | | |  |  |  |  |  |
|  |  |  |  |  |  |  |  |  |  |
| U | 36.954 |  |  |  |  |  |  |  |  |
| Critical value | 3.841 |  |  |  |  |  |  |  |  |
| DF | 1.000 |  |  |  |  |  |  |  |  |
| p-value | < 0.0001 |  |  |  |  |  |  |  |  |
| alpha | 0.05 |  |  |  |  |  |  |  |  |
| The p-value has been computed using 10000 Monte Carlo simulations. Time elapsed: 6s. | | | | | | | | |  |
|  |  |  |  |  |  |  |  |  |  |
| Test interpretation: | |  |  |  |  |  |  |  |  |
| H0: The medians of Shopping centres and Gas stations are equal. | | | | | | |  |  |  |
| Ha: Medians of Shopping centres and Gas stations are not equal | | | | | | |  |  |  |
| As the computed p-value is lower than the significance level alpha=0.05, one should reject the null hypothesis H0, and accept the alternative hypothesis Ha. | | | | | | | | |  |
|  |  |  |  |  |  |  |  |  |  |
|  |  |  |  |  |  |  |  |  |  |
|  |  |  |  |  |  |  |  |  |  |
| 95% confidence interval on the p-value: | | | |  |  |  |  |  |  |
| ] 0.000, | 0.000 [ |  |  |  |  |  |  |  |  |
|  |  |  |  |  |  |  |  |  |  |
| Mood test (Pizza restaurants\|Gyms): | | | |  |  |  |  |  |  |
|  |  |  |  |  |  |  |  |  |  |
| U | 2.268 |  |  |  |  |  |  |  |  |
| Critical value | 3.841 |  |  |  |  |  |  |  |  |
| DF | 1.000 |  |  |  |  |  |  |  |  |
| p-value | 0.118 |  |  |  |  |  |  |  |  |
| alpha | 0.05 |  |  |  |  |  |  |  |  |
| The p-value has been computed using 10000 Monte Carlo simulations. Time elapsed: 6s. | | | | | | | | |  |
|  |  |  |  |  |  |  |  |  |  |
| Test interpretation: | |  |  |  |  |  |  |  |  |
| H0: The medians of Pizza restaurants and Gyms are equal. | | | | | |  |  |  |  |
| Ha: Medians of Pizza restaurants and Gyms are not equal | | | | | |  |  |  |  |
| As the computed p-value is greater than the significance level alpha=0.05, one cannot reject the null hypothesis H0. | | | | | | | | |  |
|  |  |  |  |  |  |  |  |  |  |
|  |  |  |  |  |  |  |  |  |  |
|  |  |  |  |  |  |  |  |  |  |
| 95% confidence interval on the p-value: | | | |  |  |  |  |  |  |
| ] 0.111, | 0.124 [ |  |  |  |  |  |  |  |  |
|  |  |  |  |  |  |  |  |  |  |
| Mood test (Pizza restaurants\|Food supermarkets): | | | | |  |  |  |  |  |
|  |  |  |  |  |  |  |  |  |  |
| U | 29.531 |  |  |  |  |  |  |  |  |
| Critical value | 3.841 |  |  |  |  |  |  |  |  |
| DF | 1.000 |  |  |  |  |  |  |  |  |
| p-value | < 0.0001 |  |  |  |  |  |  |  |  |
| alpha | 0.05 |  |  |  |  |  |  |  |  |
| The p-value has been computed using 10000 Monte Carlo simulations. Time elapsed: 6s. | | | | | | | | |  |
|  |  |  |  |  |  |  |  |  |  |
| Test interpretation: | |  |  |  |  |  |  |  |  |
| H0: The medians of Pizza restaurants and Food supermarkets are equal. | | | | | | |  |  |  |
| Ha: Medians of Pizza restaurants and Food supermarkets are not equal | | | | | | |  |  |  |
| As the computed p-value is lower than the significance level alpha=0.05, one should reject the null hypothesis H0, and accept the alternative hypothesis Ha. | | | | | | | | |  |
|  |  |  |  |  |  |  |  |  |  |
|  |  |  |  |  |  |  |  |  |  |
|  |  |  |  |  |  |  |  |  |  |
| 95% confidence interval on the p-value: | | | |  |  |  |  |  |  |
| ] 0.000, | 0.000 [ |  |  |  |  |  |  |  |  |
|  |  |  |  |  |  |  |  |  |  |
| Mood test (Pizza restaurants\|Retail shops (non food)): | | | | | |  |  |  |  |
|  |  |  |  |  |  |  |  |  |  |
| U | 1.360 |  |  |  |  |  |  |  |  |
| Critical value | 3.841 |  |  |  |  |  |  |  |  |
| DF | 1.000 |  |  |  |  |  |  |  |  |
| p-value | 0.241 |  |  |  |  |  |  |  |  |
| alpha | 0.05 |  |  |  |  |  |  |  |  |
| The p-value has been computed using 10000 Monte Carlo simulations. Time elapsed: 6s. | | | | | | | | |  |
|  |  |  |  |  |  |  |  |  |  |
| Test interpretation: | |  |  |  |  |  |  |  |  |
| H0: The medians of Pizza restaurants and Retail shops (non food) are equal. | | | | | | | |  |  |
| Ha: Medians of Pizza restaurants and Retail shops (non food) are not equal | | | | | | | |  |  |
| As the computed p-value is greater than the significance level alpha=0.05, one cannot reject the null hypothesis H0. | | | | | | | | |  |
|  |  |  |  |  |  |  |  |  |  |
|  |  |  |  |  |  |  |  |  |  |
|  |  |  |  |  |  |  |  |  |  |
| 95% confidence interval on the p-value: | | | |  |  |  |  |  |  |
| ] 0.233, | 0.250 [ |  |  |  |  |  |  |  |  |
|  |  |  |  |  |  |  |  |  |  |
| Mood test (Pizza restaurants\|Fast-food restaurants): | | | | | |  |  |  |  |
|  |  |  |  |  |  |  |  |  |  |
| U | 2.372 |  |  |  |  |  |  |  |  |
| Critical value | 3.841 |  |  |  |  |  |  |  |  |
| DF | 1.000 |  |  |  |  |  |  |  |  |
| p-value | 0.127 |  |  |  |  |  |  |  |  |
| alpha | 0.05 |  |  |  |  |  |  |  |  |
| The p-value has been computed using 10000 Monte Carlo simulations. Time elapsed: 6s. | | | | | | | | |  |
|  |  |  |  |  |  |  |  |  |  |
| Test interpretation: | |  |  |  |  |  |  |  |  |
| H0: The medians of Pizza restaurants and Fast-food restaurants are equal. | | | | | | | |  |  |
| Ha: Medians of Pizza restaurants and Fast-food restaurants are not equal | | | | | | | |  |  |
| As the computed p-value is greater than the significance level alpha=0.05, one cannot reject the null hypothesis H0. | | | | | | | | |  |
|  |  |  |  |  |  |  |  |  |  |
|  |  |  |  |  |  |  |  |  |  |
|  |  |  |  |  |  |  |  |  |  |
| 95% confidence interval on the p-value: | | | |  |  |  |  |  |  |
| ] 0.120, | 0.133 [ |  |  |  |  |  |  |  |  |
|  |  |  |  |  |  |  |  |  |  |
| Mood test (Pizza restaurants\|Coffee shops): | | | | |  |  |  |  |  |
|  |  |  |  |  |  |  |  |  |  |
| U | 6.806 |  |  |  |  |  |  |  |  |
| Critical value | 3.841 |  |  |  |  |  |  |  |  |
| DF | 1.000 |  |  |  |  |  |  |  |  |
| p-value | 0.010 |  |  |  |  |  |  |  |  |
| alpha | 0.05 |  |  |  |  |  |  |  |  |
| The p-value has been computed using 10000 Monte Carlo simulations. Time elapsed: 6s. | | | | | | | | |  |
|  |  |  |  |  |  |  |  |  |  |
| Test interpretation: | |  |  |  |  |  |  |  |  |
| H0: The medians of Pizza restaurants and Coffee shops are equal. | | | | | | |  |  |  |
| Ha: Medians of Pizza restaurants and Coffee shops are not equal | | | | | | |  |  |  |
| As the computed p-value is lower than the significance level alpha=0.05, one should reject the null hypothesis H0, and accept the alternative hypothesis Ha. | | | | | | | | |  |
|  |  |  |  |  |  |  |  |  |  |
|  |  |  |  |  |  |  |  |  |  |
|  |  |  |  |  |  |  |  |  |  |
| 95% confidence interval on the p-value: | | | |  |  |  |  |  |  |
| ] 0.008, | 0.012 [ |  |  |  |  |  |  |  |  |
|  |  |  |  |  |  |  |  |  |  |
| Mood test (Pizza restaurants\|Banks): | | | |  |  |  |  |  |  |
|  |  |  |  |  |  |  |  |  |  |
| U | 14.693 |  |  |  |  |  |  |  |  |
| Critical value | 3.841 |  |  |  |  |  |  |  |  |
| DF | 1.000 |  |  |  |  |  |  |  |  |
| p-value | 0.000 |  |  |  |  |  |  |  |  |
| alpha | 0.05 |  |  |  |  |  |  |  |  |
| The p-value has been computed using 10000 Monte Carlo simulations. Time elapsed: 6s. | | | | | | | | |  |
|  |  |  |  |  |  |  |  |  |  |
| Test interpretation: | |  |  |  |  |  |  |  |  |
| H0: The medians of Pizza restaurants and Banks are equal. | | | | | |  |  |  |  |
| Ha: Medians of Pizza restaurants and Banks are not equal | | | | | |  |  |  |  |
| As the computed p-value is lower than the significance level alpha=0.05, one should reject the null hypothesis H0, and accept the alternative hypothesis Ha. | | | | | | | | |  |
|  |  |  |  |  |  |  |  |  |  |
|  |  |  |  |  |  |  |  |  |  |
|  |  |  |  |  |  |  |  |  |  |
| 95% confidence interval on the p-value: | | | |  |  |  |  |  |  |
| ] 0.000, | 0.000 [ |  |  |  |  |  |  |  |  |
|  |  |  |  |  |  |  |  |  |  |
| Mood test (Pizza restaurants\|Pharmacies): | | | | |  |  |  |  |  |
|  |  |  |  |  |  |  |  |  |  |
| U | 59.557 |  |  |  |  |  |  |  |  |
| Critical value | 3.841 |  |  |  |  |  |  |  |  |
| DF | 1.000 |  |  |  |  |  |  |  |  |
| p-value | < 0.0001 |  |  |  |  |  |  |  |  |
| alpha | 0.05 |  |  |  |  |  |  |  |  |
| The p-value has been computed using 10000 Monte Carlo simulations. Time elapsed: 6s. | | | | | | | | |  |
|  |  |  |  |  |  |  |  |  |  |
| Test interpretation: | |  |  |  |  |  |  |  |  |
| H0: The medians of Pizza restaurants and Pharmacies are equal. | | | | | | |  |  |  |
| Ha: Medians of Pizza restaurants and Pharmacies are not equal | | | | | | |  |  |  |
| As the computed p-value is lower than the significance level alpha=0.05, one should reject the null hypothesis H0, and accept the alternative hypothesis Ha. | | | | | | | | |  |
|  |  |  |  |  |  |  |  |  |  |
|  |  |  |  |  |  |  |  |  |  |
|  |  |  |  |  |  |  |  |  |  |
| 95% confidence interval on the p-value: | | | |  |  |  |  |  |  |
| ] 0.000, | 0.000 [ |  |  |  |  |  |  |  |  |
|  |  |  |  |  |  |  |  |  |  |
| Mood test (Pizza restaurants\|Post Offices): | | | | |  |  |  |  |  |
|  |  |  |  |  |  |  |  |  |  |
| U | 15.086 |  |  |  |  |  |  |  |  |
| Critical value | 3.841 |  |  |  |  |  |  |  |  |
| DF | 1.000 |  |  |  |  |  |  |  |  |
| p-value | 0.000 |  |  |  |  |  |  |  |  |
| alpha | 0.05 |  |  |  |  |  |  |  |  |
| The p-value has been computed using 10000 Monte Carlo simulations. Time elapsed: 6s. | | | | | | | | |  |
|  |  |  |  |  |  |  |  |  |  |
| Test interpretation: | |  |  |  |  |  |  |  |  |
| H0: The medians of Pizza restaurants and Post Offices are equal. | | | | | | |  |  |  |
| Ha: Medians of Pizza restaurants and Post Offices are not equal | | | | | | |  |  |  |
| As the computed p-value is lower than the significance level alpha=0.05, one should reject the null hypothesis H0, and accept the alternative hypothesis Ha. | | | | | | | | |  |
|  |  |  |  |  |  |  |  |  |  |
|  |  |  |  |  |  |  |  |  |  |
|  |  |  |  |  |  |  |  |  |  |
| 95% confidence interval on the p-value: | | | |  |  |  |  |  |  |
| ] 0.000, | 0.000 [ |  |  |  |  |  |  |  |  |
|  |  |  |  |  |  |  |  |  |  |
| Mood test (Pizza restaurants\|Gas stations): | | | | |  |  |  |  |  |
|  |  |  |  |  |  |  |  |  |  |
| U | 27.066 |  |  |  |  |  |  |  |  |
| Critical value | 3.841 |  |  |  |  |  |  |  |  |
| DF | 1.000 |  |  |  |  |  |  |  |  |
| p-value | < 0.0001 |  |  |  |  |  |  |  |  |
| alpha | 0.05 |  |  |  |  |  |  |  |  |
| The p-value has been computed using 10000 Monte Carlo simulations. Time elapsed: 6s. | | | | | | | | |  |
|  |  |  |  |  |  |  |  |  |  |
| Test interpretation: | |  |  |  |  |  |  |  |  |
| H0: The medians of Pizza restaurants and Gas stations are equal. | | | | | | |  |  |  |
| Ha: Medians of Pizza restaurants and Gas stations are not equal | | | | | | |  |  |  |
| As the computed p-value is lower than the significance level alpha=0.05, one should reject the null hypothesis H0, and accept the alternative hypothesis Ha. | | | | | | | | |  |
|  |  |  |  |  |  |  |  |  |  |
|  |  |  |  |  |  |  |  |  |  |
|  |  |  |  |  |  |  |  |  |  |
| 95% confidence interval on the p-value: | | | |  |  |  |  |  |  |
| ] 0.000, | 0.000 [ |  |  |  |  |  |  |  |  |
|  |  |  |  |  |  |  |  |  |  |
| Mood test (Gyms\|Food supermarkets): | | | |  |  |  |  |  |  |
|  |  |  |  |  |  |  |  |  |  |
| U | 0.033 |  |  |  |  |  |  |  |  |
| Critical value | 3.841 |  |  |  |  |  |  |  |  |
| DF | 1.000 |  |  |  |  |  |  |  |  |
| p-value | 0.768 |  |  |  |  |  |  |  |  |
| alpha | 0.05 |  |  |  |  |  |  |  |  |
| The p-value has been computed using 10000 Monte Carlo simulations. Time elapsed: 6s. | | | | | | | | |  |
|  |  |  |  |  |  |  |  |  |  |
| Test interpretation: | |  |  |  |  |  |  |  |  |
| H0: The medians of Gyms and Food supermarkets are equal. | | | | | |  |  |  |  |
| Ha: Medians of Gyms and Food supermarkets are not equal | | | | | |  |  |  |  |
| As the computed p-value is greater than the significance level alpha=0.05, one cannot reject the null hypothesis H0. | | | | | | | | |  |
|  |  |  |  |  |  |  |  |  |  |
|  |  |  |  |  |  |  |  |  |  |
|  |  |  |  |  |  |  |  |  |  |
| 95% confidence interval on the p-value: | | | |  |  |  |  |  |  |
| ] 0.760, | 0.777 [ |  |  |  |  |  |  |  |  |
|  |  |  |  |  |  |  |  |  |  |
| Mood test (Gyms\|Retail shops (non food)): | | | | |  |  |  |  |  |
|  |  |  |  |  |  |  |  |  |  |
| U | 0.853 |  |  |  |  |  |  |  |  |
| Critical value | 3.841 |  |  |  |  |  |  |  |  |
| DF | 1.000 |  |  |  |  |  |  |  |  |
| p-value | 0.382 |  |  |  |  |  |  |  |  |
| alpha | 0.05 |  |  |  |  |  |  |  |  |
| The p-value has been computed using 10000 Monte Carlo simulations. Time elapsed: 6s. | | | | | | | | |  |
|  |  |  |  |  |  |  |  |  |  |
| Test interpretation: | |  |  |  |  |  |  |  |  |
| H0: The medians of Gyms and Retail shops (non food) are equal. | | | | | | |  |  |  |
| Ha: Medians of Gyms and Retail shops (non food) are not equal | | | | | | |  |  |  |
| As the computed p-value is greater than the significance level alpha=0.05, one cannot reject the null hypothesis H0. | | | | | | | | |  |
|  |  |  |  |  |  |  |  |  |  |
|  |  |  |  |  |  |  |  |  |  |
|  |  |  |  |  |  |  |  |  |  |
| 95% confidence interval on the p-value: | | | |  |  |  |  |  |  |
| ] 0.372, | 0.391 [ |  |  |  |  |  |  |  |  |
|  |  |  |  |  |  |  |  |  |  |
| Mood test (Gyms\|Fast-food restaurants): | | | | |  |  |  |  |  |
|  |  |  |  |  |  |  |  |  |  |
| U | 0.000 |  |  |  |  |  |  |  |  |
| Critical value | 3.841 |  |  |  |  |  |  |  |  |
| DF | 1.000 |  |  |  |  |  |  |  |  |
| p-value | 1.000 |  |  |  |  |  |  |  |  |
| alpha | 0.05 |  |  |  |  |  |  |  |  |
| The p-value has been computed using 10000 Monte Carlo simulations. Time elapsed: 6s. | | | | | | | | |  |
|  |  |  |  |  |  |  |  |  |  |
| Test interpretation: | |  |  |  |  |  |  |  |  |
| H0: The medians of Gyms and Fast-food restaurants are equal. | | | | | | |  |  |  |
| Ha: Medians of Gyms and Fast-food restaurants are not equal | | | | | |  |  |  |  |
| As the computed p-value is greater than the significance level alpha=0.05, one cannot reject the null hypothesis H0. | | | | | | | | |  |
|  |  |  |  |  |  |  |  |  |  |
|  |  |  |  |  |  |  |  |  |  |
|  |  |  |  |  |  |  |  |  |  |
| 95% confidence interval on the p-value: | | | |  |  |  |  |  |  |
| ] 1.000, | 1.000 [ |  |  |  |  |  |  |  |  |
|  |  |  |  |  |  |  |  |  |  |
| Mood test (Gyms\|Coffee shops): | | | |  |  |  |  |  |  |
|  |  |  |  |  |  |  |  |  |  |
| U | 0.000 |  |  |  |  |  |  |  |  |
| Critical value | 3.841 |  |  |  |  |  |  |  |  |
| DF | 1.000 |  |  |  |  |  |  |  |  |
| p-value | 1.000 |  |  |  |  |  |  |  |  |
| alpha | 0.05 |  |  |  |  |  |  |  |  |
| The p-value has been computed using 10000 Monte Carlo simulations. Time elapsed: 6s. | | | | | | | | |  |
|  |  |  |  |  |  |  |  |  |  |
| Test interpretation: | |  |  |  |  |  |  |  |  |
| H0: The medians of Gyms and Coffee shops are equal. | | | | | |  |  |  |  |
| Ha: Medians of Gyms and Coffee shops are not equal | | | | | |  |  |  |  |
| As the computed p-value is greater than the significance level alpha=0.05, one cannot reject the null hypothesis H0. | | | | | | | | |  |
|  |  |  |  |  |  |  |  |  |  |
|  |  |  |  |  |  |  |  |  |  |
|  |  |  |  |  |  |  |  |  |  |
| 95% confidence interval on the p-value: | | | |  |  |  |  |  |  |
| ] 1.000, | 1.000 [ |  |  |  |  |  |  |  |  |
|  |  |  |  |  |  |  |  |  |  |
| Mood test (Gyms\|Banks): | | |  |  |  |  |  |  |  |
|  |  |  |  |  |  |  |  |  |  |
| U | 2.709 |  |  |  |  |  |  |  |  |
| Critical value | 3.841 |  |  |  |  |  |  |  |  |
| DF | 1.000 |  |  |  |  |  |  |  |  |
| p-value | 0.104 |  |  |  |  |  |  |  |  |
| alpha | 0.05 |  |  |  |  |  |  |  |  |
| The p-value has been computed using 10000 Monte Carlo simulations. Time elapsed: 6s. | | | | | | | | |  |
|  |  |  |  |  |  |  |  |  |  |
| Test interpretation: | |  |  |  |  |  |  |  |  |
| H0: The medians of Gyms and Banks are equal. | | | | |  |  |  |  |  |
| Ha: Medians of Gyms and Banks are not equal | | | | |  |  |  |  |  |
| As the computed p-value is greater than the significance level alpha=0.05, one cannot reject the null hypothesis H0. | | | | | | | | |  |
|  |  |  |  |  |  |  |  |  |  |
|  |  |  |  |  |  |  |  |  |  |
|  |  |  |  |  |  |  |  |  |  |
| 95% confidence interval on the p-value: | | | |  |  |  |  |  |  |
| ] 0.098, | 0.109 [ |  |  |  |  |  |  |  |  |
|  |  |  |  |  |  |  |  |  |  |
| Mood test (Gyms\|Pharmacies): | | | |  |  |  |  |  |  |
|  |  |  |  |  |  |  |  |  |  |
| U | 26.847 |  |  |  |  |  |  |  |  |
| Critical value | 3.841 |  |  |  |  |  |  |  |  |
| DF | 1.000 |  |  |  |  |  |  |  |  |
| p-value | < 0.0001 |  |  |  |  |  |  |  |  |
| alpha | 0.05 |  |  |  |  |  |  |  |  |
| The p-value has been computed using 10000 Monte Carlo simulations. Time elapsed: 6s. | | | | | | | | |  |
|  |  |  |  |  |  |  |  |  |  |
| Test interpretation: | |  |  |  |  |  |  |  |  |
| H0: The medians of Gyms and Pharmacies are equal. | | | | | |  |  |  |  |
| Ha: Medians of Gyms and Pharmacies are not equal | | | | |  |  |  |  |  |
| As the computed p-value is lower than the significance level alpha=0.05, one should reject the null hypothesis H0, and accept the alternative hypothesis Ha. | | | | | | | | |  |
|  |  |  |  |  |  |  |  |  |  |
|  |  |  |  |  |  |  |  |  |  |
|  |  |  |  |  |  |  |  |  |  |
| 95% confidence interval on the p-value: | | | |  |  |  |  |  |  |
| ] 0.000, | 0.000 [ |  |  |  |  |  |  |  |  |
|  |  |  |  |  |  |  |  |  |  |
| Mood test (Gyms\|Post Offices): | | | |  |  |  |  |  |  |
|  |  |  |  |  |  |  |  |  |  |
| U | 0.000 |  |  |  |  |  |  |  |  |
| Critical value | 3.841 |  |  |  |  |  |  |  |  |
| DF | 1.000 |  |  |  |  |  |  |  |  |
| p-value | 1.000 |  |  |  |  |  |  |  |  |
| alpha | 0.05 |  |  |  |  |  |  |  |  |
| The p-value has been computed using 10000 Monte Carlo simulations. Time elapsed: 6s. | | | | | | | | |  |
|  |  |  |  |  |  |  |  |  |  |
| Test interpretation: | |  |  |  |  |  |  |  |  |
| H0: The medians of Gyms and Post Offices are equal. | | | | | |  |  |  |  |
| Ha: Medians of Gyms and Post Offices are not equal | | | | | |  |  |  |  |
| As the computed p-value is greater than the significance level alpha=0.05, one cannot reject the null hypothesis H0. | | | | | | | | |  |
|  |  |  |  |  |  |  |  |  |  |
|  |  |  |  |  |  |  |  |  |  |
|  |  |  |  |  |  |  |  |  |  |
| 95% confidence interval on the p-value: | | | |  |  |  |  |  |  |
| ] 1.000, | 1.000 [ |  |  |  |  |  |  |  |  |
|  |  |  |  |  |  |  |  |  |  |
| Mood test (Gyms\|Gas stations): | | | |  |  |  |  |  |  |
|  |  |  |  |  |  |  |  |  |  |
| U | 21.505 |  |  |  |  |  |  |  |  |
| Critical value | 3.841 |  |  |  |  |  |  |  |  |
| DF | 1.000 |  |  |  |  |  |  |  |  |
| p-value | < 0.0001 |  |  |  |  |  |  |  |  |
| alpha | 0.05 |  |  |  |  |  |  |  |  |
| The p-value has been computed using 10000 Monte Carlo simulations. Time elapsed: 6s. | | | | | | | | |  |
|  |  |  |  |  |  |  |  |  |  |
| Test interpretation: | |  |  |  |  |  |  |  |  |
| H0: The medians of Gyms and Gas stations are equal. | | | | | |  |  |  |  |
| Ha: Medians of Gyms and Gas stations are not equal | | | | | |  |  |  |  |
| As the computed p-value is lower than the significance level alpha=0.05, one should reject the null hypothesis H0, and accept the alternative hypothesis Ha. | | | | | | | | |  |
|  |  |  |  |  |  |  |  |  |  |
|  |  |  |  |  |  |  |  |  |  |
|  |  |  |  |  |  |  |  |  |  |
| 95% confidence interval on the p-value: | | | |  |  |  |  |  |  |
| ] 0.000, | 0.000 [ |  |  |  |  |  |  |  |  |
|  |  |  |  |  |  |  |  |  |  |
| Mood test (Food supermarkets\|Retail shops (non food)): | | | | | |  |  |  |  |
|  |  |  |  |  |  |  |  |  |  |
| U | 16.179 |  |  |  |  |  |  |  |  |
| Critical value | 3.841 |  |  |  |  |  |  |  |  |
| DF | 1.000 |  |  |  |  |  |  |  |  |
| p-value | 0.000 |  |  |  |  |  |  |  |  |
| alpha | 0.05 |  |  |  |  |  |  |  |  |
| The p-value has been computed using 10000 Monte Carlo simulations. Time elapsed: 6s. | | | | | | | | |  |
|  |  |  |  |  |  |  |  |  |  |
| Test interpretation: | |  |  |  |  |  |  |  |  |
| H0: The medians of Food supermarkets and Retail shops (non food) are equal. | | | | | | | |  |  |
| Ha: Medians of Food supermarkets and Retail shops (non food) are not equal | | | | | | | |  |  |
| As the computed p-value is lower than the significance level alpha=0.05, one should reject the null hypothesis H0, and accept the alternative hypothesis Ha. | | | | | | | | |  |
|  |  |  |  |  |  |  |  |  |  |
|  |  |  |  |  |  |  |  |  |  |
|  |  |  |  |  |  |  |  |  |  |
| 95% confidence interval on the p-value: | | | |  |  |  |  |  |  |
| ] 0.000, | 0.000 [ |  |  |  |  |  |  |  |  |
|  |  |  |  |  |  |  |  |  |  |
| Mood test (Food supermarkets\|Fast-food restaurants): | | | | | |  |  |  |  |
|  |  |  |  |  |  |  |  |  |  |
| U | 0.000 |  |  |  |  |  |  |  |  |
| Critical value | 3.841 |  |  |  |  |  |  |  |  |
| DF | 1.000 |  |  |  |  |  |  |  |  |
| p-value | 1.000 |  |  |  |  |  |  |  |  |
| alpha | 0.05 |  |  |  |  |  |  |  |  |
| The p-value has been computed using 10000 Monte Carlo simulations. Time elapsed: 6s. | | | | | | | | |  |
|  |  |  |  |  |  |  |  |  |  |
| Test interpretation: | |  |  |  |  |  |  |  |  |
| H0: The medians of Food supermarkets and Fast-food restaurants are equal. | | | | | | | |  |  |
| Ha: Medians of Food supermarkets and Fast-food restaurants are not equal | | | | | | | |  |  |
| As the computed p-value is greater than the significance level alpha=0.05, one cannot reject the null hypothesis H0. | | | | | | | | |  |
|  |  |  |  |  |  |  |  |  |  |
|  |  |  |  |  |  |  |  |  |  |
|  |  |  |  |  |  |  |  |  |  |
| 95% confidence interval on the p-value: | | | |  |  |  |  |  |  |
| ] 1.000, | 1.000 [ |  |  |  |  |  |  |  |  |
|  |  |  |  |  |  |  |  |  |  |
| Mood test (Food supermarkets\|Coffee shops): | | | | |  |  |  |  |  |
|  |  |  |  |  |  |  |  |  |  |
| U | 0.000 |  |  |  |  |  |  |  |  |
| Critical value | 3.841 |  |  |  |  |  |  |  |  |
| DF | 1.000 |  |  |  |  |  |  |  |  |
| p-value | 1.000 |  |  |  |  |  |  |  |  |
| alpha | 0.05 |  |  |  |  |  |  |  |  |
| The p-value has been computed using 10000 Monte Carlo simulations. Time elapsed: 6s. | | | | | | | | |  |
|  |  |  |  |  |  |  |  |  |  |
| Test interpretation: | |  |  |  |  |  |  |  |  |
| H0: The medians of Food supermarkets and Coffee shops are equal. | | | | | | |  |  |  |
| Ha: Medians of Food supermarkets and Coffee shops are not equal | | | | | | |  |  |  |
| As the computed p-value is greater than the significance level alpha=0.05, one cannot reject the null hypothesis H0. | | | | | | | | |  |
|  |  |  |  |  |  |  |  |  |  |
|  |  |  |  |  |  |  |  |  |  |
|  |  |  |  |  |  |  |  |  |  |
| 95% confidence interval on the p-value: | | | |  |  |  |  |  |  |
| ] 1.000, | 1.000 [ |  |  |  |  |  |  |  |  |
|  |  |  |  |  |  |  |  |  |  |
| Mood test (Food supermarkets\|Banks): | | | |  |  |  |  |  |  |
|  |  |  |  |  |  |  |  |  |  |
| U | 10.110 |  |  |  |  |  |  |  |  |
| Critical value | 3.841 |  |  |  |  |  |  |  |  |
| DF | 1.000 |  |  |  |  |  |  |  |  |
| p-value | 0.001 |  |  |  |  |  |  |  |  |
| alpha | 0.05 |  |  |  |  |  |  |  |  |
| The p-value has been computed using 10000 Monte Carlo simulations. Time elapsed: 6s. | | | | | | | | |  |
|  |  |  |  |  |  |  |  |  |  |
| Test interpretation: | |  |  |  |  |  |  |  |  |
| H0: The medians of Food supermarkets and Banks are equal. | | | | | |  |  |  |  |
| Ha: Medians of Food supermarkets and Banks are not equal | | | | | |  |  |  |  |
| As the computed p-value is lower than the significance level alpha=0.05, one should reject the null hypothesis H0, and accept the alternative hypothesis Ha. | | | | | | | | |  |
|  |  |  |  |  |  |  |  |  |  |
|  |  |  |  |  |  |  |  |  |  |
|  |  |  |  |  |  |  |  |  |  |
| 95% confidence interval on the p-value: | | | |  |  |  |  |  |  |
| ] 0.001, | 0.002 [ |  |  |  |  |  |  |  |  |
|  |  |  |  |  |  |  |  |  |  |
| Mood test (Food supermarkets\|Pharmacies): | | | | |  |  |  |  |  |
|  |  |  |  |  |  |  |  |  |  |
| U | 54.807 |  |  |  |  |  |  |  |  |
| Critical value | 3.841 |  |  |  |  |  |  |  |  |
| DF | 1.000 |  |  |  |  |  |  |  |  |
| p-value | < 0.0001 |  |  |  |  |  |  |  |  |
| alpha | 0.05 |  |  |  |  |  |  |  |  |
| The p-value has been computed using 10000 Monte Carlo simulations. Time elapsed: 6s. | | | | | | | | |  |
|  |  |  |  |  |  |  |  |  |  |
| Test interpretation: | |  |  |  |  |  |  |  |  |
| H0: The medians of Food supermarkets and Pharmacies are equal. | | | | | | |  |  |  |
| Ha: Medians of Food supermarkets and Pharmacies are not equal | | | | | | |  |  |  |
| As the computed p-value is lower than the significance level alpha=0.05, one should reject the null hypothesis H0, and accept the alternative hypothesis Ha. | | | | | | | | |  |
|  |  |  |  |  |  |  |  |  |  |
|  |  |  |  |  |  |  |  |  |  |
|  |  |  |  |  |  |  |  |  |  |
| 95% confidence interval on the p-value: | | | |  |  |  |  |  |  |
| ] 0.000, | 0.000 [ |  |  |  |  |  |  |  |  |
|  |  |  |  |  |  |  |  |  |  |
| Mood test (Food supermarkets\|Post Offices): | | | | |  |  |  |  |  |
|  |  |  |  |  |  |  |  |  |  |
| U | 0.000 |  |  |  |  |  |  |  |  |
| Critical value | 3.841 |  |  |  |  |  |  |  |  |
| DF | 1.000 |  |  |  |  |  |  |  |  |
| p-value | 1.000 |  |  |  |  |  |  |  |  |
| alpha | 0.05 |  |  |  |  |  |  |  |  |
| The p-value has been computed using 10000 Monte Carlo simulations. Time elapsed: 6s. | | | | | | | | |  |
|  |  |  |  |  |  |  |  |  |  |
| Test interpretation: | |  |  |  |  |  |  |  |  |
| H0: The medians of Food supermarkets and Post Offices are equal. | | | | | | |  |  |  |
| Ha: Medians of Food supermarkets and Post Offices are not equal | | | | | | |  |  |  |
| As the computed p-value is greater than the significance level alpha=0.05, one cannot reject the null hypothesis H0. | | | | | | | | |  |
|  |  |  |  |  |  |  |  |  |  |
|  |  |  |  |  |  |  |  |  |  |
|  |  |  |  |  |  |  |  |  |  |
| 95% confidence interval on the p-value: | | | |  |  |  |  |  |  |
| ] 1.000, | 1.000 [ |  |  |  |  |  |  |  |  |
|  |  |  |  |  |  |  |  |  |  |
| Mood test (Food supermarkets\|Gas stations): | | | | |  |  |  |  |  |
|  |  |  |  |  |  |  |  |  |  |
| U | 22.363 |  |  |  |  |  |  |  |  |
| Critical value | 3.841 |  |  |  |  |  |  |  |  |
| DF | 1.000 |  |  |  |  |  |  |  |  |
| p-value | < 0.0001 |  |  |  |  |  |  |  |  |
| alpha | 0.05 |  |  |  |  |  |  |  |  |
| The p-value has been computed using 10000 Monte Carlo simulations. Time elapsed: 6s. | | | | | | | | |  |
|  |  |  |  |  |  |  |  |  |  |
| Test interpretation: | |  |  |  |  |  |  |  |  |
| H0: The medians of Food supermarkets and Gas stations are equal. | | | | | | |  |  |  |
| Ha: Medians of Food supermarkets and Gas stations are not equal | | | | | | |  |  |  |
| As the computed p-value is lower than the significance level alpha=0.05, one should reject the null hypothesis H0, and accept the alternative hypothesis Ha. | | | | | | | | |  |
|  |  |  |  |  |  |  |  |  |  |
|  |  |  |  |  |  |  |  |  |  |
|  |  |  |  |  |  |  |  |  |  |
| 95% confidence interval on the p-value: | | | |  |  |  |  |  |  |
| ] 0.000, | 0.000 [ |  |  |  |  |  |  |  |  |
|  |  |  |  |  |  |  |  |  |  |
| Mood test (Retail shops (non food)\|Fast-food restaurants): | | | | | |  |  |  |  |
|  |  |  |  |  |  |  |  |  |  |
| U | 0.385 |  |  |  |  |  |  |  |  |
| Critical value | 3.841 |  |  |  |  |  |  |  |  |
| DF | 1.000 |  |  |  |  |  |  |  |  |
| p-value | 0.550 |  |  |  |  |  |  |  |  |
| alpha | 0.05 |  |  |  |  |  |  |  |  |
| The p-value has been computed using 10000 Monte Carlo simulations. Time elapsed: 6s. | | | | | | | | |  |
|  |  |  |  |  |  |  |  |  |  |
| Test interpretation: | |  |  |  |  |  |  |  |  |
| H0: The medians of Retail shops (non food) and Fast-food restaurants are equal. | | | | | | | |  |  |
| Ha: Medians of Retail shops (non food) and Fast-food restaurants are not equal | | | | | | | |  |  |
| As the computed p-value is greater than the significance level alpha=0.05, one cannot reject the null hypothesis H0. | | | | | | | | |  |
|  |  |  |  |  |  |  |  |  |  |
|  |  |  |  |  |  |  |  |  |  |
|  |  |  |  |  |  |  |  |  |  |
| 95% confidence interval on the p-value: | | | |  |  |  |  |  |  |
| ] 0.541, | 0.560 [ |  |  |  |  |  |  |  |  |
|  |  |  |  |  |  |  |  |  |  |
| Mood test (Retail shops (non food)\|Coffee shops): | | | | |  |  |  |  |  |
|  |  |  |  |  |  |  |  |  |  |
| U | 2.363 |  |  |  |  |  |  |  |  |
| Critical value | 3.841 |  |  |  |  |  |  |  |  |
| DF | 1.000 |  |  |  |  |  |  |  |  |
| p-value | 0.136 |  |  |  |  |  |  |  |  |
| alpha | 0.05 |  |  |  |  |  |  |  |  |
| The p-value has been computed using 10000 Monte Carlo simulations. Time elapsed: 6s. | | | | | | | | |  |
|  |  |  |  |  |  |  |  |  |  |
| Test interpretation: | |  |  |  |  |  |  |  |  |
| H0: The medians of Retail shops (non food) and Coffee shops are equal. | | | | | | |  |  |  |
| Ha: Medians of Retail shops (non food) and Coffee shops are not equal | | | | | | |  |  |  |
| As the computed p-value is greater than the significance level alpha=0.05, one cannot reject the null hypothesis H0. | | | | | | | | |  |
|  |  |  |  |  |  |  |  |  |  |
|  |  |  |  |  |  |  |  |  |  |
|  |  |  |  |  |  |  |  |  |  |
| 95% confidence interval on the p-value: | | | |  |  |  |  |  |  |
| ] 0.129, | 0.143 [ |  |  |  |  |  |  |  |  |
|  |  |  |  |  |  |  |  |  |  |
| Mood test (Retail shops (non food)\|Banks): | | | | |  |  |  |  |  |
|  |  |  |  |  |  |  |  |  |  |
| U | 14.819 |  |  |  |  |  |  |  |  |
| Critical value | 3.841 |  |  |  |  |  |  |  |  |
| DF | 1.000 |  |  |  |  |  |  |  |  |
| p-value | < 0.0001 |  |  |  |  |  |  |  |  |
| alpha | 0.05 |  |  |  |  |  |  |  |  |
| The p-value has been computed using 10000 Monte Carlo simulations. Time elapsed: 6s. | | | | | | | | |  |
|  |  |  |  |  |  |  |  |  |  |
| Test interpretation: | |  |  |  |  |  |  |  |  |
| H0: The medians of Retail shops (non food) and Banks are equal. | | | | | | |  |  |  |
| Ha: Medians of Retail shops (non food) and Banks are not equal | | | | | | |  |  |  |
| As the computed p-value is lower than the significance level alpha=0.05, one should reject the null hypothesis H0, and accept the alternative hypothesis Ha. | | | | | | | | |  |
|  |  |  |  |  |  |  |  |  |  |
|  |  |  |  |  |  |  |  |  |  |
|  |  |  |  |  |  |  |  |  |  |
| 95% confidence interval on the p-value: | | | |  |  |  |  |  |  |
| ] 0.000, | 0.000 [ |  |  |  |  |  |  |  |  |
|  |  |  |  |  |  |  |  |  |  |
| Mood test (Retail shops (non food)\|Pharmacies): | | | | |  |  |  |  |  |
|  |  |  |  |  |  |  |  |  |  |
| U | 74.204 |  |  |  |  |  |  |  |  |
| Critical value | 3.841 |  |  |  |  |  |  |  |  |
| DF | 1.000 |  |  |  |  |  |  |  |  |
| p-value | < 0.0001 |  |  |  |  |  |  |  |  |
| alpha | 0.05 |  |  |  |  |  |  |  |  |
| The p-value has been computed using 10000 Monte Carlo simulations. Time elapsed: 6s. | | | | | | | | |  |
|  |  |  |  |  |  |  |  |  |  |
| Test interpretation: | |  |  |  |  |  |  |  |  |
| H0: The medians of Retail shops (non food) and Pharmacies are equal. | | | | | | |  |  |  |
| Ha: Medians of Retail shops (non food) and Pharmacies are not equal | | | | | | |  |  |  |
| As the computed p-value is lower than the significance level alpha=0.05, one should reject the null hypothesis H0, and accept the alternative hypothesis Ha. | | | | | | | | |  |
|  |  |  |  |  |  |  |  |  |  |
|  |  |  |  |  |  |  |  |  |  |
|  |  |  |  |  |  |  |  |  |  |
| 95% confidence interval on the p-value: | | | |  |  |  |  |  |  |
| ] 0.000, | 0.000 [ |  |  |  |  |  |  |  |  |
|  |  |  |  |  |  |  |  |  |  |
| Mood test (Retail shops (non food)\|Post Offices): | | | | |  |  |  |  |  |
|  |  |  |  |  |  |  |  |  |  |
| U | 7.992 |  |  |  |  |  |  |  |  |
| Critical value | 3.841 |  |  |  |  |  |  |  |  |
| DF | 1.000 |  |  |  |  |  |  |  |  |
| p-value | 0.004 |  |  |  |  |  |  |  |  |
| alpha | 0.05 |  |  |  |  |  |  |  |  |
| The p-value has been computed using 10000 Monte Carlo simulations. Time elapsed: 6s. | | | | | | | | |  |
|  |  |  |  |  |  |  |  |  |  |
| Test interpretation: | |  |  |  |  |  |  |  |  |
| H0: The medians of Retail shops (non food) and Post Offices are equal. | | | | | | |  |  |  |
| Ha: Medians of Retail shops (non food) and Post Offices are not equal | | | | | | |  |  |  |
| As the computed p-value is lower than the significance level alpha=0.05, one should reject the null hypothesis H0, and accept the alternative hypothesis Ha. | | | | | | | | |  |
|  |  |  |  |  |  |  |  |  |  |
|  |  |  |  |  |  |  |  |  |  |
|  |  |  |  |  |  |  |  |  |  |
| 95% confidence interval on the p-value: | | | |  |  |  |  |  |  |
| ] 0.003, | 0.006 [ |  |  |  |  |  |  |  |  |
|  |  |  |  |  |  |  |  |  |  |
| Mood test (Retail shops (non food)\|Gas stations): | | | | |  |  |  |  |  |
|  |  |  |  |  |  |  |  |  |  |
| U | 12.408 |  |  |  |  |  |  |  |  |
| Critical value | 3.841 |  |  |  |  |  |  |  |  |
| DF | 1.000 |  |  |  |  |  |  |  |  |
| p-value | 0.000 |  |  |  |  |  |  |  |  |
| alpha | 0.05 |  |  |  |  |  |  |  |  |
| The p-value has been computed using 10000 Monte Carlo simulations. Time elapsed: 6s. | | | | | | | | |  |
|  |  |  |  |  |  |  |  |  |  |
| Test interpretation: | |  |  |  |  |  |  |  |  |
| H0: The medians of Retail shops (non food) and Gas stations are equal. | | | | | | |  |  |  |
| Ha: Medians of Retail shops (non food) and Gas stations are not equal | | | | | | |  |  |  |
| As the computed p-value is lower than the significance level alpha=0.05, one should reject the null hypothesis H0, and accept the alternative hypothesis Ha. | | | | | | | | |  |
|  |  |  |  |  |  |  |  |  |  |
|  |  |  |  |  |  |  |  |  |  |
|  |  |  |  |  |  |  |  |  |  |
| 95% confidence interval on the p-value: | | | |  |  |  |  |  |  |
| ] 0.000, | 0.001 [ |  |  |  |  |  |  |  |  |
|  |  |  |  |  |  |  |  |  |  |
| Mood test (Fast-food restaurants\|Coffee shops): | | | | |  |  |  |  |  |
|  |  |  |  |  |  |  |  |  |  |
| U | 0.026 |  |  |  |  |  |  |  |  |
| Critical value | 3.841 |  |  |  |  |  |  |  |  |
| DF | 1.000 |  |  |  |  |  |  |  |  |
| p-value | 0.874 |  |  |  |  |  |  |  |  |
| alpha | 0.05 |  |  |  |  |  |  |  |  |
| The p-value has been computed using 10000 Monte Carlo simulations. Time elapsed: 6s. | | | | | | | | |  |
|  |  |  |  |  |  |  |  |  |  |
| Test interpretation: | |  |  |  |  |  |  |  |  |
| H0: The medians of Fast-food restaurants and Coffee shops are equal. | | | | | | |  |  |  |
| Ha: Medians of Fast-food restaurants and Coffee shops are not equal | | | | | | |  |  |  |
| As the computed p-value is greater than the significance level alpha=0.05, one cannot reject the null hypothesis H0. | | | | | | | | |  |
|  |  |  |  |  |  |  |  |  |  |
|  |  |  |  |  |  |  |  |  |  |
|  |  |  |  |  |  |  |  |  |  |
| 95% confidence interval on the p-value: | | | |  |  |  |  |  |  |
| ] 0.867, | 0.880 [ |  |  |  |  |  |  |  |  |
|  |  |  |  |  |  |  |  |  |  |
| Mood test (Fast-food restaurants\|Banks): | | | | |  |  |  |  |  |
|  |  |  |  |  |  |  |  |  |  |
| U | 4.083 |  |  |  |  |  |  |  |  |
| Critical value | 3.841 |  |  |  |  |  |  |  |  |
| DF | 1.000 |  |  |  |  |  |  |  |  |
| p-value | 0.046 |  |  |  |  |  |  |  |  |
| alpha | 0.05 |  |  |  |  |  |  |  |  |
| The p-value has been computed using 10000 Monte Carlo simulations. Time elapsed: 6s. | | | | | | | | |  |
|  |  |  |  |  |  |  |  |  |  |
| Test interpretation: | |  |  |  |  |  |  |  |  |
| H0: The medians of Fast-food restaurants and Banks are equal. | | | | | | |  |  |  |
| Ha: Medians of Fast-food restaurants and Banks are not equal | | | | | |  |  |  |  |
| As the computed p-value is lower than the significance level alpha=0.05, one should reject the null hypothesis H0, and accept the alternative hypothesis Ha. | | | | | | | | |  |
|  |  |  |  |  |  |  |  |  |  |
|  |  |  |  |  |  |  |  |  |  |
|  |  |  |  |  |  |  |  |  |  |
| 95% confidence interval on the p-value: | | | |  |  |  |  |  |  |
| ] 0.042, | 0.050 [ |  |  |  |  |  |  |  |  |
|  |  |  |  |  |  |  |  |  |  |
| Mood test (Fast-food restaurants\|Pharmacies): | | | | |  |  |  |  |  |
|  |  |  |  |  |  |  |  |  |  |
| U | 33.236 |  |  |  |  |  |  |  |  |
| Critical value | 3.841 |  |  |  |  |  |  |  |  |
| DF | 1.000 |  |  |  |  |  |  |  |  |
| p-value | < 0.0001 |  |  |  |  |  |  |  |  |
| alpha | 0.05 |  |  |  |  |  |  |  |  |
| The p-value has been computed using 10000 Monte Carlo simulations. Time elapsed: 6s. | | | | | | | | |  |
|  |  |  |  |  |  |  |  |  |  |
| Test interpretation: | |  |  |  |  |  |  |  |  |
| H0: The medians of Fast-food restaurants and Pharmacies are equal. | | | | | | |  |  |  |
| Ha: Medians of Fast-food restaurants and Pharmacies are not equal | | | | | | |  |  |  |
| As the computed p-value is lower than the significance level alpha=0.05, one should reject the null hypothesis H0, and accept the alternative hypothesis Ha. | | | | | | | | |  |
|  |  |  |  |  |  |  |  |  |  |
|  |  |  |  |  |  |  |  |  |  |
|  |  |  |  |  |  |  |  |  |  |
| 95% confidence interval on the p-value: | | | |  |  |  |  |  |  |
| ] 0.000, | 0.000 [ |  |  |  |  |  |  |  |  |
|  |  |  |  |  |  |  |  |  |  |
| Mood test (Fast-food restaurants\|Post Offices): | | | | |  |  |  |  |  |
|  |  |  |  |  |  |  |  |  |  |
| U | 0.020 |  |  |  |  |  |  |  |  |
| Critical value | 3.841 |  |  |  |  |  |  |  |  |
| DF | 1.000 |  |  |  |  |  |  |  |  |
| p-value | 0.904 |  |  |  |  |  |  |  |  |
| alpha | 0.05 |  |  |  |  |  |  |  |  |
| The p-value has been computed using 10000 Monte Carlo simulations. Time elapsed: 6s. | | | | | | | | |  |
|  |  |  |  |  |  |  |  |  |  |
| Test interpretation: | |  |  |  |  |  |  |  |  |
| H0: The medians of Fast-food restaurants and Post Offices are equal. | | | | | | |  |  |  |
| Ha: Medians of Fast-food restaurants and Post Offices are not equal | | | | | | |  |  |  |
| As the computed p-value is greater than the significance level alpha=0.05, one cannot reject the null hypothesis H0. | | | | | | | | |  |
|  |  |  |  |  |  |  |  |  |  |
|  |  |  |  |  |  |  |  |  |  |
|  |  |  |  |  |  |  |  |  |  |
| 95% confidence interval on the p-value: | | | |  |  |  |  |  |  |
| ] 0.899, | 0.910 [ |  |  |  |  |  |  |  |  |
|  |  |  |  |  |  |  |  |  |  |
| Mood test (Fast-food restaurants\|Gas stations): | | | | |  |  |  |  |  |
|  |  |  |  |  |  |  |  |  |  |
| U | 33.539 |  |  |  |  |  |  |  |  |
| Critical value | 3.841 |  |  |  |  |  |  |  |  |
| DF | 1.000 |  |  |  |  |  |  |  |  |
| p-value | < 0.0001 |  |  |  |  |  |  |  |  |
| alpha | 0.05 |  |  |  |  |  |  |  |  |
| The p-value has been computed using 10000 Monte Carlo simulations. Time elapsed: 6s. | | | | | | | | |  |
|  |  |  |  |  |  |  |  |  |  |
| Test interpretation: | |  |  |  |  |  |  |  |  |
| H0: The medians of Fast-food restaurants and Gas stations are equal. | | | | | | |  |  |  |
| Ha: Medians of Fast-food restaurants and Gas stations are not equal | | | | | | |  |  |  |
| As the computed p-value is lower than the significance level alpha=0.05, one should reject the null hypothesis H0, and accept the alternative hypothesis Ha. | | | | | | | | |  |
|  |  |  |  |  |  |  |  |  |  |
|  |  |  |  |  |  |  |  |  |  |
|  |  |  |  |  |  |  |  |  |  |
| 95% confidence interval on the p-value: | | | |  |  |  |  |  |  |
| ] 0.000, | 0.000 [ |  |  |  |  |  |  |  |  |
|  |  |  |  |  |  |  |  |  |  |
| Mood test (Coffee shops\|Banks): | | | |  |  |  |  |  |  |
|  |  |  |  |  |  |  |  |  |  |
| U | 6.028 |  |  |  |  |  |  |  |  |
| Critical value | 3.841 |  |  |  |  |  |  |  |  |
| DF | 1.000 |  |  |  |  |  |  |  |  |
| p-value | 0.015 |  |  |  |  |  |  |  |  |
| alpha | 0.05 |  |  |  |  |  |  |  |  |
| The p-value has been computed using 10000 Monte Carlo simulations. Time elapsed: 6s. | | | | | | | | |  |
|  |  |  |  |  |  |  |  |  |  |
| Test interpretation: | |  |  |  |  |  |  |  |  |
| H0: The medians of Coffee shops and Banks are equal. | | | | | |  |  |  |  |
| Ha: Medians of Coffee shops and Banks are not equal | | | | | |  |  |  |  |
| As the computed p-value is lower than the significance level alpha=0.05, one should reject the null hypothesis H0, and accept the alternative hypothesis Ha. | | | | | | | | |  |
|  |  |  |  |  |  |  |  |  |  |
|  |  |  |  |  |  |  |  |  |  |
|  |  |  |  |  |  |  |  |  |  |
| 95% confidence interval on the p-value: | | | |  |  |  |  |  |  |
| ] 0.012, | 0.017 [ |  |  |  |  |  |  |  |  |
|  |  |  |  |  |  |  |  |  |  |
| Mood test (Coffee shops\|Pharmacies): | | | |  |  |  |  |  |  |
|  |  |  |  |  |  |  |  |  |  |
| U | 39.707 |  |  |  |  |  |  |  |  |
| Critical value | 3.841 |  |  |  |  |  |  |  |  |
| DF | 1.000 |  |  |  |  |  |  |  |  |
| p-value | < 0.0001 |  |  |  |  |  |  |  |  |
| alpha | 0.05 |  |  |  |  |  |  |  |  |
| The p-value has been computed using 10000 Monte Carlo simulations. Time elapsed: 6s. | | | | | | | | |  |
|  |  |  |  |  |  |  |  |  |  |
| Test interpretation: | |  |  |  |  |  |  |  |  |
| H0: The medians of Coffee shops and Pharmacies are equal. | | | | | |  |  |  |  |
| Ha: Medians of Coffee shops and Pharmacies are not equal | | | | | |  |  |  |  |
| As the computed p-value is lower than the significance level alpha=0.05, one should reject the null hypothesis H0, and accept the alternative hypothesis Ha. | | | | | | | | |  |
|  |  |  |  |  |  |  |  |  |  |
|  |  |  |  |  |  |  |  |  |  |
|  |  |  |  |  |  |  |  |  |  |
| 95% confidence interval on the p-value: | | | |  |  |  |  |  |  |
| ] 0.000, | 0.000 [ |  |  |  |  |  |  |  |  |
|  |  |  |  |  |  |  |  |  |  |
| Mood test (Coffee shops\|Post Offices): | | | |  |  |  |  |  |  |
|  |  |  |  |  |  |  |  |  |  |
| U | 0.028 |  |  |  |  |  |  |  |  |
| Critical value | 3.841 |  |  |  |  |  |  |  |  |
| DF | 1.000 |  |  |  |  |  |  |  |  |
| p-value | 0.857 |  |  |  |  |  |  |  |  |
| alpha | 0.05 |  |  |  |  |  |  |  |  |
| The p-value has been computed using 10000 Monte Carlo simulations. Time elapsed: 6s. | | | | | | | | |  |
|  |  |  |  |  |  |  |  |  |  |
| Test interpretation: | |  |  |  |  |  |  |  |  |
| H0: The medians of Coffee shops and Post Offices are equal. | | | | | |  |  |  |  |
| Ha: Medians of Coffee shops and Post Offices are not equal | | | | | |  |  |  |  |
| As the computed p-value is greater than the significance level alpha=0.05, one cannot reject the null hypothesis H0. | | | | | | | | |  |
|  |  |  |  |  |  |  |  |  |  |
|  |  |  |  |  |  |  |  |  |  |
|  |  |  |  |  |  |  |  |  |  |
| 95% confidence interval on the p-value: | | | |  |  |  |  |  |  |
| ] 0.850, | 0.864 [ |  |  |  |  |  |  |  |  |
|  |  |  |  |  |  |  |  |  |  |
| Mood test (Coffee shops\|Gas stations): | | | |  |  |  |  |  |  |
|  |  |  |  |  |  |  |  |  |  |
| U | 17.601 |  |  |  |  |  |  |  |  |
| Critical value | 3.841 |  |  |  |  |  |  |  |  |
| DF | 1.000 |  |  |  |  |  |  |  |  |
| p-value | 0.000 |  |  |  |  |  |  |  |  |
| alpha | 0.05 |  |  |  |  |  |  |  |  |
| The p-value has been computed using 10000 Monte Carlo simulations. Time elapsed: 6s. | | | | | | | | |  |
|  |  |  |  |  |  |  |  |  |  |
| Test interpretation: | |  |  |  |  |  |  |  |  |
| H0: The medians of Coffee shops and Gas stations are equal. | | | | | |  |  |  |  |
| Ha: Medians of Coffee shops and Gas stations are not equal | | | | | |  |  |  |  |
| As the computed p-value is lower than the significance level alpha=0.05, one should reject the null hypothesis H0, and accept the alternative hypothesis Ha. | | | | | | | | |  |
|  |  |  |  |  |  |  |  |  |  |
|  |  |  |  |  |  |  |  |  |  |
|  |  |  |  |  |  |  |  |  |  |
| 95% confidence interval on the p-value: | | | |  |  |  |  |  |  |
| ] 0.000, | 0.000 [ |  |  |  |  |  |  |  |  |
|  |  |  |  |  |  |  |  |  |  |
| Mood test (Banks\|Pharmacies): | | | |  |  |  |  |  |  |
|  |  |  |  |  |  |  |  |  |  |
| U | 12.844 |  |  |  |  |  |  |  |  |
| Critical value | 3.841 |  |  |  |  |  |  |  |  |
| DF | 1.000 |  |  |  |  |  |  |  |  |
| p-value | 0.000 |  |  |  |  |  |  |  |  |
| alpha | 0.05 |  |  |  |  |  |  |  |  |
| The p-value has been computed using 10000 Monte Carlo simulations. Time elapsed: 6s. | | | | | | | | |  |
|  |  |  |  |  |  |  |  |  |  |
| Test interpretation: | |  |  |  |  |  |  |  |  |
| H0: The medians of Banks and Pharmacies are equal. | | | | | |  |  |  |  |
| Ha: Medians of Banks and Pharmacies are not equal | | | | | |  |  |  |  |
| As the computed p-value is lower than the significance level alpha=0.05, one should reject the null hypothesis H0, and accept the alternative hypothesis Ha. | | | | | | | | |  |
|  |  |  |  |  |  |  |  |  |  |
|  |  |  |  |  |  |  |  |  |  |
|  |  |  |  |  |  |  |  |  |  |
| 95% confidence interval on the p-value: | | | |  |  |  |  |  |  |
| ] 0.000, | 0.000 [ |  |  |  |  |  |  |  |  |
|  |  |  |  |  |  |  |  |  |  |
| Mood test (Banks\|Post Offices): | | | |  |  |  |  |  |  |
|  |  |  |  |  |  |  |  |  |  |
| U | 6.585 |  |  |  |  |  |  |  |  |
| Critical value | 3.841 |  |  |  |  |  |  |  |  |
| DF | 1.000 |  |  |  |  |  |  |  |  |
| p-value | 0.011 |  |  |  |  |  |  |  |  |
| alpha | 0.05 |  |  |  |  |  |  |  |  |
| The p-value has been computed using 10000 Monte Carlo simulations. Time elapsed: 6s. | | | | | | | | |  |
|  |  |  |  |  |  |  |  |  |  |
| Test interpretation: | |  |  |  |  |  |  |  |  |
| H0: The medians of Banks and Post Offices are equal. | | | | | |  |  |  |  |
| Ha: Medians of Banks and Post Offices are not equal | | | | | |  |  |  |  |
| As the computed p-value is lower than the significance level alpha=0.05, one should reject the null hypothesis H0, and accept the alternative hypothesis Ha. | | | | | | | | |  |
|  |  |  |  |  |  |  |  |  |  |
|  |  |  |  |  |  |  |  |  |  |
|  |  |  |  |  |  |  |  |  |  |
| 95% confidence interval on the p-value: | | | |  |  |  |  |  |  |
| ] 0.009, | 0.013 [ |  |  |  |  |  |  |  |  |
|  |  |  |  |  |  |  |  |  |  |
| Mood test (Banks\|Gas stations): | | | |  |  |  |  |  |  |
|  |  |  |  |  |  |  |  |  |  |
| U | 5.960 |  |  |  |  |  |  |  |  |
| Critical value | 3.841 |  |  |  |  |  |  |  |  |
| DF | 1.000 |  |  |  |  |  |  |  |  |
| p-value | 0.016 |  |  |  |  |  |  |  |  |
| alpha | 0.05 |  |  |  |  |  |  |  |  |
| The p-value has been computed using 10000 Monte Carlo simulations. Time elapsed: 6s. | | | | | | | | |  |
|  |  |  |  |  |  |  |  |  |  |
| Test interpretation: | |  |  |  |  |  |  |  |  |
| H0: The medians of Banks and Gas stations are equal. | | | | | |  |  |  |  |
| Ha: Medians of Banks and Gas stations are not equal | | | | | |  |  |  |  |
| As the computed p-value is lower than the significance level alpha=0.05, one should reject the null hypothesis H0, and accept the alternative hypothesis Ha. | | | | | | | | |  |
|  |  |  |  |  |  |  |  |  |  |
|  |  |  |  |  |  |  |  |  |  |
|  |  |  |  |  |  |  |  |  |  |
| 95% confidence interval on the p-value: | | | |  |  |  |  |  |  |
| ] 0.014, | 0.019 [ |  |  |  |  |  |  |  |  |
|  |  |  |  |  |  |  |  |  |  |
| Mood test (Pharmacies\|Post Offices): | | | |  |  |  |  |  |  |
|  |  |  |  |  |  |  |  |  |  |
| U | 41.561 |  |  |  |  |  |  |  |  |
| Critical value | 3.841 |  |  |  |  |  |  |  |  |
| DF | 1.000 |  |  |  |  |  |  |  |  |
| p-value | < 0.0001 |  |  |  |  |  |  |  |  |
| alpha | 0.05 |  |  |  |  |  |  |  |  |
| The p-value has been computed using 10000 Monte Carlo simulations. Time elapsed: 6s. | | | | | | | | |  |
|  |  |  |  |  |  |  |  |  |  |
| Test interpretation: | |  |  |  |  |  |  |  |  |
| H0: The medians of Pharmacies and Post Offices are equal. | | | | | |  |  |  |  |
| Ha: Medians of Pharmacies and Post Offices are not equal | | | | | |  |  |  |  |
| As the computed p-value is lower than the significance level alpha=0.05, one should reject the null hypothesis H0, and accept the alternative hypothesis Ha. | | | | | | | | |  |
|  |  |  |  |  |  |  |  |  |  |
|  |  |  |  |  |  |  |  |  |  |
|  |  |  |  |  |  |  |  |  |  |
| 95% confidence interval on the p-value: | | | |  |  |  |  |  |  |
| ] 0.000, | 0.000 [ |  |  |  |  |  |  |  |  |
|  |  |  |  |  |  |  |  |  |  |
| Mood test (Pharmacies\|Gas stations): | | | |  |  |  |  |  |  |
|  |  |  |  |  |  |  |  |  |  |
| U | 0.000 |  |  |  |  |  |  |  |  |
| Critical value | 3.841 |  |  |  |  |  |  |  |  |
| DF | 1.000 |  |  |  |  |  |  |  |  |
| p-value | 0.890 |  |  |  |  |  |  |  |  |
| alpha | 0.05 |  |  |  |  |  |  |  |  |
| The p-value has been computed using 10000 Monte Carlo simulations. Time elapsed: 6s. | | | | | | | | |  |
|  |  |  |  |  |  |  |  |  |  |
| Test interpretation: | |  |  |  |  |  |  |  |  |
| H0: The medians of Pharmacies and Gas stations are equal. | | | | | |  |  |  |  |
| Ha: Medians of Pharmacies and Gas stations are not equal | | | | | |  |  |  |  |
| As the computed p-value is greater than the significance level alpha=0.05, one cannot reject the null hypothesis H0. | | | | | | | | |  |
|  |  |  |  |  |  |  |  |  |  |
|  |  |  |  |  |  |  |  |  |  |
|  |  |  |  |  |  |  |  |  |  |
| 95% confidence interval on the p-value: | | | |  |  |  |  |  |  |
| ] 0.884, | 0.896 [ |  |  |  |  |  |  |  |  |
|  |  |  |  |  |  |  |  |  |  |
| Mood test (Post Offices\|Gas stations): | | | |  |  |  |  |  |  |
|  |  |  |  |  |  |  |  |  |  |
| U | 18.269 |  |  |  |  |  |  |  |  |
| Critical value | 3.841 |  |  |  |  |  |  |  |  |
| DF | 1.000 |  |  |  |  |  |  |  |  |
| p-value | 0.000 |  |  |  |  |  |  |  |  |
| alpha | 0.05 |  |  |  |  |  |  |  |  |
| The p-value has been computed using 10000 Monte Carlo simulations. Time elapsed: 6s. | | | | | | | | |  |
|  |  |  |  |  |  |  |  |  |  |
| Test interpretation: | |  |  |  |  |  |  |  |  |
| H0: The medians of Post Offices and Gas stations are equal. | | | | | |  |  |  |  |
| Ha: Medians of Post Offices and Gas stations are not equal | | | | | |  |  |  |  |
| As the computed p-value is lower than the significance level alpha=0.05, one should reject the null hypothesis H0, and accept the alternative hypothesis Ha. | | | | | | | | |  |
|  |  |  |  |  |  |  |  |  |  |
|  |  |  |  |  |  |  |  |  |  |
|  |  |  |  |  |  |  |  |  |  |
| 95% confidence interval on the p-value: | | | |  |  |  |  |  |  |
| ] 0.000, | 0.000 [ |  |  |  |  |  |  |  |  |

## Model validity (least squares regression)

| **Dependent Y** | REGR_Pred1 |  |  |  |  |
| --- | --- | --- | --- | --- | --- |
| **Independent X** | Risk_of_exposure |  |  |  |  |
|  | Risk of exposure |  |  |  |  |
|  |  |  |  |  |  |
| **Least squares regression** |  |  |  |  |  |
| **Sample size** | 14 |  |  |  |  |
| **Coefficient of determination R^2^** | 0.8696 |  |  |  |  |
| **Residual standard deviation** | 65.1124 |  |  |  |  |
|  |  |  |  |  |  |
| **Regression Equation** |  |  |  |  |  |
| y = 24.8191 + 0.8696 x | | | | | |
| **Parameter** | **Coefficient** | **Std. Error** | **95% CI** | **t** | **P** |
| **Intercept** | 24.8191 | 25.4024 | -30.5280 to 80.1661 | 0.9770 | 0.3478 |
| **Slope** | 0.8696 | 0.0972 | 0.6579 to 1.0814 | 8.9474 | <0.0001 |
|  |  |  |  |  |  |
| **Analysis of Variance** |  |  |  |  |  |
| **Source** | **DF** | **Sum of Squares** | **Mean Square** |  |  |
| **Regression** | 1 | 339405.267 | 339405.2665 |  |  |
| **Residual** | 12 | 50875.4827 | 4239.62356 |  |  |
|  |  |  |  |  |  |
| **F-ratio** | 80.05552 |  |  |  |  |
| **Significance level** | P<0.0001 |  |  |  |  |
|  |  |  |  |  |  |
| **Residuals** |  |  |  |  |  |
| **Kolmogorov-Smirnov test^a^** | D=0.2252 |  |  |  |  |
| **for Normal distribution** | accept Normality (P=0.0526) |  |  |  |  |
|  |  |  |  |  |  |

## Absolute risk of exposure

### Kruskal-Wallis test

| Summary statistics: | |  |  |  |  |  |  |  |  |
| --- | --- | --- | --- | --- | --- | --- | --- | --- | --- |
|  |  |  |  |  |  |  |  |  |  |
| Variable | Observations | Obs. with missing data | Obs. without missing data | Minimum | Maximum | Mean | Std. deviation |  |  |
| Delta | 14 | 0 | 14 | 31.200 | 730.080 | 190.394 | 185.800 |  |  |
| Ancestral | 14 | 0 | 14 | 1.390 | 27.460 | 9.481 | 10.254 |  |  |
|  |  |  |  |  |  |  |  |  |  |
|  |  |  |  |  |  |  |  |  |  |
| Kruskal-Wallis test / Two-tailed test: | | | |  |  |  |  |  |  |
|  |  |  |  |  |  |  |  |  |  |
| K | 20.382 |  |  |  |  |  |  |  |  |
| p-value (one-tailed) | **<0.0001** |  |  |  |  |  |  |  |  |
| alpha | 0.050 |  |  |  |  |  |  |  |  |
| The p-value has been computed using 10000 Monte Carlo simulations. Time elapsed: 0s. | | | | | | | | |  |
| 99% confidence interval on the p-value: | | | |  |  |  |  |  |  |
| ] 0.000, | 0.000 [ |  |  |  |  |  |  |  |  |
|  |  |  |  |  |  |  |  |  |  |
| Test interpretation: | |  |  |  |  |  |  |  |  |
| H0: The samples come from the same population. | | | | |  |  |  |  |  |
| Ha: The samples do not come from the same population. | | | | | |  |  |  |  |
| As the computed p-value is lower than the significance level alpha=0.05, one should reject the null hypothesis H0, and accept the alternative hypothesis Ha. | | | | | | | | |  |
|  |  |  |  |  |  |  |  |  |  |
|  |  |  |  |  |  |  |  |  |  |
|  |  |  |  |  |  |  |  |  |  |
| Ties have been detected in the data and the appropriate corrections have been applied. | | | | | | | | |  |
|  |  |  |  |  |  |  |  |  |  |
|  |  |  |  |  |  |  |  |  |  |
| Multiple pairwise comparisons using Dunn's procedure / Two-tailed test: | | | | | | | |  |  |
|  |  |  |  |  |  |  |  |  |  |
| Sample | Frequency | Sum of ranks | Mean of ranks | Groups | |  |  |  |  |
| Ancestral | 14 | 105.000 | 7.500 | A |  |  |  |  |  |
| Delta | 14 | 301.000 | 21.500 |  | B |  |  |  |  |
|  |  |  |  |  |  |  |  |  |  |
|  |  |  |  |  |  |  |  |  |  |
| \|  \| \| --- \| |  |  |  |  |  |  |  |  |  |
|  |  |  |  |  |  |  |  |  |  |
|  |  |  |  |  |  |  |  |  |  |
|  |  |  |  |  |  |  |  |  |  |
|  |  |  |  |  |  |  |  |  |  |
|  |  |  |  |  |  |  |  |  |  |
|  |  |  |  |  |  |  |  |  |  |
|  |  |  |  |  |  |  |  |  |  |
|  |  |  |  |  |  |  |  |  |  |
|  |  |  |  |  |  |  |  |  |  |
|  |  |  |  |  |  |  |  |  |  |
|  |  |  |  |  |  |  |  |  |  |
|  |  |  |  |  |  |  |  |  |  |
|  |  |  |  |  |  |  |  |  |  |
|  |  |  |  |  |  |  |  |  |  |
|  |  |  |  |  |  |  |  |  |  |
|  |  |  |  |  |  |  |  |  |  |
|  |  |  |  |  |  |  |  |  |  |
|  |  |  |  |  |  |  |  |  |  |
| \|  \| \| --- \| |  |  |  |  |  |  |  |  |  |
|  |  |  |  |  |  |  |  |  |  |
|  |  |  |  |  |  |  |  |  |  |
|  |  |  |  |  |  |  |  |  |  |
|  |  |  |  |  |  |  |  |  |  |
|  |  |  |  |  |  |  |  |  |  |
|  |  |  |  |  |  |  |  |  |  |
|  |  |  |  |  |  |  |  |  |  |
|  |  |  |  |  |  |  |  |  |  |
|  |  |  |  |  |  |  |  |  |  |
|  |  |  |  |  |  |  |  |  |  |
|  |  |  |  |  |  |  |  |  |  |
|  |  |  |  |  |  |  |  |  |  |
|  |  |  |  |  |  |  |  |  |  |
|  |  |  |  |  |  |  |  |  |  |
|  |  |  |  |  |  |  |  |  |  |
|  |  |  |  |  |  |  |  |  |  |
|  |  |  |  |  |  |  |  |  |  |
| \|  \| \| --- \| |  |  |  |  |  |  |  |  |  |
|  |  |  |  |  |  |  |  |  |  |
|  |  |  |  |  |  |  |  |  |  |
|  |  |  |  |  |  |  |  |  |  |
|  |  |  |  |  |  |  |  |  |  |
|  |  |  |  |  |  |  |  |  |  |
|  |  |  |  |  |  |  |  |  |  |
|  |  |  |  |  |  |  |  |  |  |
|  |  |  |  |  |  |  |  |  |  |
|  |  |  |  |  |  |  |  |  |  |
|  |  |  |  |  |  |  |  |  |  |
|  |  |  |  |  |  |  |  |  |  |
|  |  |  |  |  |  |  |  |  |  |
|  |  |  |  |  |  |  |  |  |  |
|  |  |  |  |  |  |  |  |  |  |
|  |  |  |  |  |  |  |  |  |  |
|  |  |  |  |  |  |  |  |  |  |
|  |  |  |  |  |  |  |  |  |  |
|  |  |  |  |  |  |  |  |  |  |
|  |  |  |  |  |  |  |  |  |  |
| \|  \| \| --- \| |  |  |  |  |  |  |  |  |  |
|  |  |  |  |  |  |  |  |  |  |
|  |  |  |  |  |  |  |  |  |  |
|  |  |  |  |  |  |  |  |  |  |
|  |  |  |  |  |  |  |  |  |  |
|  |  |  |  |  |  |  |  |  |  |
|  |  |  |  |  |  |  |  |  |  |
|  |  |  |  |  |  |  |  |  |  |
|  |  |  |  |  |  |  |  |  |  |
|  |  |  |  |  |  |  |  |  |  |
|  |  |  |  |  |  |  |  |  |  |
|  |  |  |  |  |  |  |  |  |  |
|  |  |  |  |  |  |  |  |  |  |
|  |  |  |  |  |  |  |  |  |  |
|  |  |  |  |  |  |  |  |  |  |
|  |  |  |  |  |  |  |  |  |  |
|  |  |  |  |  |  |  |  |  |  |
|  |  |  |  |  |  |  |  |  |  |
|  |  |  |  |  |  |  |  |  |  |

### Mood test

| Summary statistics: | |  |  |  |  |  |  |  |  |
| --- | --- | --- | --- | --- | --- | --- | --- | --- | --- |
|  |  |  |  |  |  |  |  |  |  |
| Variable | Observations | Obs. with missing data | Obs. without missing data | Minimum | Maximum | Mean | Std. deviation |  |  |
| Delta | 14 | 0 | 14 | 31.200 | 730.080 | 190.394 | 185.800 |  |  |
| Ancestral | 14 | 0 | 14 | 1.390 | 27.460 | 9.481 | 10.254 |  |  |
|  |  |  |  |  |  |  |  |  |  |
|  |  |  |  |  |  |  |  |  |  |
| **Mood test:** | |  |  |  |  |  |  |  |  |
|  |  |  |  |  |  |  |  |  |  |
| U | 28.000 |  |  |  |  |  |  |  |  |
| Critical value | 3.841 |  |  |  |  |  |  |  |  |
| DF | 1.000 |  |  |  |  |  |  |  |  |
| p-value | < 0.0001 |  |  |  |  |  |  |  |  |
| alpha | 0.05 |  |  |  |  |  |  |  |  |
| The p-value has been computed using 10000 Monte Carlo simulations. Time elapsed: 0s. | | | | | | | | |  |
|  |  |  |  |  |  |  |  |  |  |
| Test interpretation: | |  |  |  |  |  |  |  |  |
| H0: The medians of Delta and Ancestral are equal. | | | | |  |  |  |  |  |
| Ha: Medians of Delta and Ancestral are not equal | | | | |  |  |  |  |  |
| As the computed p-value is lower than the significance level alpha=0.05, one should reject the null hypothesis H0, and accept the alternative hypothesis Ha. | | | | | | | | |  |
|  |  |  |  |  |  |  |  |  |  |
|  |  |  |  |  |  |  |  |  |  |
|  |  |  |  |  |  |  |  |  |  |
| 95% confidence interval on the p-value: | | | |  |  |  |  |  |  |
| ] 0.000, | 0.000 [ |  |  |  |  |  |  |  |  |
|  |  |  |  |  |  |  |  |  |  |

## Relative risk of exposure

### Kruskal-Wallis test

|  |  |  |  |  |  |  |  |  |  |
| --- | --- | --- | --- | --- | --- | --- | --- | --- | --- |
| Variable | Observations | Obs. with missing data | Obs. without missing data | Minimum | Maximum | Mean | Std. deviation |  |  |
| DELTA S | 14 | 0 | 14 | 1.000 | 23.400 | 6.102 | 5.955 |  |  |
| ANCESTRAL S | 14 | 0 | 14 | 1.000 | 19.755 | 6.821 | 7.377 |  |  |
|  |  |  |  |  |  |  |  |  |  |
|  |  |  |  |  |  |  |  |  |  |
| Kruskal-Wallis test / Two-tailed test: | | | |  |  |  |  |  |  |
|  |  |  |  |  |  |  |  |  |  |
| K | 0.119 |  |  |  |  |  |  |  |  |
| p-value (one-tailed) | 0.739 |  |  |  |  |  |  |  |  |
| alpha | 0.050 |  |  |  |  |  |  |  |  |
| The p-value has been computed using 10000 Monte Carlo simulations. Time elapsed: 0s. | | | | | | | | |  |
| 99% confidence interval on the p-value: | | | |  |  |  |  |  |  |
| ] 0.727, | 0.750 [ |  |  |  |  |  |  |  |  |
|  |  |  |  |  |  |  |  |  |  |
| Test interpretation: | |  |  |  |  |  |  |  |  |
| H0: The samples come from the same population. | | | | |  |  |  |  |  |
| Ha: The samples do not come from the same population. | | | | | |  |  |  |  |
| As the computed p-value is greater than the significance level alpha=0.05, one cannot reject the null hypothesis H0. | | | | | | | | |  |
|  |  |  |  |  |  |  |  |  |  |
|  |  |  |  |  |  |  |  |  |  |
|  |  |  |  |  |  |  |  |  |  |
| Ties have been detected in the data and the appropriate corrections have been applied. | | | | | | | | |  |
|  |  |  |  |  |  |  |  |  |  |
|  |  |  |  |  |  |  |  |  |  |
| Multiple pairwise comparisons using Dunn's procedure / Two-tailed test: | | | | | | | |  |  |
|  |  |  |  |  |  |  |  |  |  |
| Sample | Frequency | Sum of ranks | Mean of ranks | Groups |  |  |  |  |  |
| ANCESTRAL S | 14 | 195.500 | 13.964 | A |  |  |  |  |  |
| DELTA S | 14 | 210.500 | 15.036 | A |  |  |  |  |  |
|  |  |  |  |  |  |  |  |  |  |
|  |  |  |  |  |  |  |  |  |  |
| \|  \| \| --- \| |  |  |  |  |  |  |  |  |  |
|  |  |  |  |  |  |  |  |  |  |
|  |  |  |  |  |  |  |  |  |  |
|  |  |  |  |  |  |  |  |  |  |
|  |  |  |  |  |  |  |  |  |  |
|  |  |  |  |  |  |  |  |  |  |
|  |  |  |  |  |  |  |  |  |  |
|  |  |  |  |  |  |  |  |  |  |
|  |  |  |  |  |  |  |  |  |  |
|  |  |  |  |  |  |  |  |  |  |
|  |  |  |  |  |  |  |  |  |  |
|  |  |  |  |  |  |  |  |  |  |
|  |  |  |  |  |  |  |  |  |  |
|  |  |  |  |  |  |  |  |  |  |
|  |  |  |  |  |  |  |  |  |  |
|  |  |  |  |  |  |  |  |  |  |
|  |  |  |  |  |  |  |  |  |  |
|  |  |  |  |  |  |  |  |  |  |
|  |  |  |  |  |  |  |  |  |  |
| \|  \| \| --- \| |  |  |  |  |  |  |  |  |  |
|  |  |  |  |  |  |  |  |  |  |
|  |  |  |  |  |  |  |  |  |  |
|  |  |  |  |  |  |  |  |  |  |
|  |  |  |  |  |  |  |  |  |  |
|  |  |  |  |  |  |  |  |  |  |
|  |  |  |  |  |  |  |  |  |  |
|  |  |  |  |  |  |  |  |  |  |
|  |  |  |  |  |  |  |  |  |  |
|  |  |  |  |  |  |  |  |  |  |
|  |  |  |  |  |  |  |  |  |  |
|  |  |  |  |  |  |  |  |  |  |
|  |  |  |  |  |  |  |  |  |  |
|  |  |  |  |  |  |  |  |  |  |
|  |  |  |  |  |  |  |  |  |  |
|  |  |  |  |  |  |  |  |  |  |
|  |  |  |  |  |  |  |  |  |  |
|  |  |  |  |  |  |  |  |  |  |
| \|  \| \| --- \| |  |  |  |  |  |  |  |  |  |
|  |  |  |  |  |  |  |  |  |  |
|  |  |  |  |  |  |  |  |  |  |
|  |  |  |  |  |  |  |  |  |  |
|  |  |  |  |  |  |  |  |  |  |
|  |  |  |  |  |  |  |  |  |  |
|  |  |  |  |  |  |  |  |  |  |
|  |  |  |  |  |  |  |  |  |  |
|  |  |  |  |  |  |  |  |  |  |
|  |  |  |  |  |  |  |  |  |  |
|  |  |  |  |  |  |  |  |  |  |
|  |  |  |  |  |  |  |  |  |  |
|  |  |  |  |  |  |  |  |  |  |
|  |  |  |  |  |  |  |  |  |  |
|  |  |  |  |  |  |  |  |  |  |
|  |  |  |  |  |  |  |  |  |  |
|  |  |  |  |  |  |  |  |  |  |
|  |  |  |  |  |  |  |  |  |  |
|  |  |  |  |  |  |  |  |  |  |
|  |  |  |  |  |  |  |  |  |  |
| \|  \| \| --- \| |  |  |  |  |  |  |  |  |  |
|  |  |  |  |  |  |  |  |  |  |
|  |  |  |  |  |  |  |  |  |  |
|  |  |  |  |  |  |  |  |  |  |
|  |  |  |  |  |  |  |  |  |  |
|  |  |  |  |  |  |  |  |  |  |
|  |  |  |  |  |  |  |  |  |  |
|  |  |  |  |  |  |  |  |  |  |
|  |  |  |  |  |  |  |  |  |  |
|  |  |  |  |  |  |  |  |  |  |
|  |  |  |  |  |  |  |  |  |  |
|  |  |  |  |  |  |  |  |  |  |
|  |  |  |  |  |  |  |  |  |  |
|  |  |  |  |  |  |  |  |  |  |

### Mood Test

| Summary statistics: | |  |  |  |  |  |  |  |  |
| --- | --- | --- | --- | --- | --- | --- | --- | --- | --- |
|  |  |  |  |  |  |  |  |  |  |
| Variable | Observations | Obs. with missing data | Obs. without missing data | Minimum | Maximum | Mean | Std. deviation |  |  |
| DELTA S | 14 | 0 | 14 | 1.000 | 23.400 | 6.102 | 5.955 |  |  |
| ANCESTRAL S | 14 | 0 | 14 | 1.000 | 19.755 | 6.821 | 7.377 |  |  |
|  |  |  |  |  |  |  |  |  |  |
|  |  |  |  |  |  |  |  |  |  |
| **Mood test:** | |  |  |  |  |  |  |  |  |
|  |  |  |  |  |  |  |  |  |  |
| U | 0.571 |  |  |  |  |  |  |  |  |
| Critical value | 3.841 |  |  |  |  |  |  |  |  |
| DF | 1.000 |  |  |  |  |  |  |  |  |
| p-value | 0.701 |  |  |  |  |  |  |  |  |
| alpha | 0.05 |  |  |  |  |  |  |  |  |
| The p-value has been computed using 10000 Monte Carlo simulations. Time elapsed: 0s. | | | | | | | | |  |
|  |  |  |  |  |  |  |  |  |  |
| Test interpretation: | |  |  |  |  |  |  |  |  |
| H0: The medians of DELTA S and ANCESTRAL S are equal. | | | | | |  |  |  |  |
| Ha: Medians of DELTA S and ANCESTRAL S are not equal | | | | | |  |  |  |  |
| As the computed p-value is greater than the significance level alpha=0.05, one cannot reject the null hypothesis H0. | | | | | | | | |  |
|  |  |  |  |  |  |  |  |  |  |
|  |  |  |  |  |  |  |  |  |  |
|  |  |  |  |  |  |  |  |  |  |
| 95% confidence interval on the p-value: | | | |  |  |  |  |  |  |
| ] 0.692, | 0.710 [ |  |  |  |  |  |  |  |  |
|  |  |  |  |  |  |  |  |  |  |
